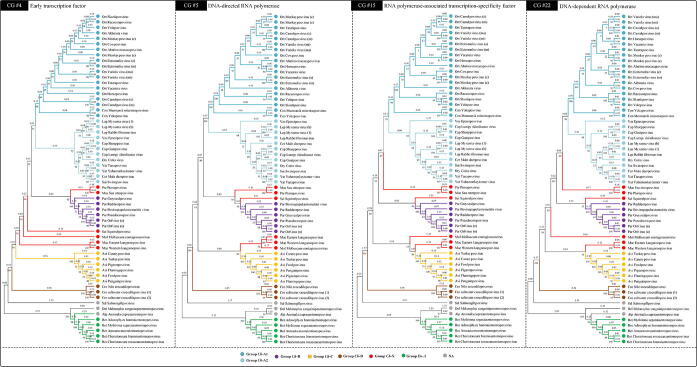# Genomic analysis of Poxviridae and exploring qualified gene sequences for phylogenetics

**DOI:** 10.1016/j.csbj.2021.09.031

**Published:** 2021-09-28

**Authors:** Zehui Yu, Wenjie Zhang, Huancheng Fu, Xiaoxia Zou, Mingde Zhao, Sicheng Liang, Congwei Gu, Qian Yang, Manli He, Qihai Xiao, Wudian Xiao, Lvqin He, Muhan Lü

**Affiliations:** a Laboratory Animal Center, Southwest Medical University, Luzhou, Sichuan, PR China; b Department of Gastroenterology, The Affiliated Hospital of Southwest Medical University, Luzhou, Sichuan Province, PR China; c School of Basic Medical Sciences, Zhejiang University, Hangzhou, PR China; d School of Basic Medical Sciences, Zunyi Medical University, Zunyi, Guizhou, PR China; e State Key Laboratory of Biotherapy and Cancer Center, Sichuan University, Sichuan, PR China; f Suining First People's Hospital, Sichuan, PR China

**Keywords:** *Poxviridae*, Core genes, Synteny analysis, Phylogenetics

## Abstract

The members of the *Poxviridae* family are globally distributed all over the world and can cause infectious diseases. Although genome sequences are publicly available for representative isolates of all genera, studies on the criteria for genome-based classification within the *Poxviridae* family have rarely been reported. In our study, 60 *Poxviridae* genomes were re-annotated using Prokka. By using BLAST filtration and MCScanX, synteny and similarity of whole genomic amino acid sequences were visualized. According to the analysis pattern, the *Chordopoxvirinae* and *Entomopoxvirinae* subfamilies can be subdivided into five and two categories respectively, which is consistent with the phylogenetic tree constructed based on whole genomic amino acid sequences and Poxvirus core genes. Finally, four genes (Early transcription factor, DNA-directed RNA polymerase, RNA polymerase-associated transcription-specificity factor and DNA-dependent RNA polymerase) were selected from Poxvirus core genes by substitution saturation analysis and phylogenetic tree verification. Phylogenetic trees constructed based on single gene and concatenated sequences of the four selected genes showed that the classification of subgroups was consistent with the phylogenetic trees based on genome. Conclusion: a new method based on the similarity of whole genomic amino acid sequences was proposed for *Poxviridae* taxon demarcation, and the use of the four selected qualified genes will help make phylogenic identification of newly discovered *Poxviridae* isolates more convenient and accurate.

## Graphical abstract

**Figure:**
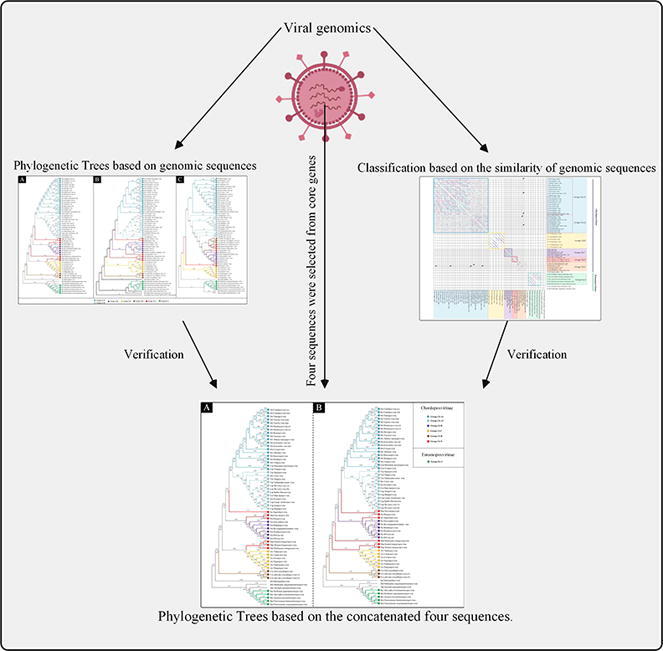

## 1 Introduction

The family *Poxviridae*, belonging to a group of large eukaryotic dsDNA viruses termed Nucleo-Cytoplasmic Large DNA Viruses (NCLDVs), has been found to infect a diverse array of birds, mammals and insects. According to the latest International Committee on Taxonomy of Viruses (ICTV) Master Species List 2020.v1, the family *Poxviridae* contains two subfamilies (*Chordopoxvirinae* and *Entomopoxvirinae*) and is currently subdivided into 22 genera. The following criteria: phylogenetic analysis, nucleotide sequence or amino acid identity, gene content comparisons, organization of the genome, growth characteristics and host range in cell culture, disease characteristics, and serological criteria, are used as a guideline to establish the taxonomic statuses of species, genera, and subfamilies within the *Poxviridae* family (ICTV assigned code 2019.005D). Among them, phylogenetic distance and natural host are the primary criteria. Though these methods can characterize the evolutionary relationship through the classification of poxviruses, clear division criteria at genus level are lacking. For example, the genus demarcation criterion for the family *Iridoviridae* is that members of a given genus share less than 50% amino acid sequence identity with members of other genera (ICTV assigned code 2018.007D). However, such clear division criteria as seen for the family *Iridoviridae* (ICTV assigned code 2019.003G, 2019.005D and 2020.001G), is lacking for the family *Poxviridae*. In addition, the classifications of the family *Poxviridae* into subfamilies and genera are mainly based on phylogenetic analysis and host range [1]. However, with the discovery of newly isolated poxviruses, it is difficult to reconcile these classification methods. For example, while viruses of the same genus can infect different hosts, as seen in the genus *Orthopoxvirus*, viruses divided into different genera can also infect the same host, as seen by Vaccinia virus (*Orthopoxvirus*) and Molluscum contagiosum virus (*Molluscipoxvirus*) both infecting human (Table 3).

Poxvirus genomes contain linear double stranded DNA ranging from 130 kbp in parapoxviruses to 380 kbp in entomopoxviruses and the coding potential of poxvirus genomes ranges from approximately 133 genes in parapoxviruses and yatapoxviruses to 328 genes in canarypox virus. With the development of genomic sequencing technology, it has become more convenient and quicker to obtain complete virus sequences. To date, the complete genomic sequences of most viruses within the family *Poxviridae* have already been published in NCBI (https://www.ncbi.nlm.nih.gov). These published genomic sequences can provide the fundamental database for studying the evolution and taxonomy of the family *Poxviridae*. In this study, we propose a novel poxvirus taxon demarcation based on the similarity of genomic amino acid sequences and genomic synteny. In addition, four qualified genes for phylogenetic analysis were selected from poxvirus core genes, which can be beneficial in phylogenic identification of newly discovered poxvirus isolates.

## 2 Materials and methods

### 2.1 Genome and re-annotation

A total of 60 poxvirus genomic nucleic acid sequences were obtained from National Center for Biotechnology Information (NCBI, www.ncbi.nlm.nih.gov/). The detailed information about host species, the country of origin and the year of detection are listed in Table S1. To avoid different genomic annotation method leading to deviation in subsequent analysis, we used the Prokka v1.14.5 [2] to annotate the 60 genomes uniformly using the same parameters (settings: --kingdom Viruses, remaining settings: default).

### 2.2 Synteny analysis

BLAST v2.6.0+ [3] and MCScanX [4] were performed to determine synteny between 60 poxviruses (Table 1). Firstly, the database was built by merging 60 annotated amino acid sequence files generated by Prokka software and using “makeblast” command of BLAST (Step 1: Merge the 60 poxviruses amino acid sequences into an all.fa input file). Secondly, the merged sequence file was aligned by using “blastp” command of BLAST (Step 2: Perform mutual BLAST alignment of all amino acid sequences in the 60 poxviruses). Then, the results of the comparison are filtered according to the identity threshold set as 30%, 70% and 85% respectively (Step 3). Finally, both the annotation information file (gff format) and aligned file were imported into MCScanX to generate synteny images (Step 4 and Step5).

**Table 1:** The detailed steps of synteny analysis.

Step	Codes
Step 1: Create database	makeblastdb -in poxviruse.fa -dbtype prot -out index/all -parse_seqids
Step 2: BLAST	blastp -query poxviruse.fa -db index/all -out out.blast -evalue 1e-5 -num_threads 8 -outfmt 6
Step 3: Filtration	cat out.blast | awk ‘{ if ($3 > 30) print $0}’ > poxviruse.blast (identity threshold set as 30%) cat out.blast | awk ‘{ if ($3 > 70) print $0}’ > poxviruse.blast (identity threshold set as 70%) cat out.blast | awk ‘{ if ($3 > 85) print $0}’ > poxviruse.blast (identity threshold set as 85%)
Step 4: MCScanX	./MCScanX input_file/poxviruse
Step 5: Visualization	java dot_plotter -g poxviruse.gff -s poxviruse.collinearity -c dot.ctl -o dot.PNG

### 2.3 Core-pan analysis

The strictly core genes (present in all viral genomes) of the 60 *Poxviridae* genomes were identified by using PanX [5]. The input files generated by Prokka software (settings: --cg 1.0, --nsl; remaining settings: default) were in “gbk” format. The identified strictly core genes would be used in subsequent analysis and also used to explore which genes are qualified for use in phylogenetic analysis.

### 2.4 Phylogenetic analysis

The composition vector phylogenetic tree (CV-Tree) is an alignment-free classification tools based on whole-genome [6], [7]. The amino acid sequences generated by Prokka were directly submitted to the CVTree3 Web Server (http://tlife.fudan.edu.cn/cvtree/cvtree/, K-tuple length was set at 5, Select Built-In Genomes: none). The maximum likelihood phylogenetic tree (ML-Tree) was constructed based on poxvirus core genes. MAFFT software was used to align the core genes sequences identified by PanX [8] and the aligned core genes were concatenated in order using PhyloSuite [9]. The ML-Tree was then constructed by using MEGA-X [10]. Detailed parameter settings (refer to ICTV Proposal, https://talk.ictvonline.org/taxonomy/p/taxonomy-history?taxnode_id = 202007155) are shown in Table 2. The single nucleotide polymorphisms tree (SNPs-Tree) was generated automatically by PanX [5]. The annotations for the phylogenetic trees were made using Visio 2016 as we performed recently [27], [28].

**Table 2:** The parameter settings for phylogenetic analysis.

	Items	Setting
ML-Tree	Phylogeny Test	Test of Phylogeny: Bootstrap method No.: 100
	Substitution Model	Substitutions Type: Nucleotide/Amino acid Model/Method: Tamura-Nei model/LG model
	Rates and Patterns	Rates among Sites: Gamma Distributed (G) No of Discrete Gamma Categories: 5
	Data Subset to Use	Gaps/Missing Data Treatment: Partial deletion Site Coverage Cutoff (%): 95
	Tree Inference Options	ML Heuristic Method: Nearest-Neighbor-Interchange (NNI) Initial Tree for ML: automatically (Maximum Parsimony) Branch Swap Filter: None
NJ-Tree	Phylogeny Test	Test of Phylogeny: Bootstrap method No.: 1000
	Substitution Model	Substitutions Type: Nucleotide/Amino acid Model/Method: Maximum Composite Likelihood/Poisson Substitutions to Include: Transitions + Transversions/-
	Rates and patterns	Rates among Sites: Uniform Rates Pattern among Lineages: Same (Homogeneous)
	Data Subset to Use	Gaps/Missing Data Treatment: Complete deletion Select Codon Positions: all

**Table 3:** Host range and taxonomic classification of the family *Poxviridae* based on synteny analysis.

30% threshold	70% threshold	Virus genera	Host range [15], [16]
Group *Ch*	Group *Ch-A*	*Orthopoxvirus* (A1)	Mammalian species (including human, monkey and cow *etc*.)
		*Centapoxvirus* (A1)	*Microtus oeconomus*
		*Cervidpoxvirus* (A2)	Mule deer
		*Vespertilionpoxvirus* (A2)	Bat
		*Suipoxvirus* (A2)	Swine
		*Capripoxvirus* (A2)	Sheep
		*Yatapoxvirus* (A2)	Primate
		*Oryzopoxvirus* (A2)	Mice
		*Leporipoxvirus* (A2)	Rabbit
	Group *Ch-B*	*Avipoxvirus*	Birds
	Group *Ch-C*	*Parapoxvirus*	Bovine, sheep, seal, deer and human *etc*
	Group *Ch-D*	*Crocodylidpoxvirus*	Crocodile
	Group *Ch-X*	*Macropopoxvirus*	Kangaroo
		*Sciuripoxvirus*	Squirrel
		*Pteropopoxvirus*	Fox
		*Mustelpoxvirus*	Sea
		*Molluscipoxvirus*	Human
		*Salmonpoxvirus*	Fish
Group *En*	Group *En-A*	*Betaentomopoxvirus*	Lepidoptera and Orthoptera
		*Alphaentomopoxvirus* *Deltaentomopoxvirus*	Coleoptera Melanoplus sanguinipes

### 2.5 Exploring qualified sequences from core genes

The sequences that had experienced severe substitution saturation [11] or recombination [12], [13] were not suitable for phylogenetic analysis [11], [12], [13]. In order to explore qualified genes from core genes, recombination analysis and substitution saturation analysis of Poxvirus core genes were performed using Recombination Detection Program (RDP) BETA4.67 [13] and DAMBE v5.3.19 [11]. For recombination analysis, the identified core genes sequences were aligned using MAFFT software and the aligned core genes were concatenated in order using PhyloSuite [9]. The concatenated core genes file was imported into RDP software to perform recombination analysis. For substitution saturation analysis, the aligned gene sequences from the core genes were imported separately to DAMBE. In the “Seq.analysis” drop-down list, the “Measure Substitution Saturation > Test by Xia et al.” option was chosen to perform substitution saturation analysis. The detailed steps have been outlined in our previous research paper [12]. The NJ-Trees and ML-Trees based on single core gene were constructed using MEGA-X [10] (detailed parameter settings are shown in Table 2).

## 3 Results

### 3.1 Synteny analysis

**Fig. 1:**
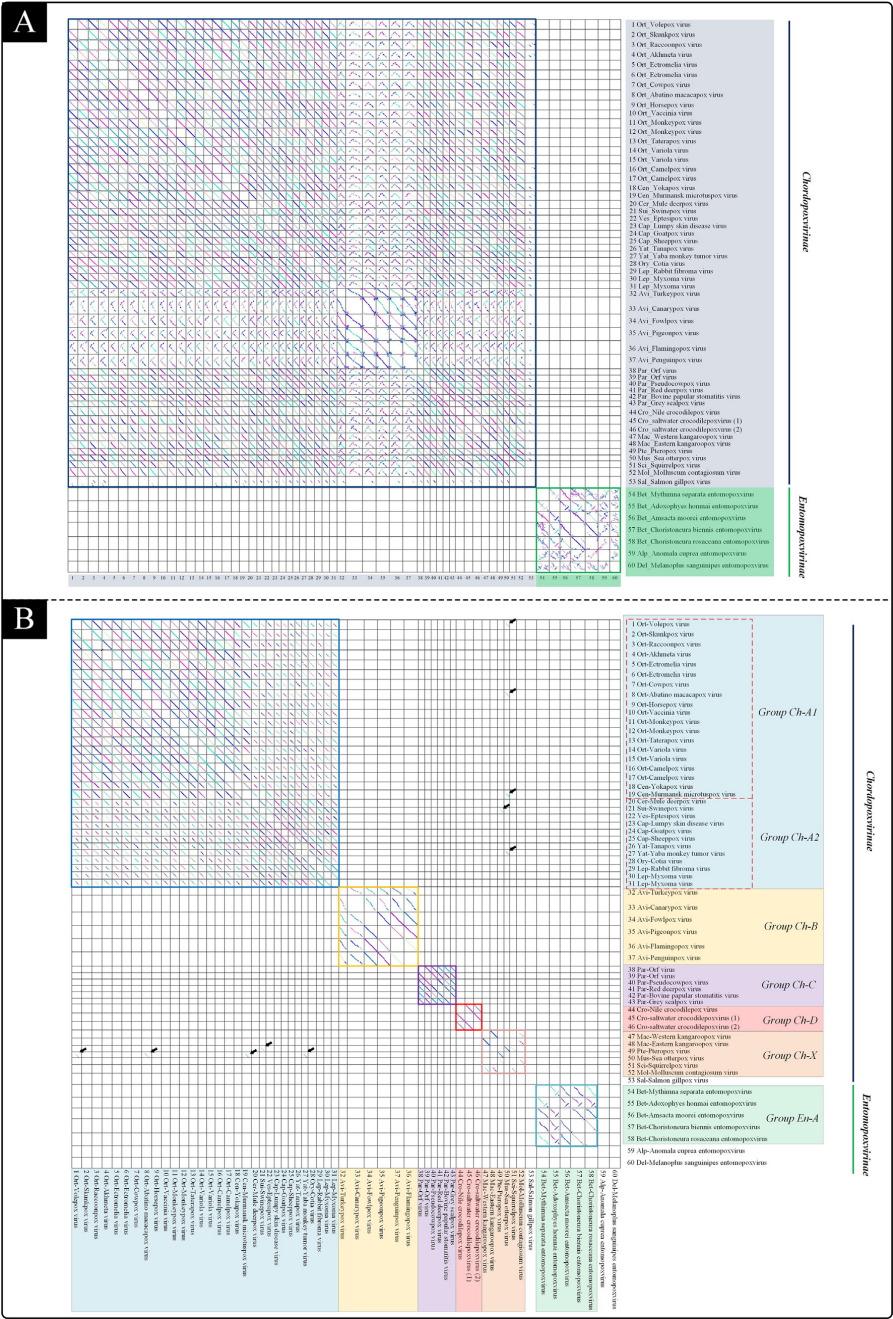
Synteny analysis of 60 members in *Poxviridae* family (A: identity threshold set as 30%, B: identity threshold set as 70%). The first three letters are abbreviated from the genus name (e.g. Ort-Volepox virus, Ort means *Orthopoxvirus*). Each corresponding block represents the collinearity comparison of two viruses. If there is no collinearity amino acid sequence between two viruses at 30% (Fig. 1A) and 70% (Fig. 1B) identity level, the block would be blank. The different colors of the boxes and shaded panels represent manual grouping; For example in Fig. 1A, all viruses in *Entomopoxvirinae* Group share > 30% BLAST identity collinearity sequences with each other (green box), but not with viruses from other groups as seen by the corresponding blank boxes. The colours of lines in a block are to distinguish between different collinear regions. The colours between blocks are irrelevant. (For interpretation of the references to color in this figure legend, the reader is referred to the web version of this article.)

Synteny analysis may serve as an alternative method to determine viral taxonomy and evolutionary relationship, such as in the case of the family *Iridoviridae* [14]. MCScanX [4] is a visual tool used in identifying gene order and comparing genomic structural changes. In our study, the genomic linear relationships of 60 poxviruses were compared in pairs by using BLAST and MCScanX software and the corresponding collinearity sequences, if any, between each pair of sequences are shown in the corresponding block (Fig. 1 A and B).

In this study, poxviruses were grouped according to the presence or absence of collinearity amino acid sequence at different BLAST identity levels. After screening with the identity threshold set as 30% (meaning that sequences with less than 30% BLAST identity were filtered out), the 60 poxviruses were divided into two groups (Fig. 1A): *Chordopoxvirinae* subfamily group (abbreviated to “Group *Ch*”) and *Entomopoxvirinae* subfamily group (abbreviated to “Group *En*”).

**Supplementary figure 1:**
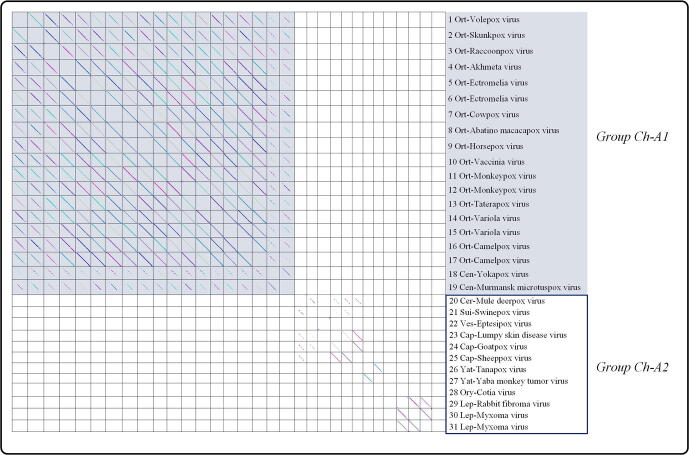


By setting the identity threshold as 70% (meaning that sequences with less than 70% BLAST identity were filtered out), the Group *Ch* was then further subdivided into five categories and Group *En* into one category (Fig. 1B and Table 3). Notably, Salmon gill poxvirus (*Salmonpoxvirus*), Anomala cuprea entomopoxvirus (*Alphaentomopoxvirus*) and Melanoplus sanguinipes entomopoxvirus (*Deltaentomopoxvirus*) shared no collinearity with any other viruses. The Groups *Ch*-A, *Ch*-B, *Ch*-C, *Ch*-D and *En*-A shared > 70% BLAST identity collinearity sequences with only the viruses within the same group (blocks corresponding to collinearity with other groups were all blank). Furthermore, the Group *Ch*-A could be subdivided into 2 subgroups when the identity threshold was set as 85% (Supplementary fig. 1). It is also worth noting that Group *Ch*-X is a special group. The viruses in Group *Ch*-X were only sporadically (not all) collinear with the viruses in the same group, unlike Groups *Ch*-A, *Ch*-B, *Ch*-C, *Ch*-D and *En*-A, where all viruses in the same group were collinear to each other. In addition, the sea otterpox virus(n = 4) and pteropox virus(n = 1) in Group *Ch*-X also shared > 70% BLAST identity collinearity sequences with other groups of viruses (Fig. 1B, black arrow).

### 3.2 Defining the core genes

The result of core-pan analysis by using PanX showed that the 60 poxviruses shared 22 strictly core genes (shared by all viruses). The locations and annotation information of these 22 Poxvirus core genes are summarized in Table S2. In 2003, Upton, Chris, et al. defined 49 core genes in 21 poxvirus genomes [17]. The smaller number of core genes defined in our study is due to the different methods of defining the core genes.

B2L gene [18], [19], P32 gene [20], [21], fpv167 gene [22], DNA topoisomerase I and DNA polymerase [23] have been previously used as phylogenetic markers. DNA topoisomerase I and DNA polymerase are within the 22 core genes identified in our study, and correspond to CG#16 and CG#22, respectively (Table S2, CG is short for core gene). However, B2L gene, P32 gene and fpv167 gene cannot be detected using panX in *Entomopoxvirinae* subfamily.

### 3.3 Phylogenetic analysis

**Fig. 2:**
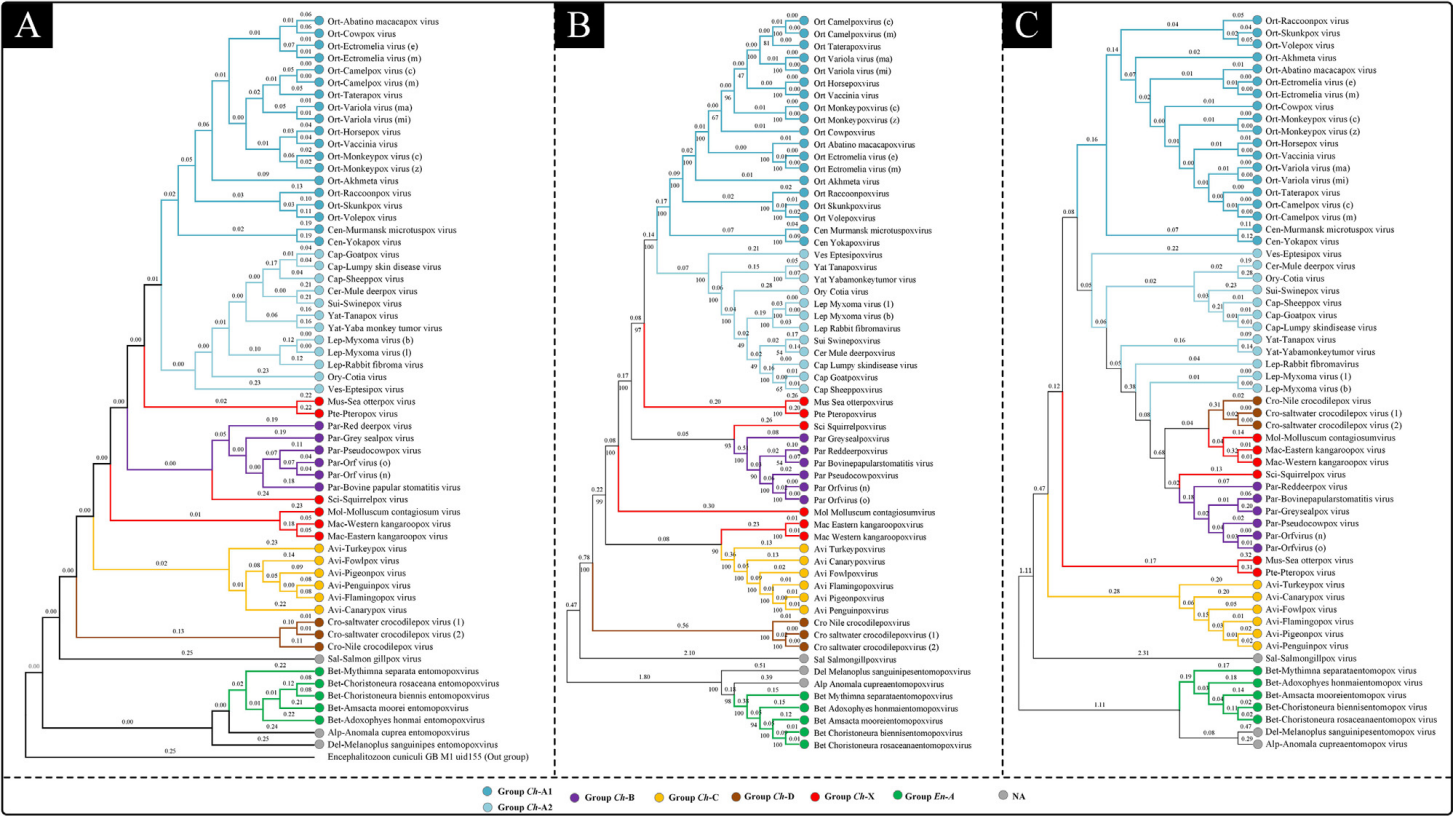
Phylogenetic analysis of 60 poxviruses. (A) The composition vector phylogenetic tree (CV-Tree) based on genomic amino acid sequences. (B) The maximum likelihood phylogenetic tree (ML-Tree) based on poxvirus core genes. (C) The single nucleotide polymorphisms phylogenetic tree (SNP-Tree) based on poxvirus core genes. The numbers on the branches represent branch lengths/genetic distances and numbers below the branch points represent bootstrap values. The color of the branch endpoints represents the classification results based on synteny analysis.

Genomic amino acid sequences were used to construct the CV-Tree using the composition vector method. Meanwhile, the ML-Tree and SNPs-Tree were constructed based on the defined 22 Poxvirus core genes using MAGE and PanX software. Group classification based on synteny analysis was markedly distinct in the phylogenetic trees. All the phylogenetic trees showed that despite making use of different types of sequences and phylogenetic algorithms, viruses in Group *En* still separately clustered into a single branch (Fig. 2). The ML-Tree and CV-Tree were found to be the most consistent with the result of synteny analysis. All the viruses in Groups *Ch-*A, *Ch-*B, *Ch-*C, *Ch-*D and *En-*A were clustered into the same branch (Fig. 2A and B). In addition, similar to what was seen in synteny analysis, Group *Ch-*X in phylogenetic trees also displayed unique characteristics. All three phylogenetic trees showed that the viruses in Group *Ch-*X were interspersed among other Groups *Ch*. In the grouping using synteny analysis, only some viruses in Group *Ch*-X were collinear with the viruses in the same group (the internal evolutionary relationship is weak), while other viruses shared collinearity sequences with viruses from other groups (Fig. 1B, black arrow). This may lead to Group *Ch*-X not forming monophyletic clades in the phylogeny. In general, the grouping results based on synteny analysis were consistent with the genomic phylogenetic analysis.

### 3.4 Exploring qualified sequences

#### 3.4.1 Comparison of nucleotide and amino acid sequences

**Fig. 3:**
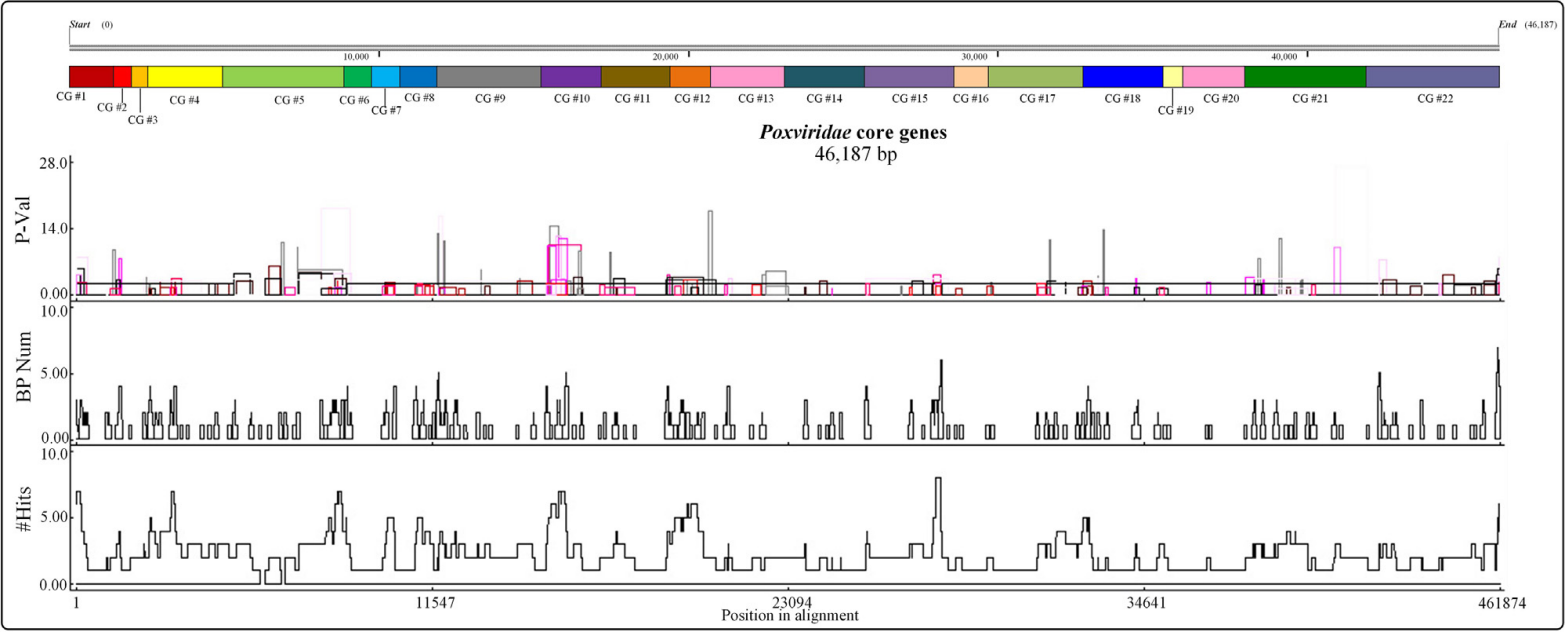
The recombination analysis detected by RDP4 among 22 Poxvirus core genes. The color bands at the top of the image indicate the placement of core genes. The abscissa represents nucleotide position of core genes. The ordinate represents the minimum probability values associated with detected events (P-Val), recombination breakpoint numbers (BP Num) and the number of events detected in particular regions of the alignment (#Hits).

In order to verify what type of sequence (nucleotide and amino acid sequences) is suitable for phylogenetic analysis, NJ-Trees and ML-Trees based on each core gene were constructed. Our results showed that most of the NJ-Trees and ML-Trees constructed based on the nucleotide sequences of core genes were incorrect (Supplementary file 1 and 2) and could not even distinguish between the subfamilies *Entomopoxvirinae* and *Chordopoxvirinae* (the phylogenetic trees incorrectly mark Group *En* in yellow in Supplementary file 1 and 2). Phylogenetic trees based on amino acid sequences yielded better results than those using nucleotide sequences (Supplementary file 3 and 4). We suspected that this may be due to recombinant fragments within the core genes since recombination analysis revealed that all core genes contained recombinant fragments (Fig. 3 and Table S4). Such recombination events could severely decrease the accuracy of phylogenetic trees [12], [13]. However, further algorithmic research is still needed to determine the influence of recombination events on the construction of phylogenetic trees based on amino acid sequences.

#### 3.4.2 Substitution saturation analysis

The accuracy of a phylogenetic tree can be influenced by whether the sequences have experienced substantial substitution saturation [24]. In order to determine which sequences from core genes are qualified for phylogenetics, we used DAMBE7 software to assess the substitution saturation of Poxvirus core genes [25]. The values of *Iss* (index of substitution saturation), *Iss.c* (critical *Iss*) and P-value are shown in Table S3. If *Iss* values for all subsets of NumOTU are not significantly smaller than the corresponding *Iss.c*, that means the sequences experience substantial substitution saturation and are poor choices for phylogenetic analysis. The substitution saturation analysis revealed that a total of 7 core genes (CG #2, #6, #8, #10, #11, #12 and #13) were not qualified for phylogenetic analysis (Table S3). In addition, we also performed substitution saturation analysis on the genes previously used for phylogenetic analysis (B2L, P32, fpv167, DNA topoisomerase I and DNA polymerase). Among them, P32 experience substantial substitution saturation.

#### 3.4.3 NJ-Trees and ML-Trees verification

**Supplementary figure 2:**
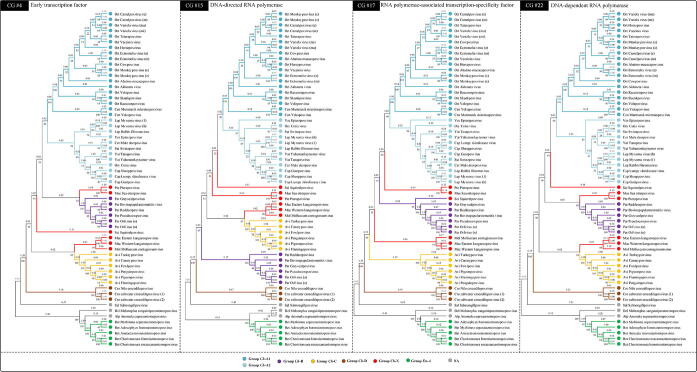


According to the previous results of synteny and genomic phylogenetic analyses, we considered the a phylogenetic tree to be qualified based on the following criteria: (1) the tree can accurately divide the poxviruses into either the *Entomopoxvirinae* or *Chordopoxvirinae* subfamily group; (2) the viruses in Groups *Ch*-A, *Ch*-B, *Ch*-C and *Ch*-D each cluster into their respective separate branch and evolutionary relationship is consistent with the genomic phylogenetic tree (Fig. 2 A and B); (3) the viruses in Groups *Ch*-A1 and *Ch*-A2 cluster into separate branches. After screening, phylogenetic trees based on amino acid sequences showed that the trees (both NJ-Tree and ML-Tree) constructed using CG #4 (Early transcription factor), CG #5 (DNA-directed RNA polymerase), CG #15 (RNA polymerase-associated transcription-specificity factor) and CG #22 (DNA-dependent RNA polymerase) met the above requirements (Table 4, the amino acid phylogenetic trees based on four qualified genes are shown in Supplementary figure 2, Supplementary figure 3, the amino acid phylogenetic trees based on 22 core genes are shown in Supplementary figs. 3 and 4). It is worth noting that using the NJ method yielded more qualified trees than the ML method (Table 4, 6 qualified NJ-Trees and 4 qualified ML-Trees). Therefore, in combination with our previous studies, it is recommended to use amino acid sequences to construct NJ-trees for poxviruses.

**Table 4:** The result of NJ-Trees and ML-Trees verification.

	NJ-Trees	ML-Trees		NJ-Trees	ML-Trees
CG #1	Type II	Type II	CG #12*	Type I	Type I
CG #2*	Type I	Type I	CG #13*	Type I	Type I
CG #3	Type II	Type II	CG #14	Type III	Type III
CG #4	Qualified	Qualified	CG #15	Qualified	Qualified
CG #5	Qualified	Qualified	CG #16	Type III	Type I
CG #6*	Type I	Type III	CG #17	Qualified	Type III
CG #7	Type II	Type II	CG #18	Type III	Type III
CG #8*	Type I	Type I	CG #19	Type II	Type II
CG #9	Type III	Type III	CG #20	Qualified	Type IV
CG #10*	Type II	Type II	CG #21	Type III	Type IV
CG #11*	Type II	Type II	CG #22	Qualified	Qualified

The sequences experienced substantial substitution saturation are indicated with “*”.

Phylogenetic trees that did not meet the requirements of qualified tree were divided into four levels according to the degree of error. Type I (subfamily level error): Salmon gill poxvirus was clustered into Group *En*. Type II (subfamily level error and degree of error is less than Type I): Salmon gill poxvirus was on a single branch and did not get clustered into Group *Ch*. Type III (genus level error): evolutionary relationship of Crocodylidpoxvirus was not consistent with the genomic phylogenetic tree. Type IV (group level error): Eptesipox virus did not cluster into Group *Ch*-A2. The verification according to the above criteria are summarized in Table 4. The result showed that all phylogenetic trees based on sequences that had experienced substantial substitution saturation were not qualified (Table 4 *). Moreover, most of the errors were relatively serious subfamily level errors (Table 4 *).

### 3.5 Phylogenetic analysis of qualified core genes

**Fig. 4:**
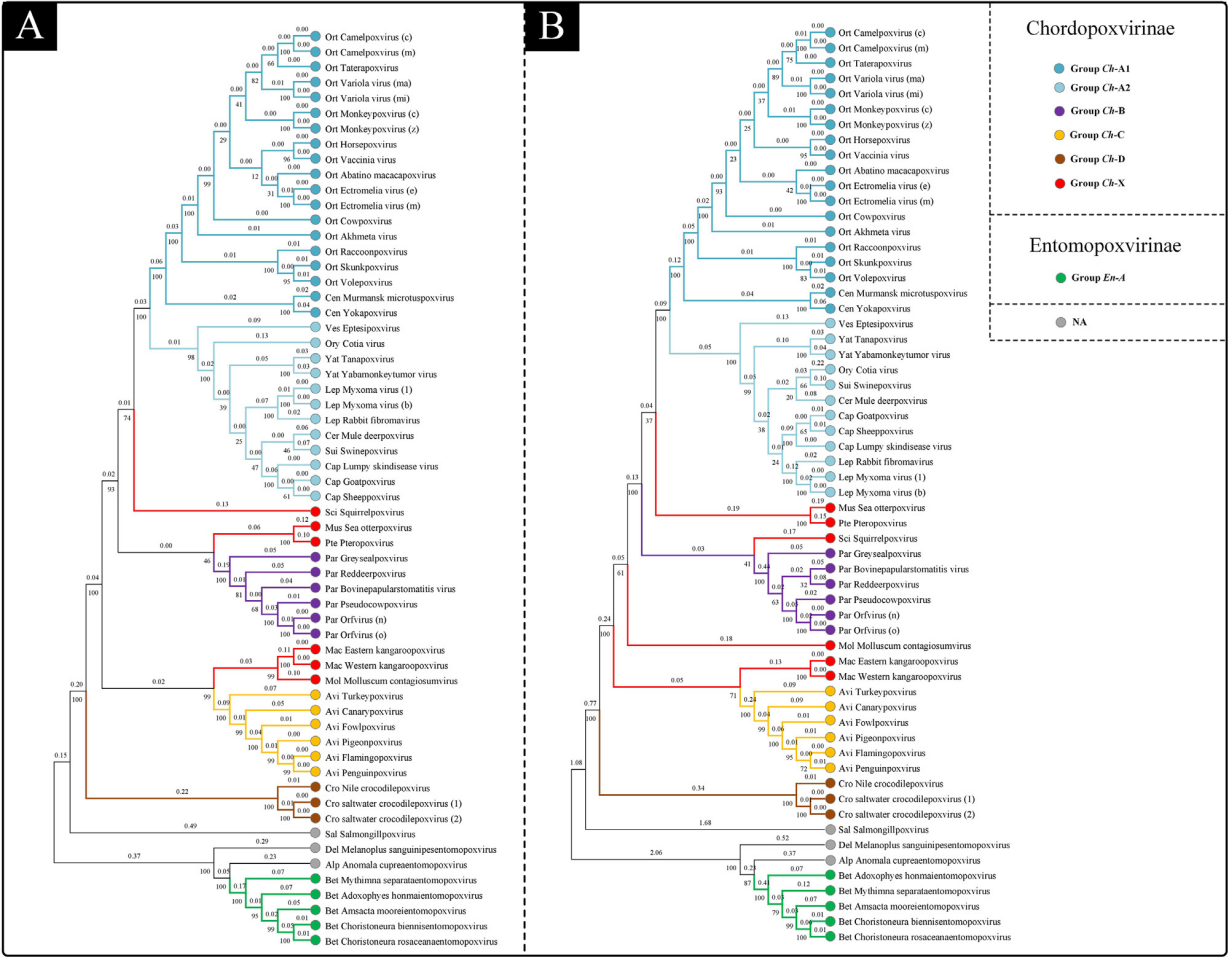
Phylogenetic analysis of 60 poxviruses based on the concatenated 4 amino acid sequences. (A) The maximum likelihood phylogenetic tree (ML-Tree). (B) The neighbor joining phylogenetic tree (NJ-Tree). The numbers on branch represent branch length/genetic distance, the numbers below the branch points represent bootstrap values.

The CG #4, #5, #15 and #22 were selected from 22 Poxvirus core genes following the substitution saturation analysis and NJ/ML-Trees verification. Phylogenetic trees were then constructed based on the concatenated four amino acid sequences (Fig. 4). The NJ-Tree and ML-Tree based on these concatenated 4 sequences showed that the branching structure was very similar to the CV-tree based on genome (Fig. 2A) and the ML-Tree based on the 22 Poxvirus core genes (Fig. 2B). The two phylogenetic trees were also considered to be qualified according to our criteria.

## 4 Discussion

According to the newest description by International Committee on Taxonomy of Viruses (ICTV) for the family *Poxviridae* (file code:2019.005D), phylogenetic distance and natural host are the primary taxon demarcation criteria. In addition, the organization of the genome was also mentioned as an optional criterion, but conservation of gene synteny can frequently be so high that the resolving power is not sufficient to distinguish between taxa. In this study, we have used BLAST filtration and MCScanX (synteny visualization software) in a novel way to solve this problem. The principle of our method was based on genomic synteny relationships and the similarity of whole genomic amino acid sequences. For example, when setting identity threshold as 30% while performing BLAST, the corresponding box would be blank if all amino acid sequences between the two virus genomes share less than 30% identity. In this way, we can distinguish between the *Entomopoxvirinae* subfamily and *Chordopoxvirinae* subfamily (Fig. 1A). By further setting the identity threshold as 70% (Fig. 1B), the *Entomopoxvirinae* and *Chordopoxvirinae* subfamilies would be subdivided into different groups and the group demarcation formed using this method would also be consistent with phylogenetic trees based on Poxvirus core genes and whole genomic amino acid sequences (Fig. 2), as well as previously published poxvirus phylogenetic analyses (ICTV assigned code 2019.007D), thus supporting the credibility of this method. To the best of our knowledge, this is the first application of such a method in the study of virus taxonomy.

Phylogenetic analysis is the primary and most common taxon demarcation criteria. Phylogenetic trees based on genome or virus core genes are accurate, but the prerequisite is that the virus genomes have been previously sequenced. Generally, taxonomic classification of newly discovered *Poxviridae* isolates are based on single or multiple viral genes, such as B2L gene [18], [19], P32 gene [20], [21], fpv167 gene [22], DNA polymerase and DNA topoisomerase I [23]. While analysis of the single gene is convenient, the phylogenetic tree based on a single gene may be not consistent with viral evolution. In this study, four genes were selected from Poxvirus core genes by substitution saturation analysis and phylogenetic tree verification. The phylogenetic tree verification result for the entire protein-coding region for the four genes indicated that all phylogenetic trees based on the single amino acid sequence (Figure S2 and S3) and also those based on the concatenated four amino acid sequences (Fig. 4) share similarity with genomic phylogenetic trees. Thus, our study can provide a valuable reference for *Poxviridae* taxonomic classification based on single gene phylogenetic analysis. The amino acid and nucleotide sequences of the selected four genes are provided in the Supplementary file. In addition, we also suggested that the phylogenetic trees based on amino acid sequences were better than those based on nucleotide sequences, according to our results from phylogenetic tree verification.

Besides phylogenetic analysis, natural host is also a key indicator for taxon demarcation criteria. Indeed, the delineation of the natural host is a defining characteristic at subfamily level. For example, *Chordopoxvirinae* subfamily and *Entomopoxvirinae* subfamily infect vertebrates and insects, respectively. However, genus level taxon demarcation based on host range lacks a uniform standard. As new poxvirus isolates continue to be discovered, the range of infected hosts in some genera have continued to widen, and there have been cases where the poxviruses belonging to the same genus can infect different hosts (Table 3). Moreover, since host range expansion is also an evolutionary path for viruses [26], as time goes on, the delineation of host range will become increasingly unsuitable for taxon demarcation. Therefore, we suggested a new type of criteria for genus demarcation: (1) the member of a given genus shares>70% BLAST identity collinearity sequences with the viruses within the same genera, and (2) in the phylogenetic tree based on the 22 Poxvirus core genes, the viruses within same genera can be clustered into the same branch. According to this criteria, *Orthopoxvirus* and *Centapoxvirus* can fall under the same genera. Similarly, *Cervidpoxvirus*, *Suipoxvirus*, *Vespertilionpoxvirus*, *Capripoxvirus*, *Yatapoxvirus*, *Oryzopoxvirus* and *Leporipoxvirus* can be classified into the same genera, and likewise for both *Pteropopoxvirus* and *Mustelpoxvirus* (Fig. 1 and Fig. 2B). This proposal will also be submitted to ICTV for further discussion.

## 5 Data availability statement

The data used to support the findings of this study are available from the corresponding author upon request.

## CRediT authorship contribution statement

**Zehui Yu:** Conceptualization, Formal analysis, Methodology, Funding acquisition, Writing - original draft, Writing - review & editing. **Wenjie Zhang:** Conceptualization, Writing - original draft, Writing - review & editing. **Huancheng Fu:** Methodology, Visualization, Writing - review & editing. **Xiaoxia Zou:** Data curation, Writing - original draft. **Mingde Zhao:** Formal analysis, Writing - review & editing. **Sicheng Liang:** Formal analysis, Writing - review & editing. **Congwei Gu:** Formal analysis. **Qian Yang:** Data curation. **Manli He:** Data curation. **Qihai Xiao:** Formal analysis. **Wudian Xiao:** Data curation. **Lvqin He:** Data curation. **Muhan Lü:** Conceptualization, Funding acquisition, Supervision, Validation Writing - review & editing.

## Declaration of Competing Interest

The authors declare that they have no known competing financial interests or personal relationships that could have appeared to influence the work reported in this paper.

## Figures and Tables

**Supplementary figure 3:**