# Supplementary material for: Genomic analysis of Poxviridae and exploring qualified gene sequences for phylogenetics
Source: Comput Struct Biotechnol J. 2021 Sep 28;19:5479–86. doi: 10.1016/j.csbj.2021.09.031 (PMC8515299; doi:10.1016/j.csbj.2021.09.031)
Supplement: Supplementary data 1 [file mmc1.docx]

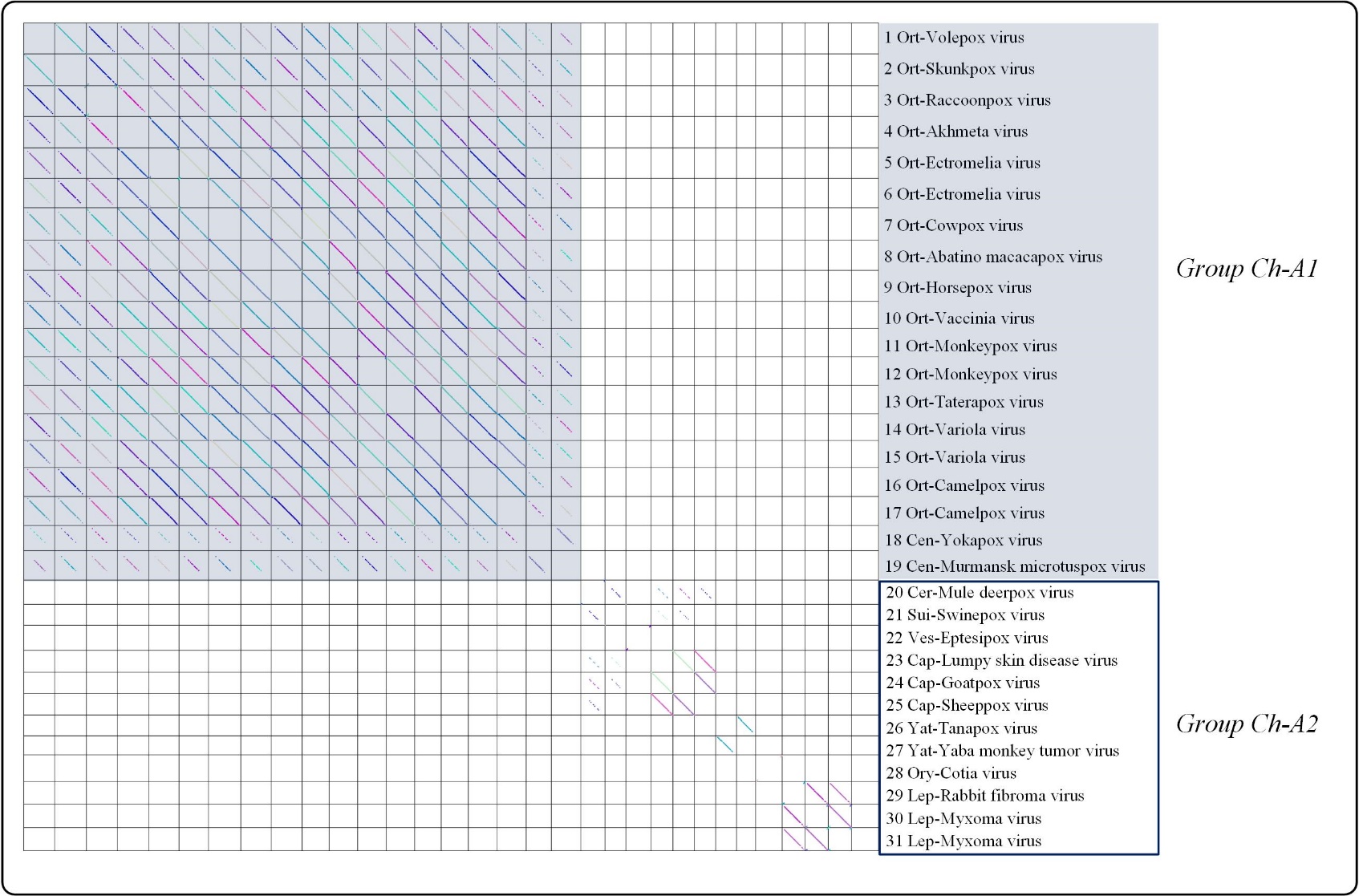


**Supplementary Figure 1** Synteny analysis of Group *Ch-A* (identity threshold set as 85%). The first three letters are abbreviated from the genus name (e.g. Ort-Volepox virus, Ort means *Orthopoxvirus*). Each corresponding block represents the collinearity comparison of two viruses. The colours of lines in a block are to distinguish between different collinear regions. The colours between blocks are irrelevant.


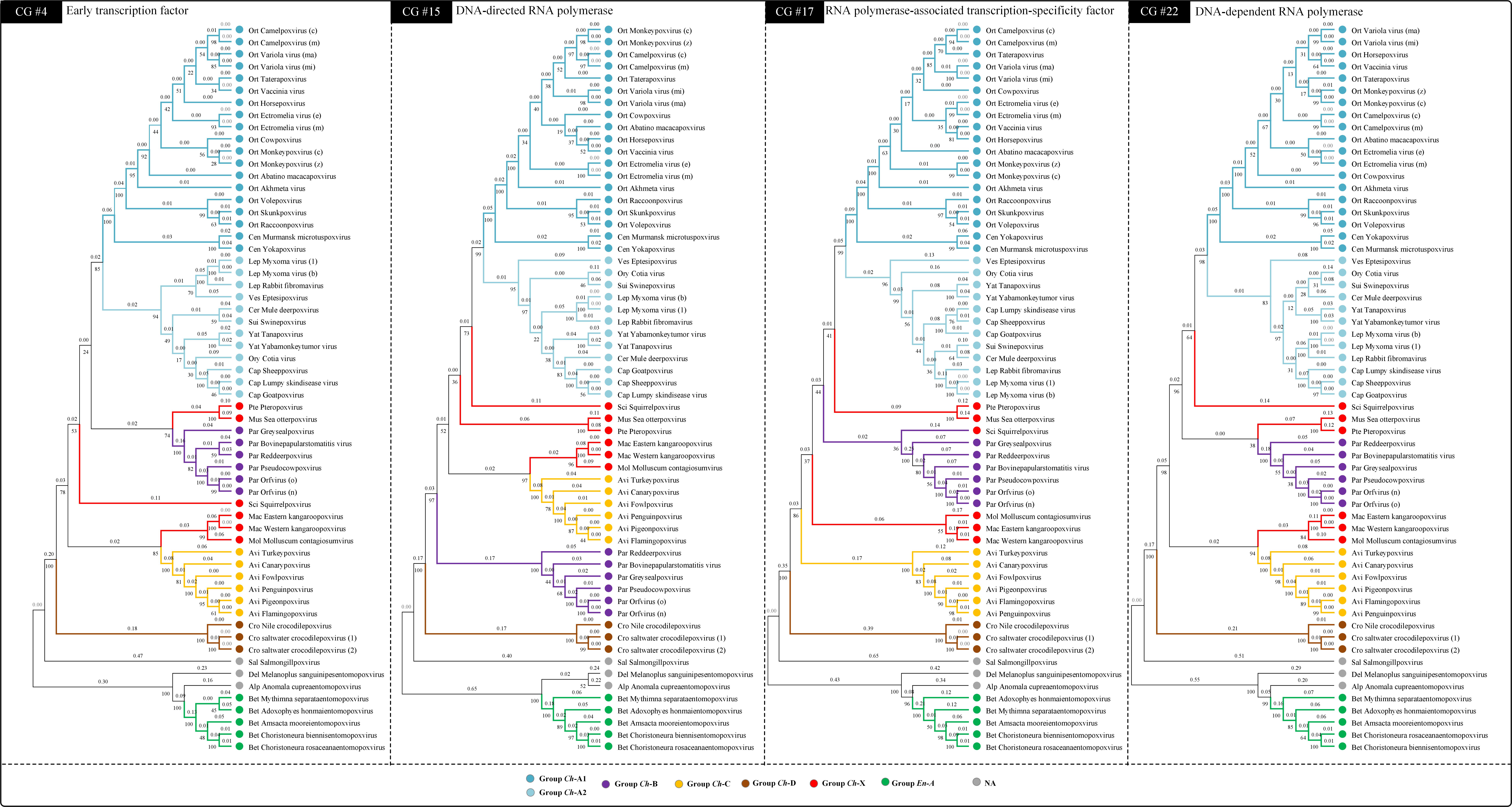


**Supplementary Figure 2.** The neighbor joining phylogenetic trees based on single amino acid sequence. The numbers on the branches represent branch lengths/genetic distances and numbers below the branch points represent bootstrap values. The color of the branch endpoints represents the classification results based on synteny analysis.


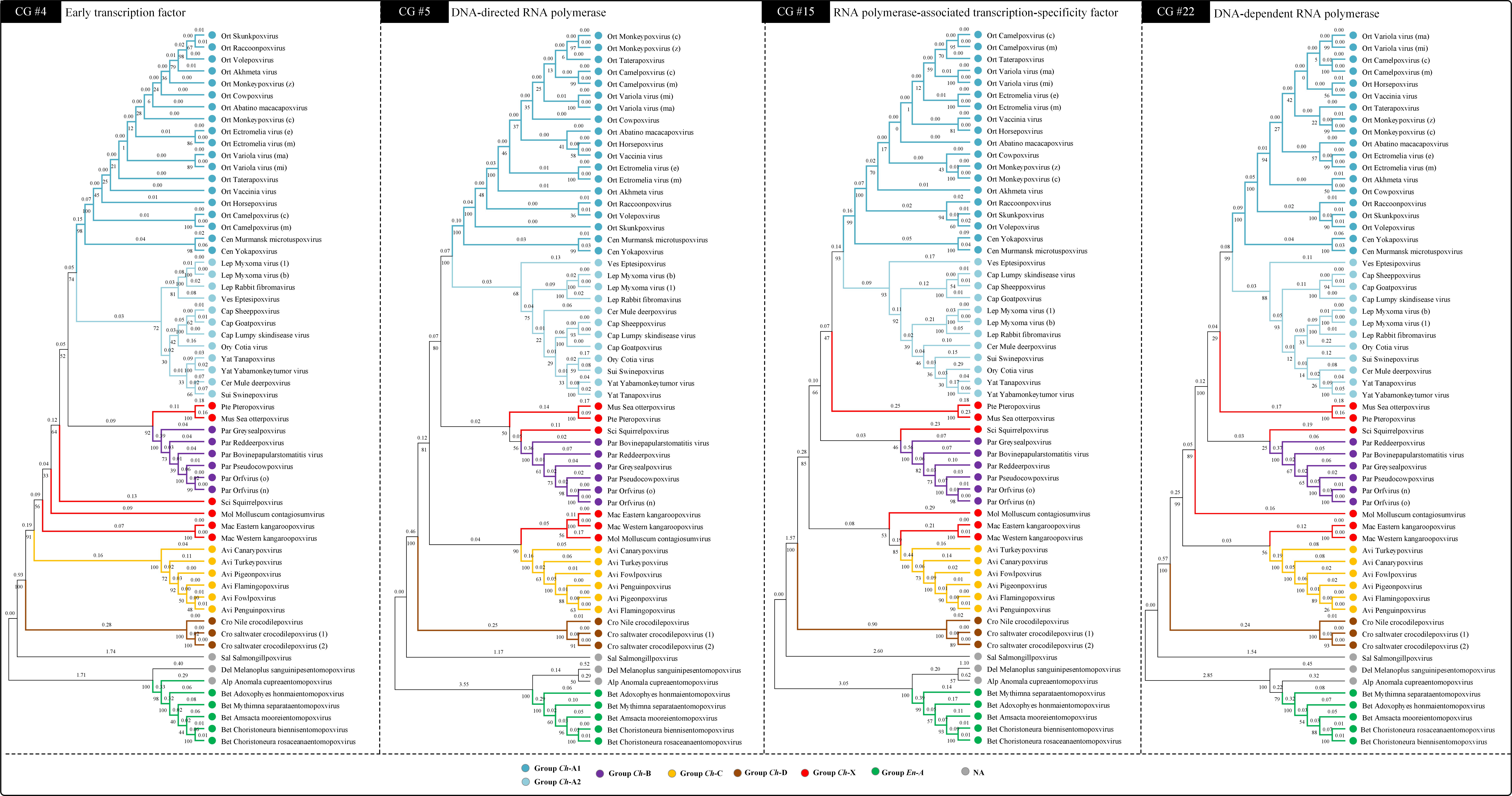


**Supplementary Figure 3.** The maximum likelihood phylogenetic trees based on single amino acid sequence. The numbers on the branches represent branch lengths/genetic distances and numbers below the branch points represent bootstrap values. The color of the branch endpoints represents the classification results based on synteny analysis.
