# Supplementary material for: Genomic analysis of Poxviridae and exploring qualified gene sequences for phylogenetics
Source: Comput Struct Biotechnol J. 2021 Sep 28;19:5479–86. doi: 10.1016/j.csbj.2021.09.031 (PMC8515299; doi:10.1016/j.csbj.2021.09.031)

Supplementary file 1: The NJ-Tree based on single nucleotide sequence of core genes. The numbers on the branches represent branch lengths/genetic distances and numbers below the branch points represent bootstrap values. The color of the branch endpoints represents the classification results based on synteny analysis.

Legend

- **Group *Ch*-A1**
- **Group *Ch*-A2**
- **Group *Ch*-B**
- **Group *Ch*-C**
- **Group *Ch*-D**
- **Group *Ch*-X**
- **Group *En-A***
- **NA**

The NJ-Tree based on CG #1 nucleotide sequence

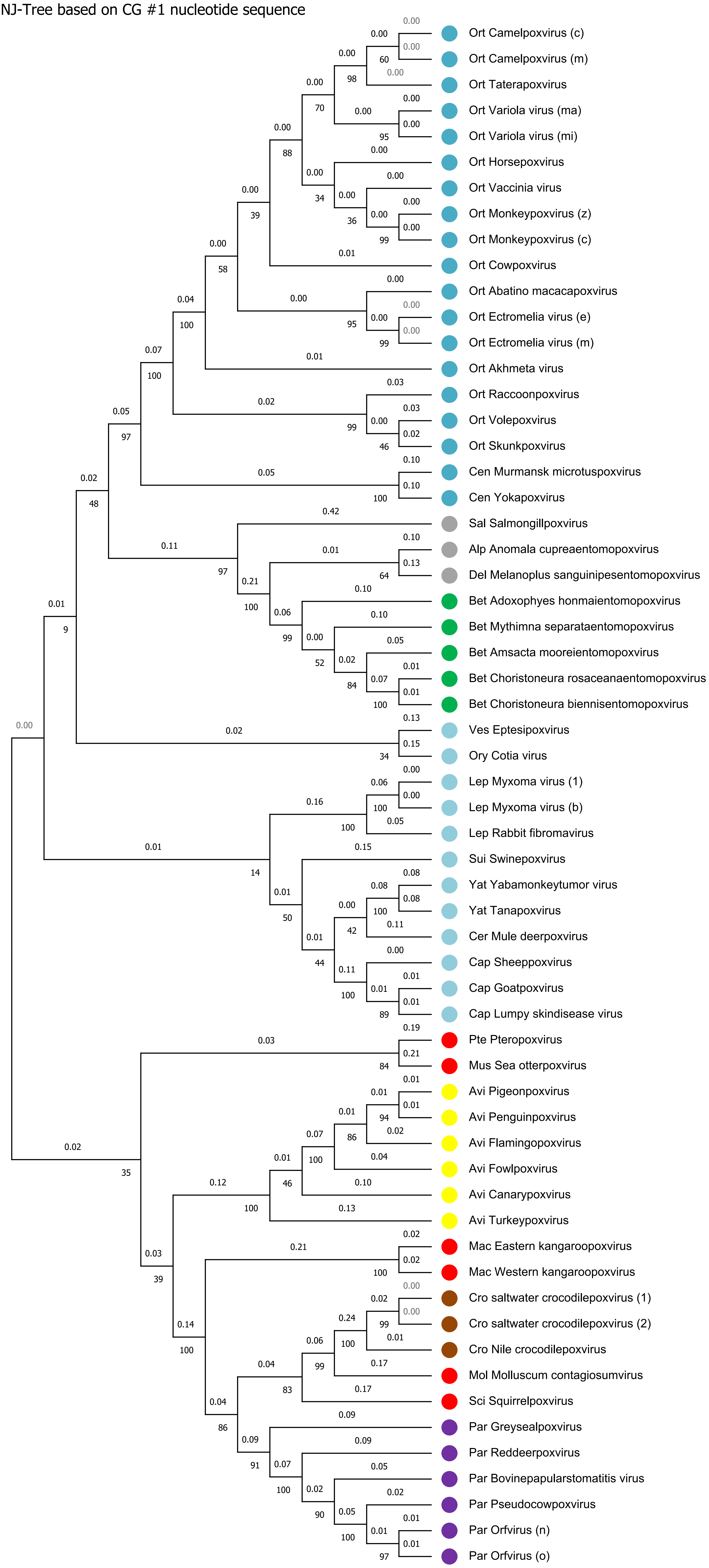

The NJ-Tree based on CG #2 nucleotide sequence

Experience substantial substitution saturation

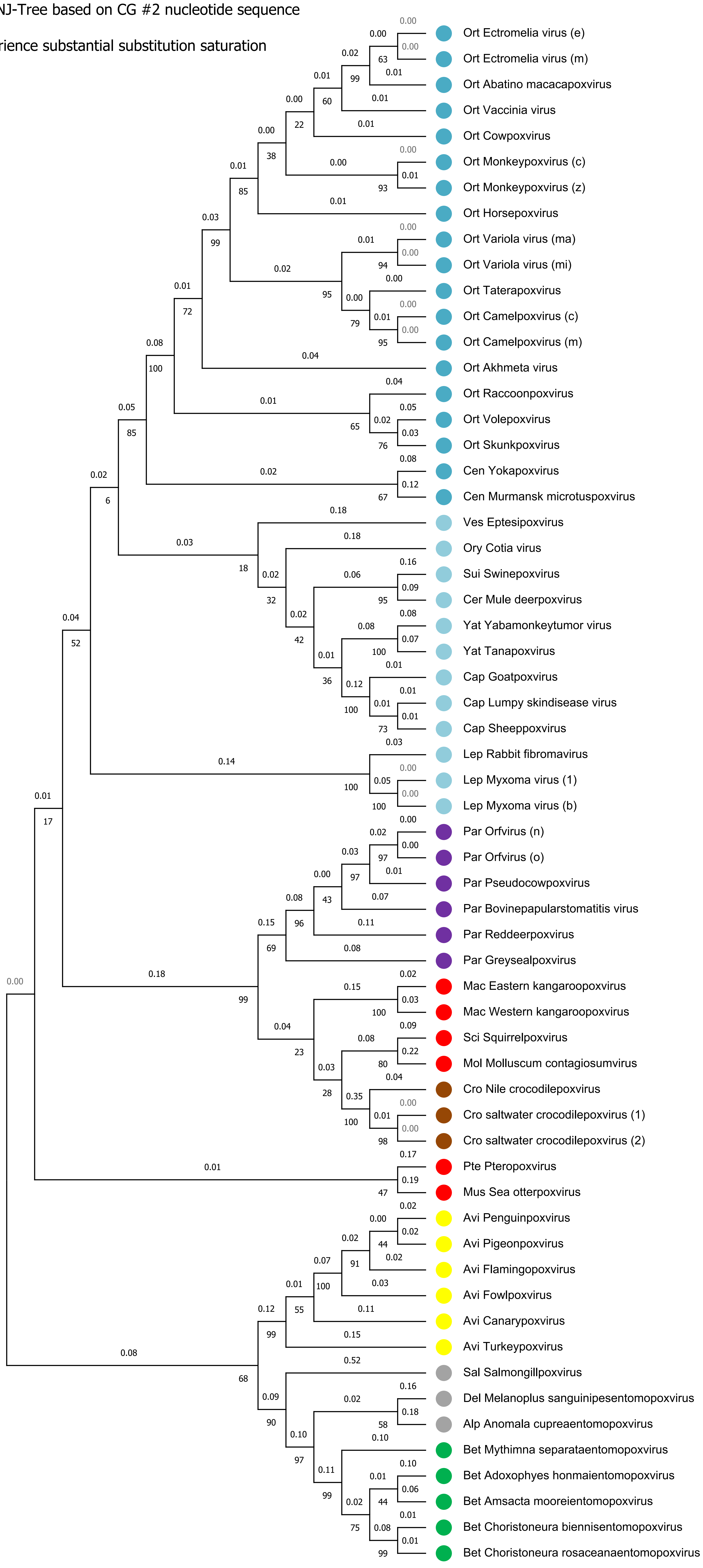

The NJ-Tree based on CG #3 nucleotide sequence

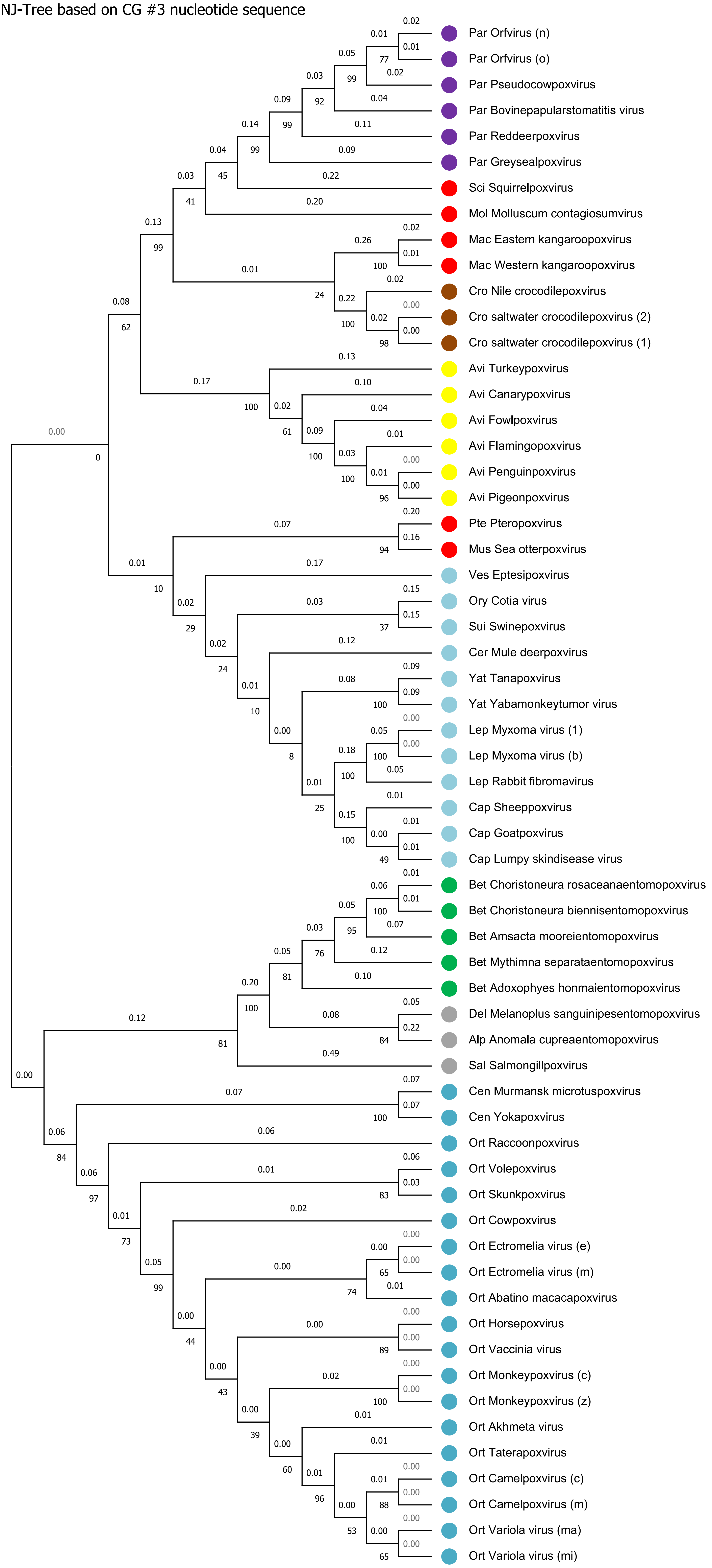

The NJ-Tree based on CG #4 nucleotide sequence

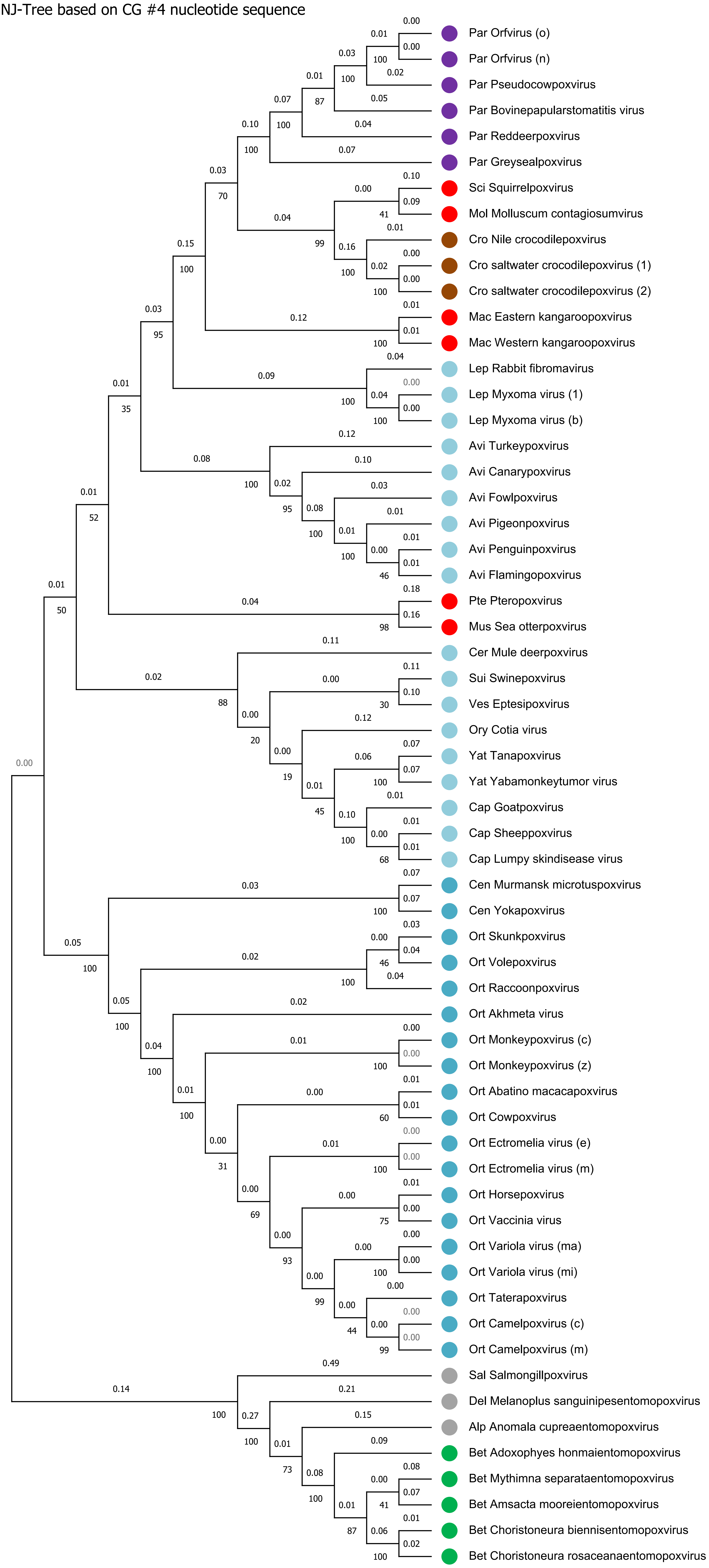

The NJ-Tree based on CG #5 nucleotide sequence

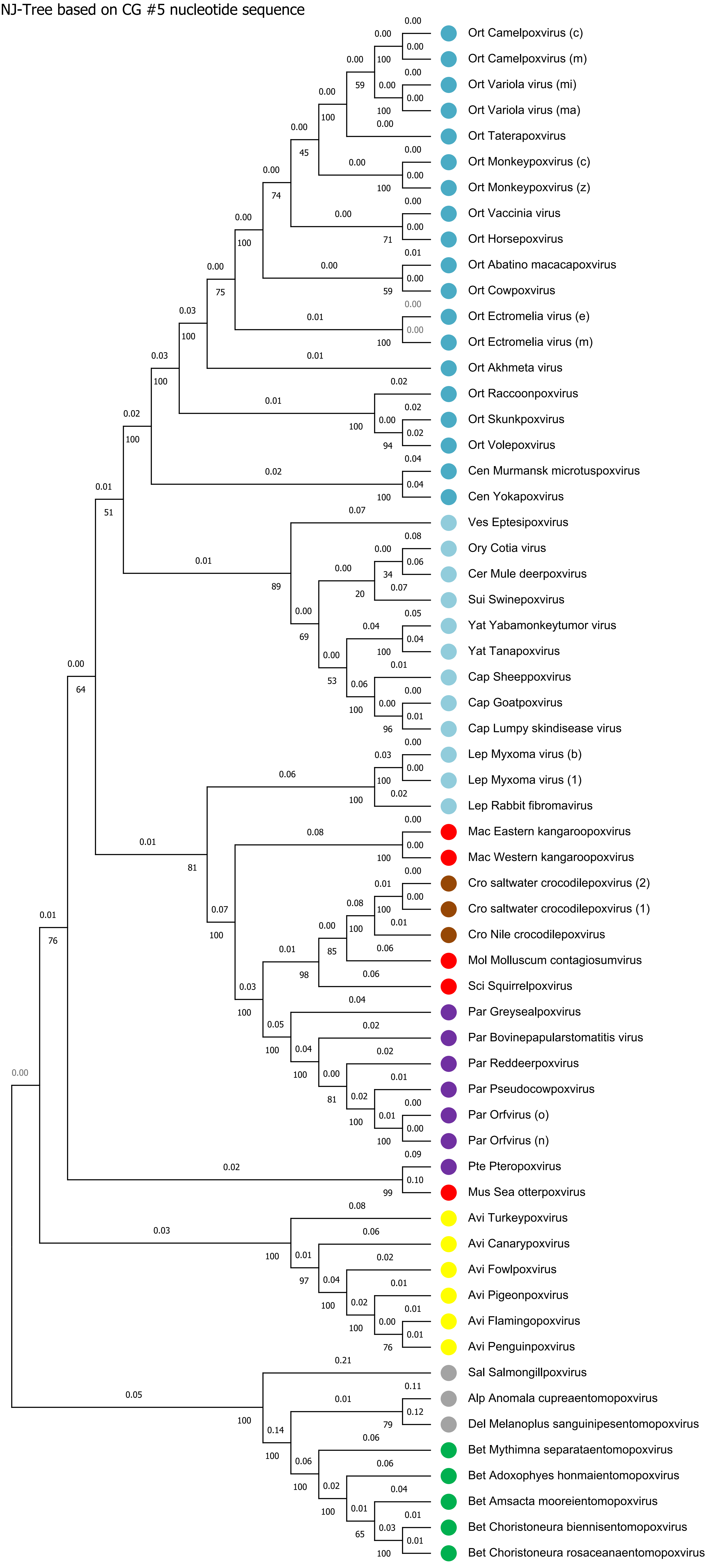

The NJ-Tree based on CG #6 nucleotide sequence

Experience substantial substitution saturation

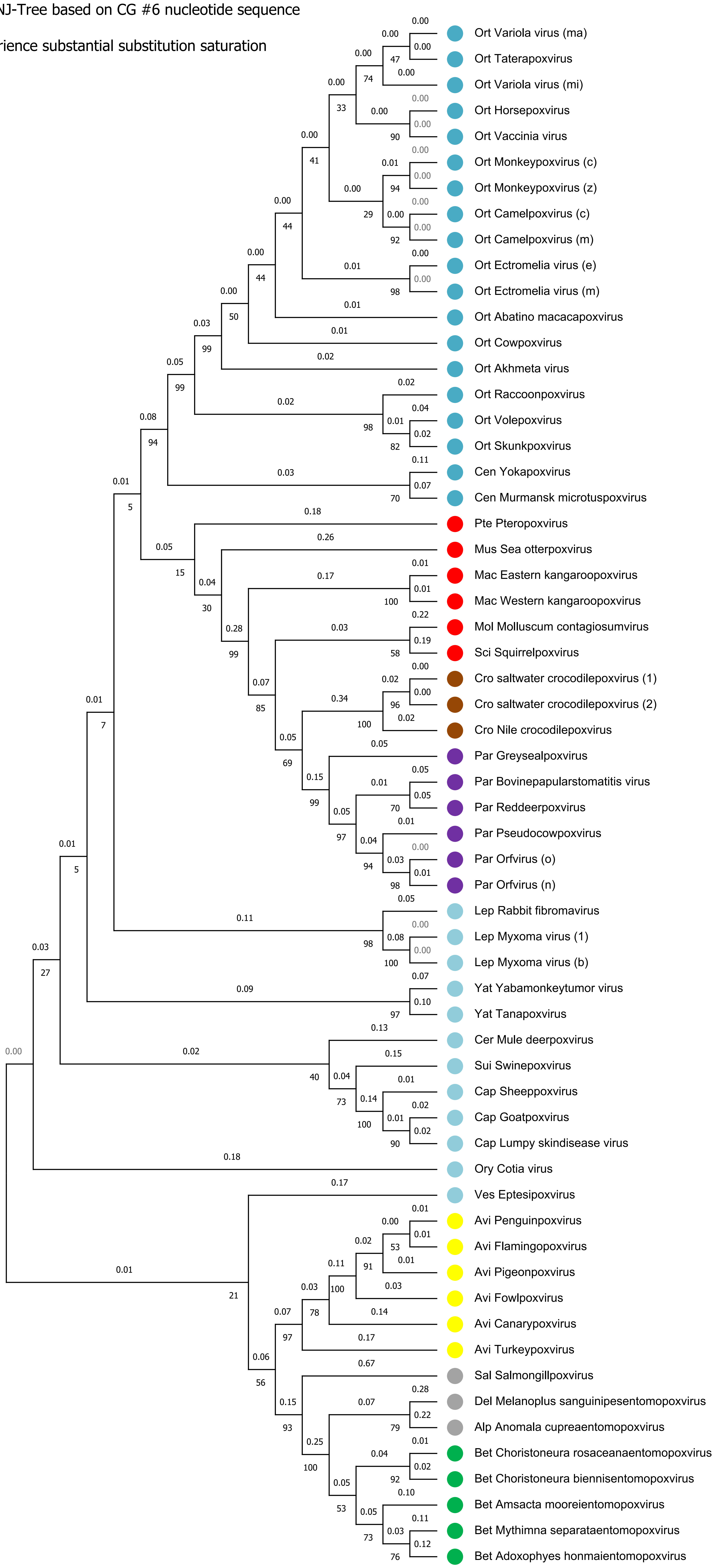

The NJ-Tree based on CG #7 nucleotide sequence

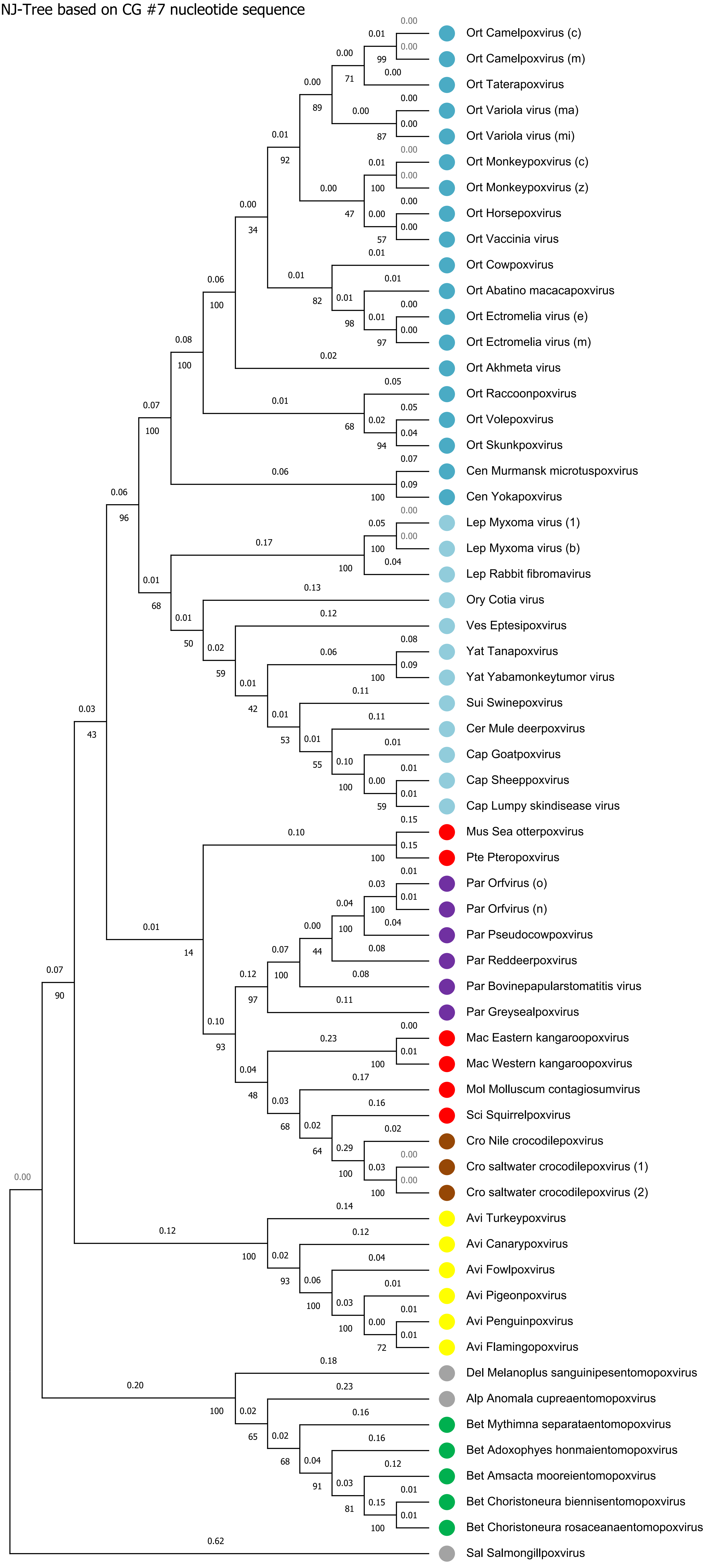

The NJ-Tree based on CG #8 nucleotide sequence

Experience substantial substitution saturation

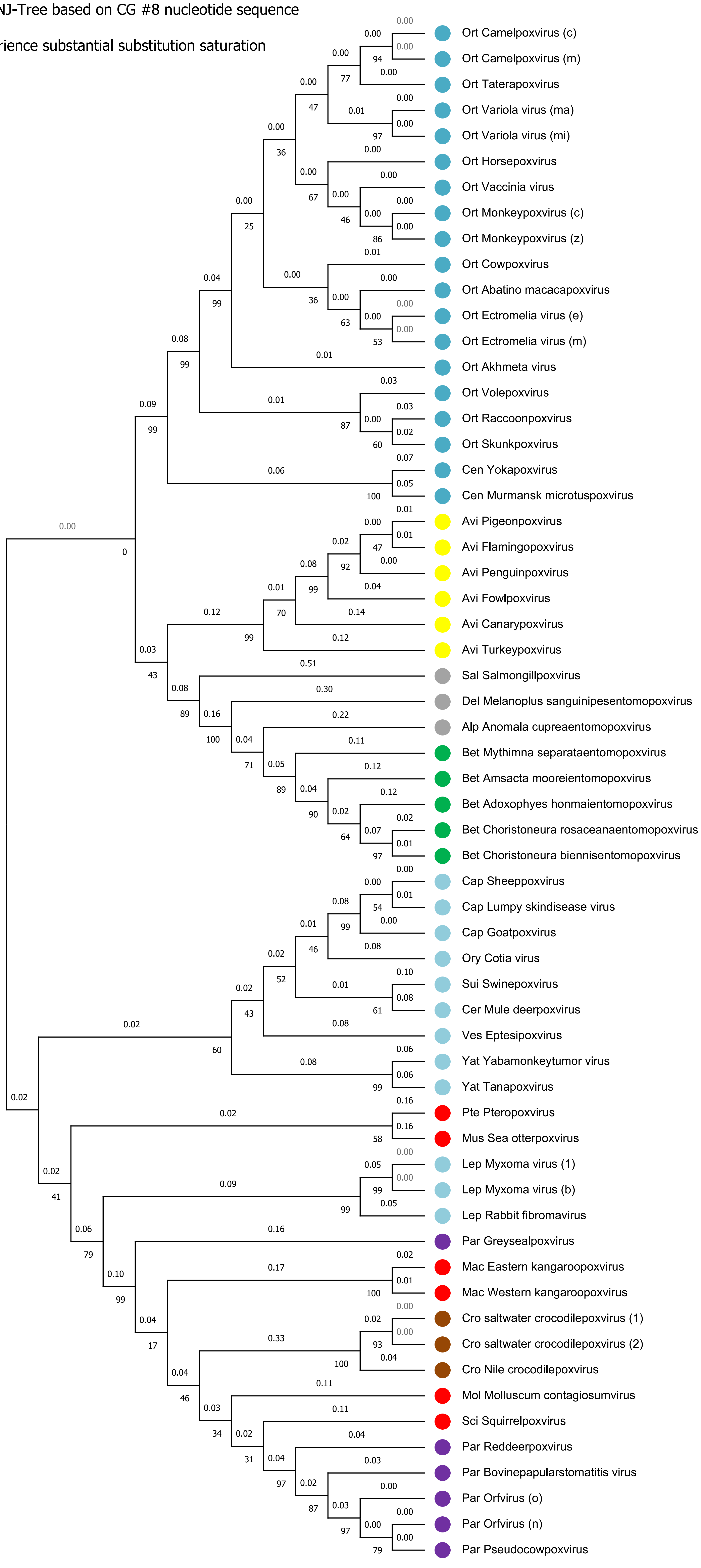

The NJ-Tree based on CG #9 nucleotide sequence

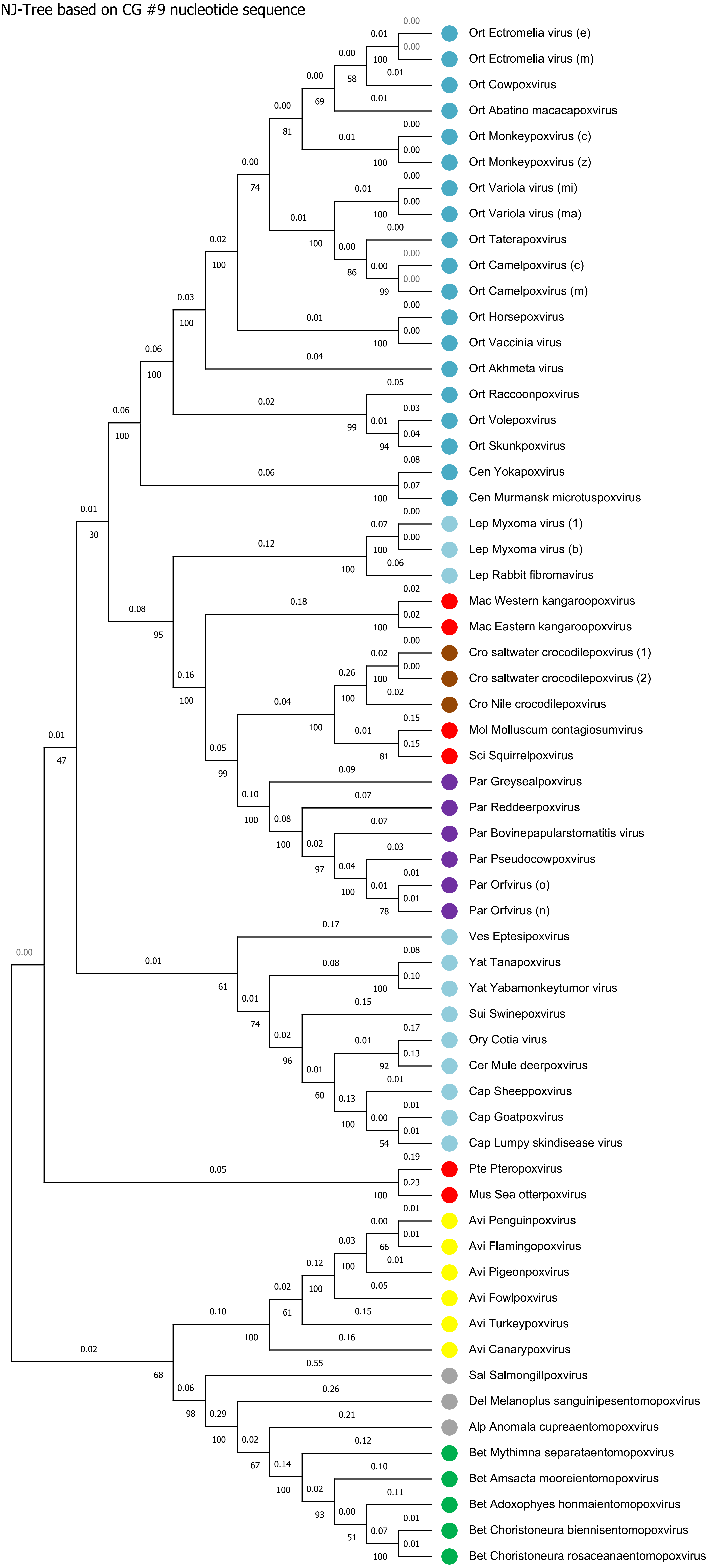

The NJ-Tree based on CG #10 nucleotide sequence

Experience substantial substitution saturation

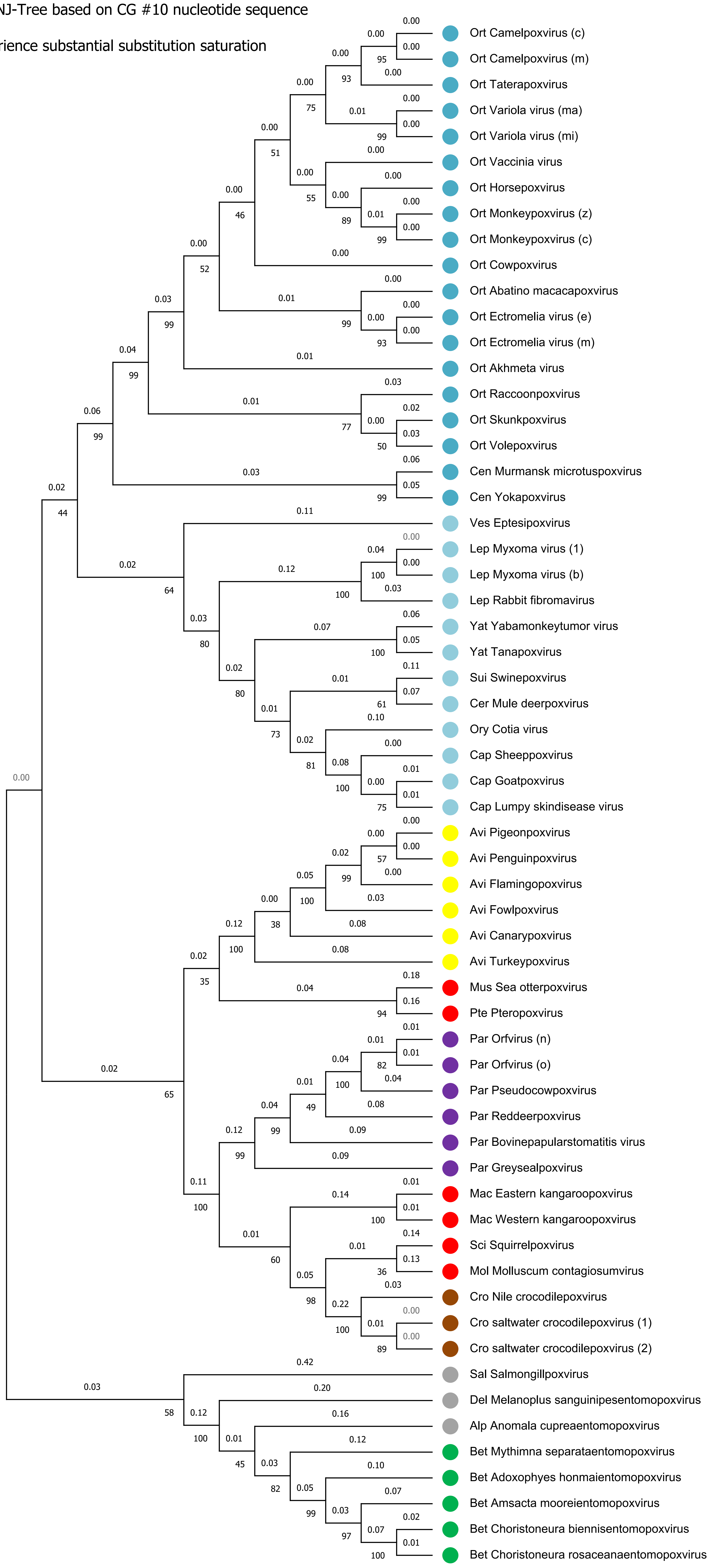

The NJ-Tree based on CG #11 nucleotide sequence

Experience substantial substitution saturation

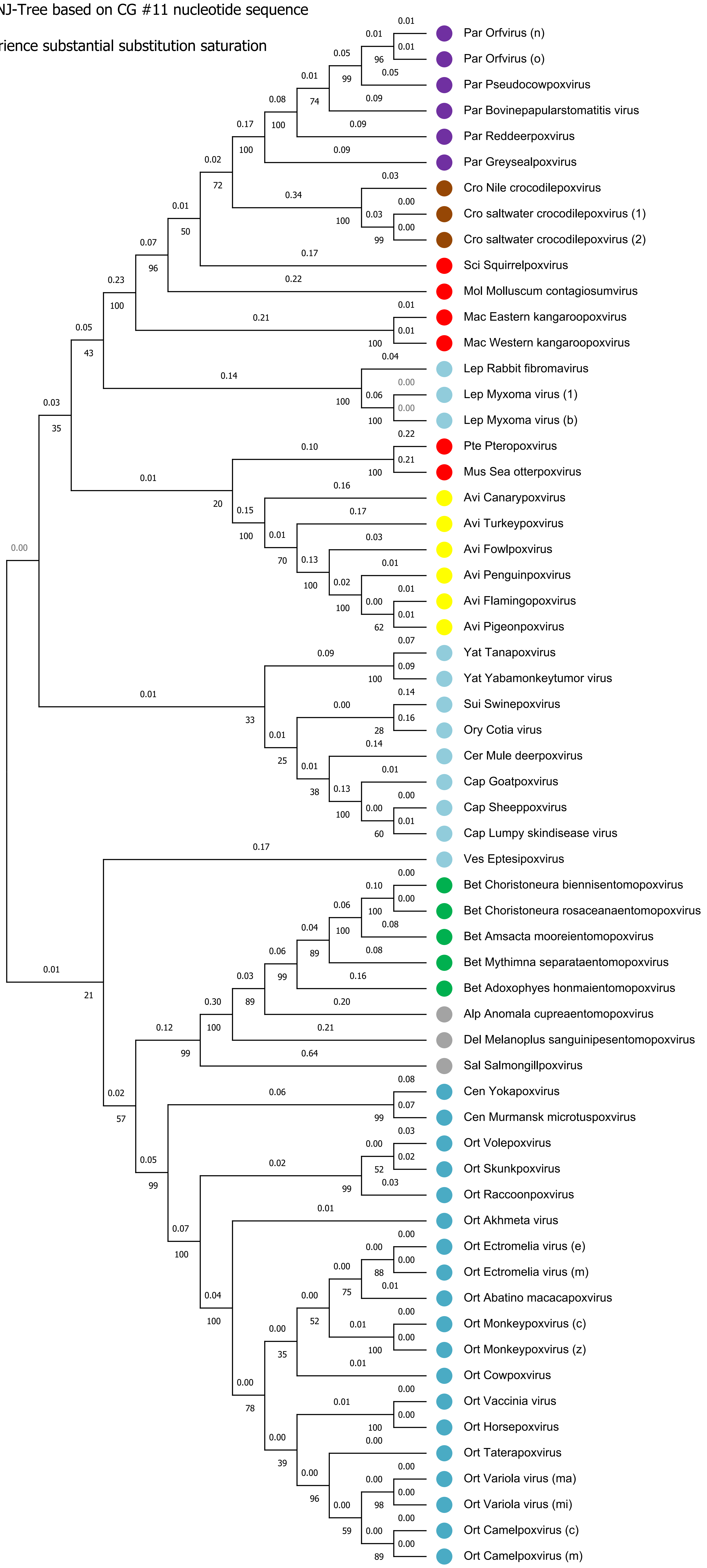

The NJ-Tree based on CG #12 nucleotide sequence

Experience substantial substitution saturation

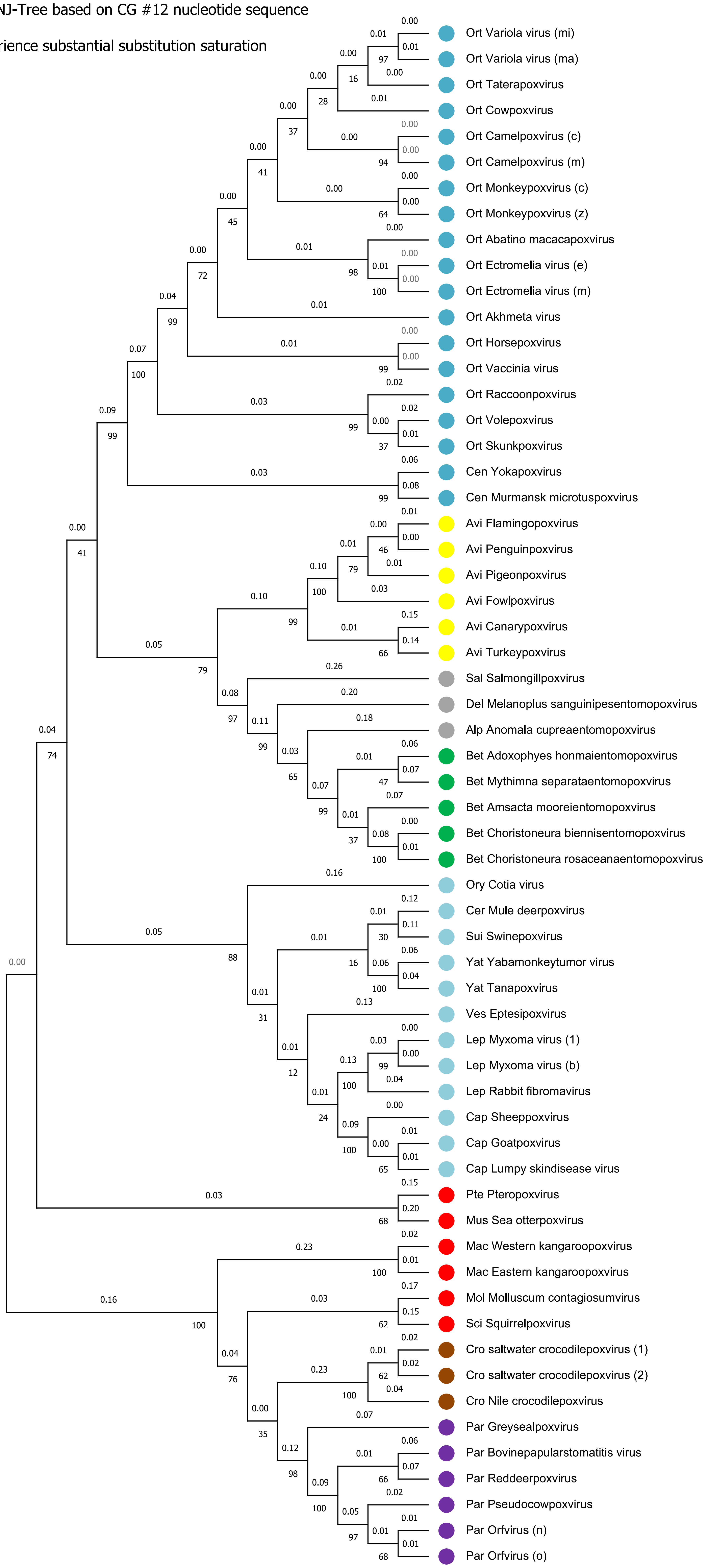

The NJ-Tree based on CG #13 nucleotide sequence

Experience substantial substitution saturation

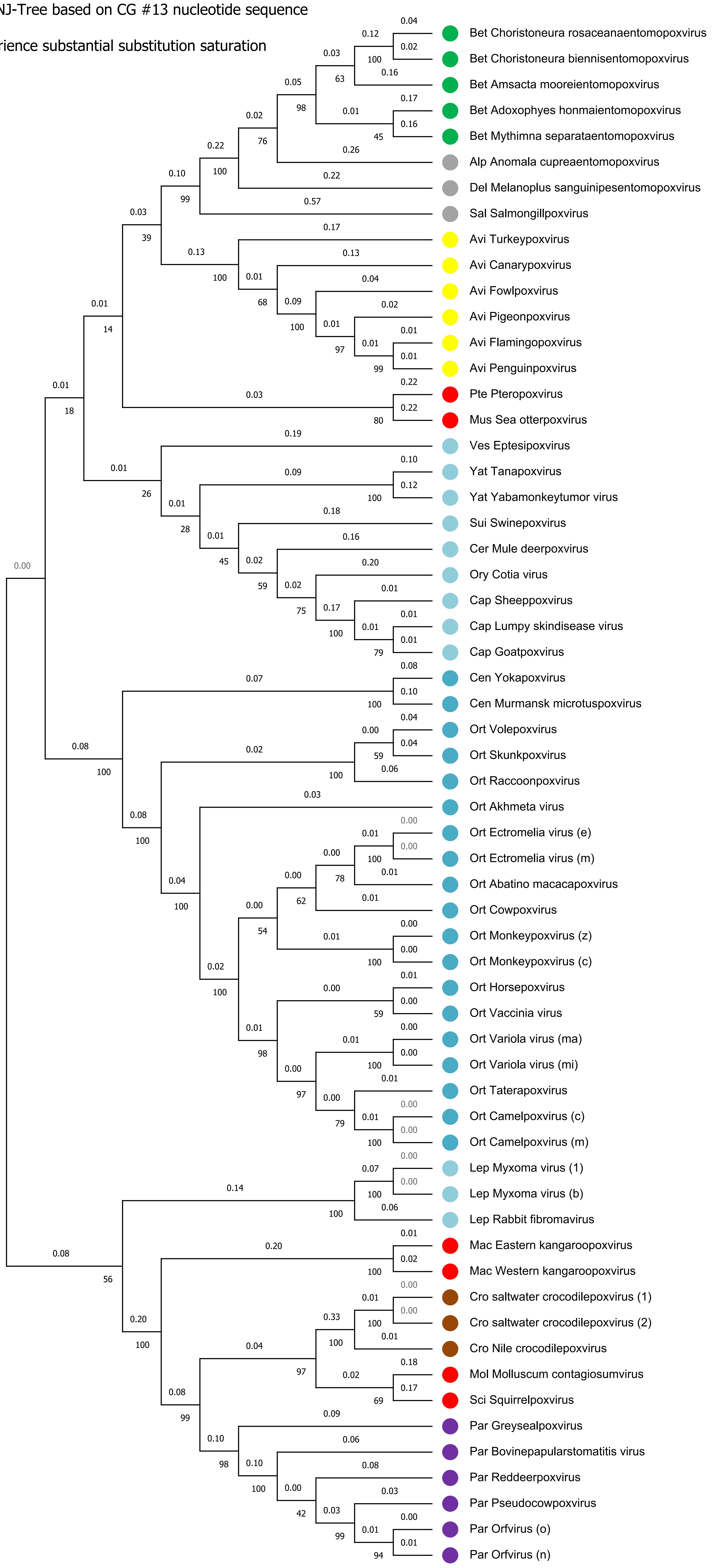

The NJ-Tree based on CG #14 nucleotide sequence

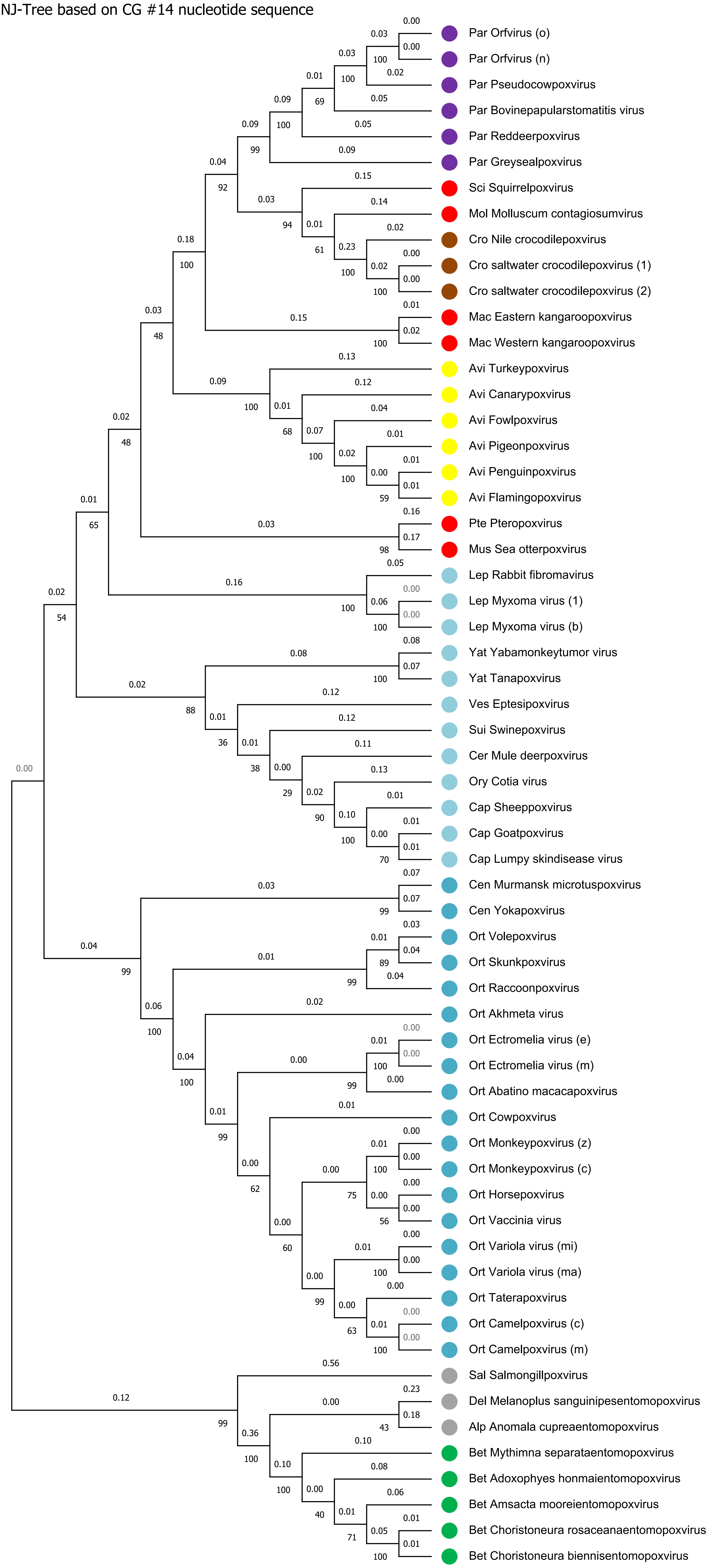

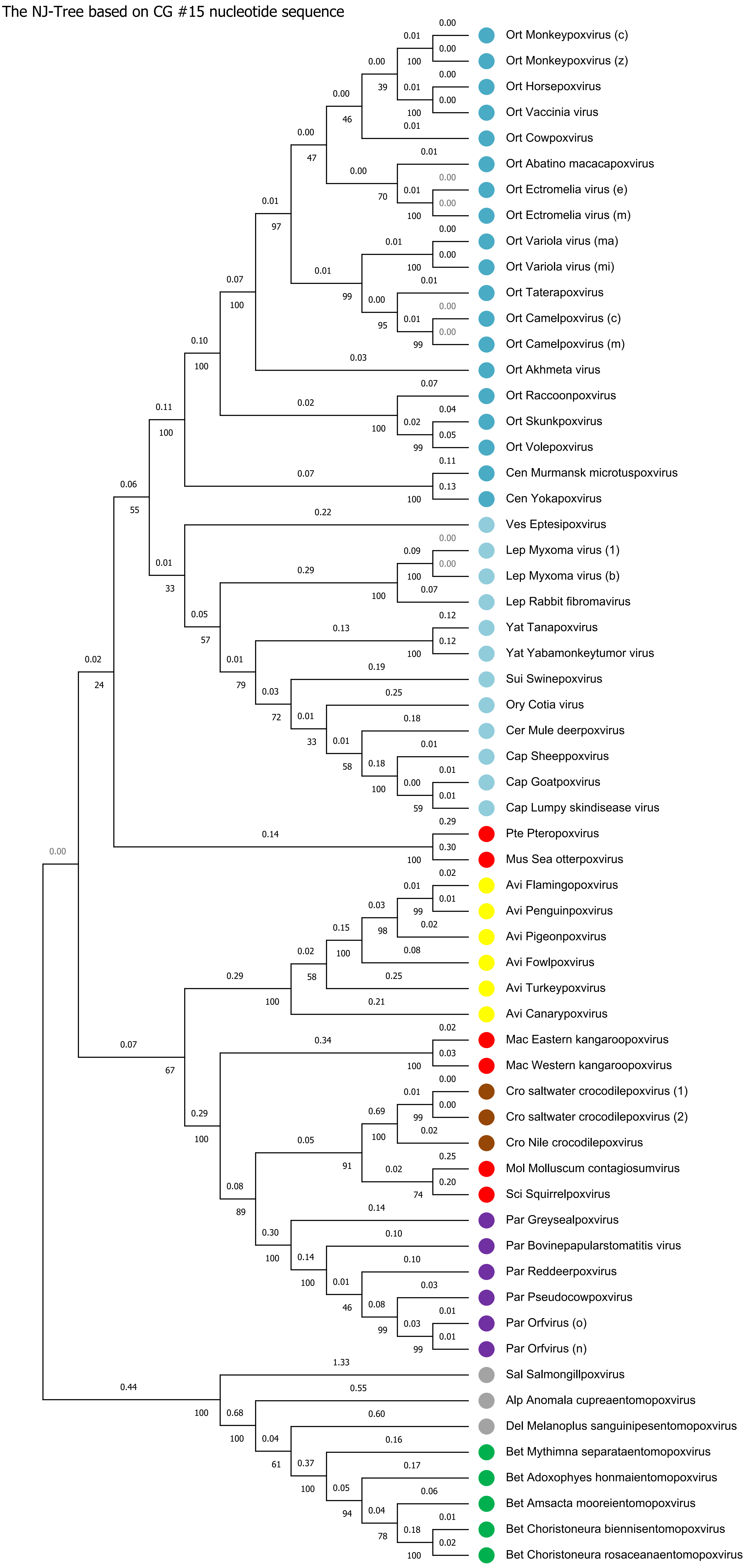
The NJ-Tree based on CG #15 nucleotide sequence
Ort Monkeypoxvirus (c)
Ort Monkeypoxvirus (z)
Ort Horsepoxvirus
Ort Vaccinia virus
Ort Cowpoxvirus
Ort Abatino macacapoxvirus
Ort Ectromelia virus (e)
Ort Ectromelia virus (m)
Ort Variola virus (ma)
Ort Variola virus (mi)
Ort Taterapoxvirus
Ort Camelpoxvirus (c)
Ort Camelpoxvirus (m)
Ort Akhmeta virus
Ort Raccoonpoxvirus
Ort Skunkpoxvirus
Ort Volepoxvirus
Cen Murmansk microtuspoxvirus
Cen Yokapoxvirus
Ves Eptesipoxvirus
Lep Myxoma virus (1)
Lep Myxoma virus (b)
Lep Rabbit fibromavirus
Yat Tanapoxvirus
Yat Yabamonkeytumor virus
Sui Swinepoxvirus
Ory Cotia virus
Cer Mule deerpoxvirus
Cap Sheeppoxvirus
Cap Goatpoxvirus
Cap Lumpy skindisease virus
Pte Pteropoxvirus
Mus Sea otterpoxvirus
Avi Flamingopoxvirus
Avi Penguinpoxvirus
Avi Pigeonpoxvirus
Avi Fowlpoxvirus
Avi Turkeypoxvirus
Avi Canarypoxvirus
Mac Eastern kangaroopoxvirus
Mac Western kangaroopoxvirus
Cro saltwater crocodilepoxvirus (1)
Cro saltwater crocodilepoxvirus (2)
Cro Nile crocodilepoxvirus
Mol Molluscum contagiosumvirus
Sci Squirrelpoxvirus
Par Greysealpoxvirus
Par Bovinepapularstomatitis virus
Par Reddeerpoxvirus
Par Pseudocowpoxvirus
Par Orfvirus (o)
Par Orfvirus (n)
Sal Salmongillpoxvirus
Alp Anomala cupreaentomopoxvirus
Del Melanoplus sanguinipesentomopoxvirus
Bet Mythimna separataentomopoxvirus
Bet Adoxophyes honmaientomopoxvirus
Bet Amsacta mooreientomopoxvirus
Bet Choristoneura biennisentomopoxvirus
Bet Choristoneura rosaceanaentomopoxvirus

The NJ-Tree based on CG #16 nucleotide sequence

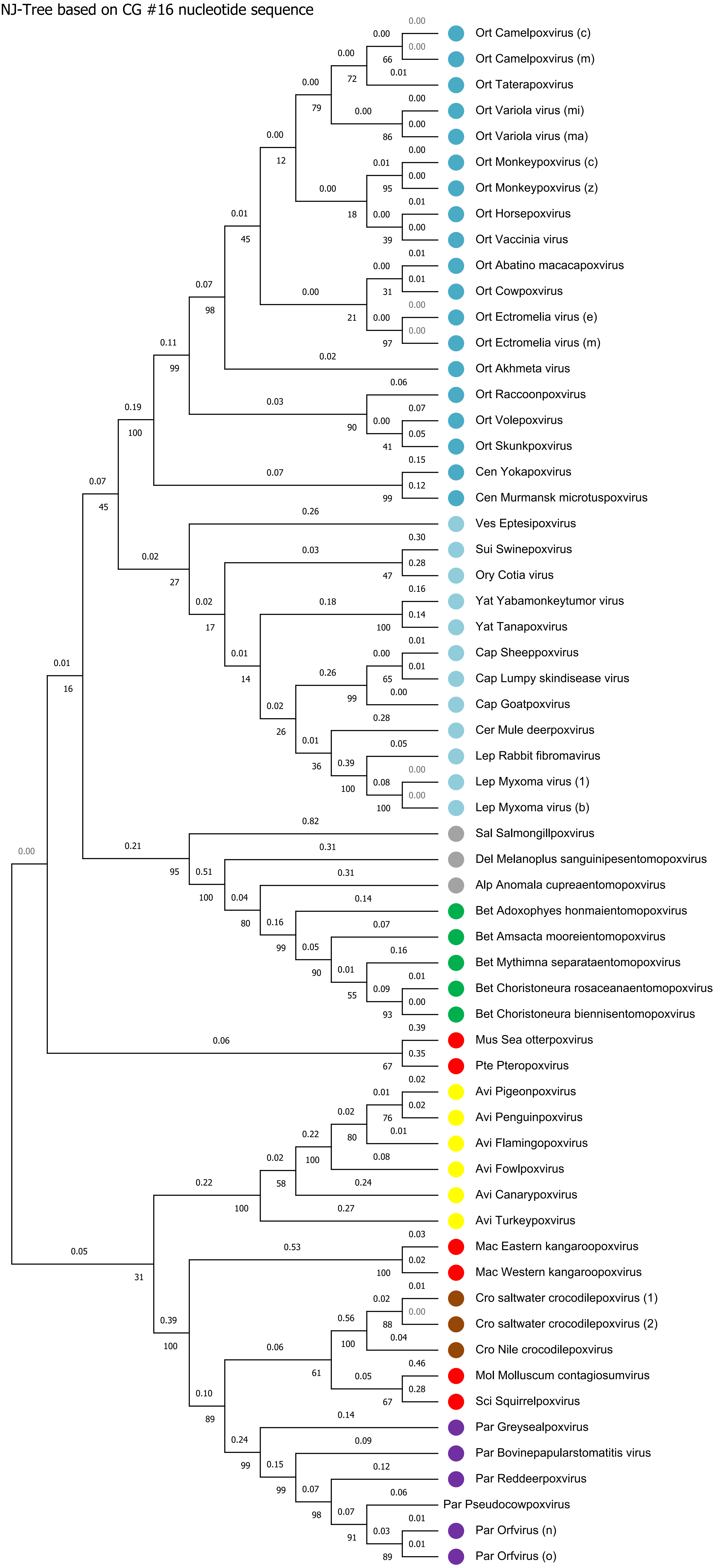

The NJ-Tree based on CG #17 nucleotide sequence

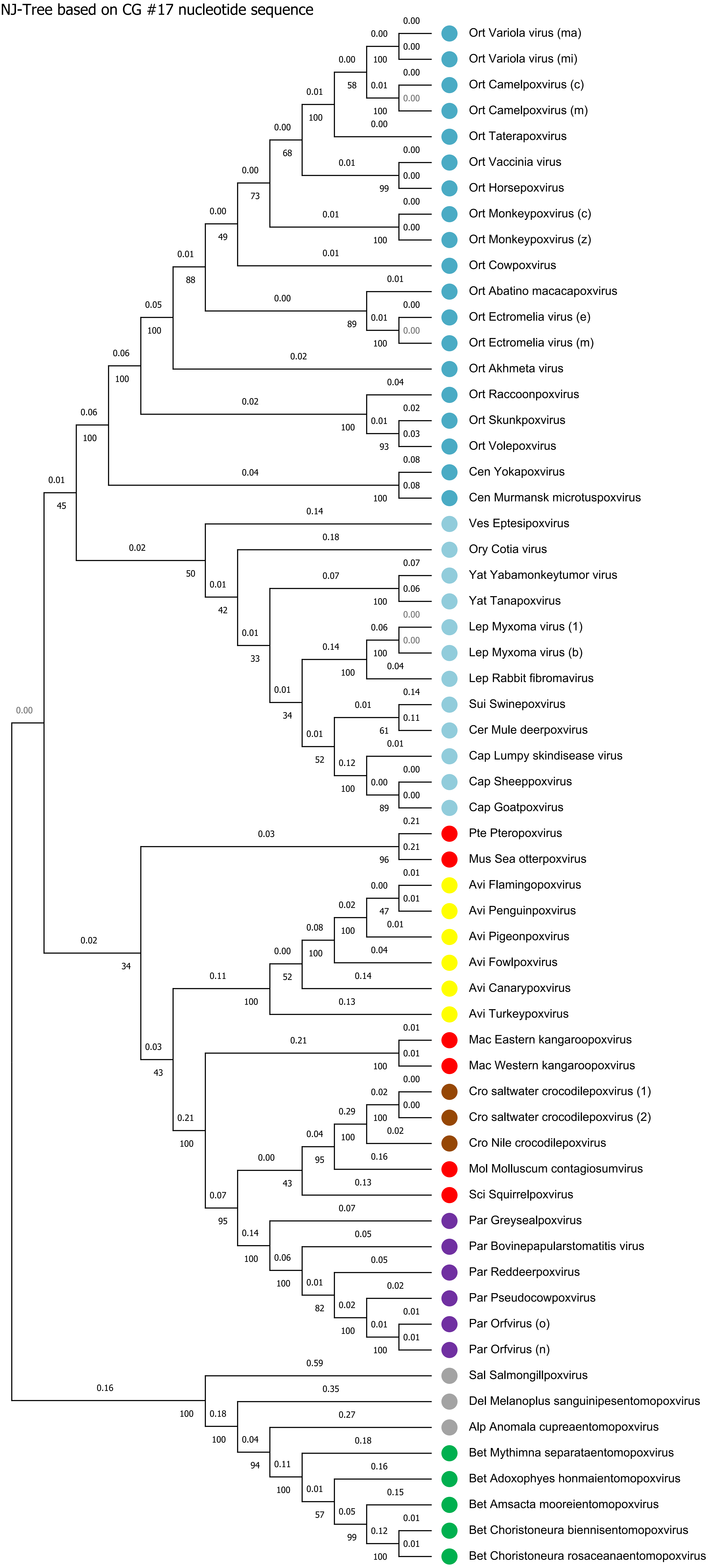

The NJ-Tree based on CG #18 nucleotide sequence

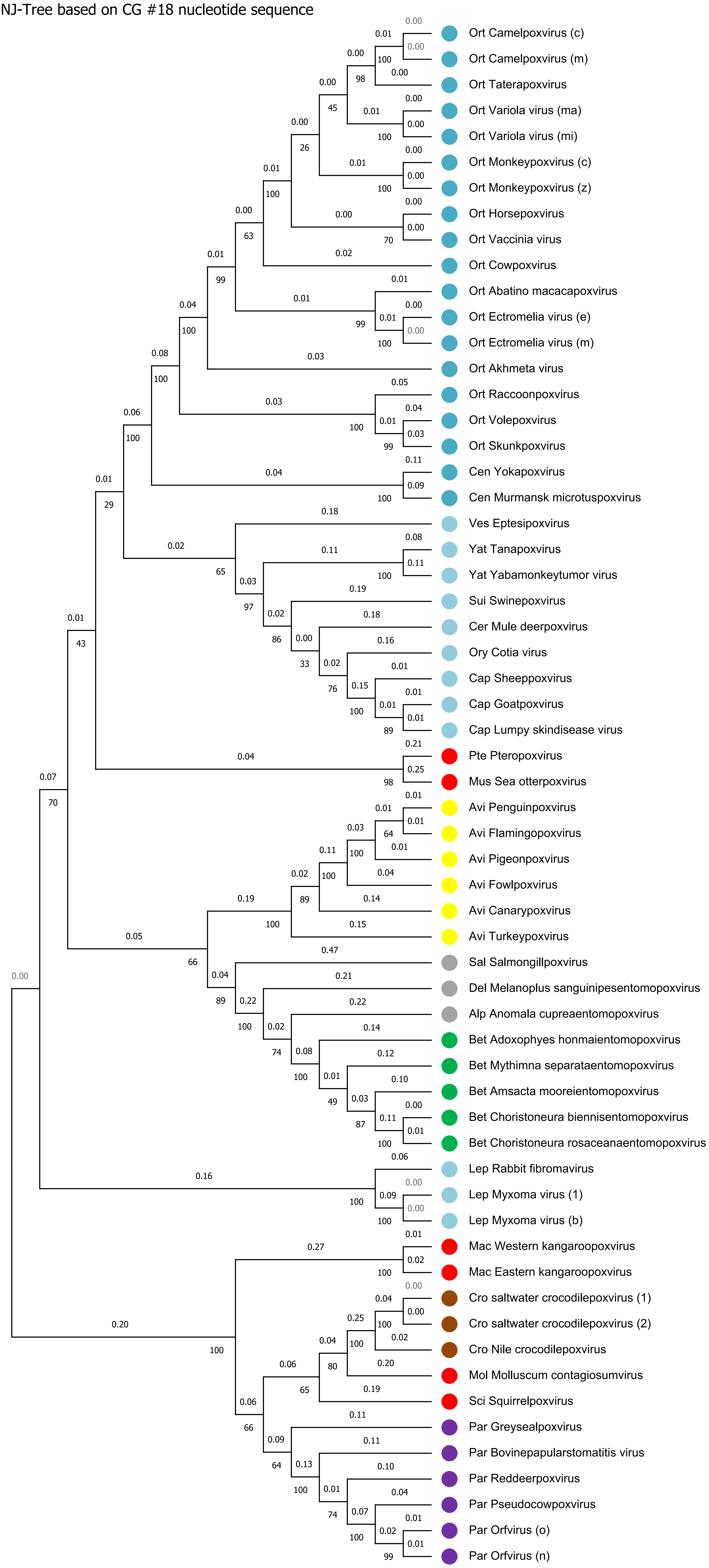

The NJ-Tree based on CG #19 nucleotide sequence

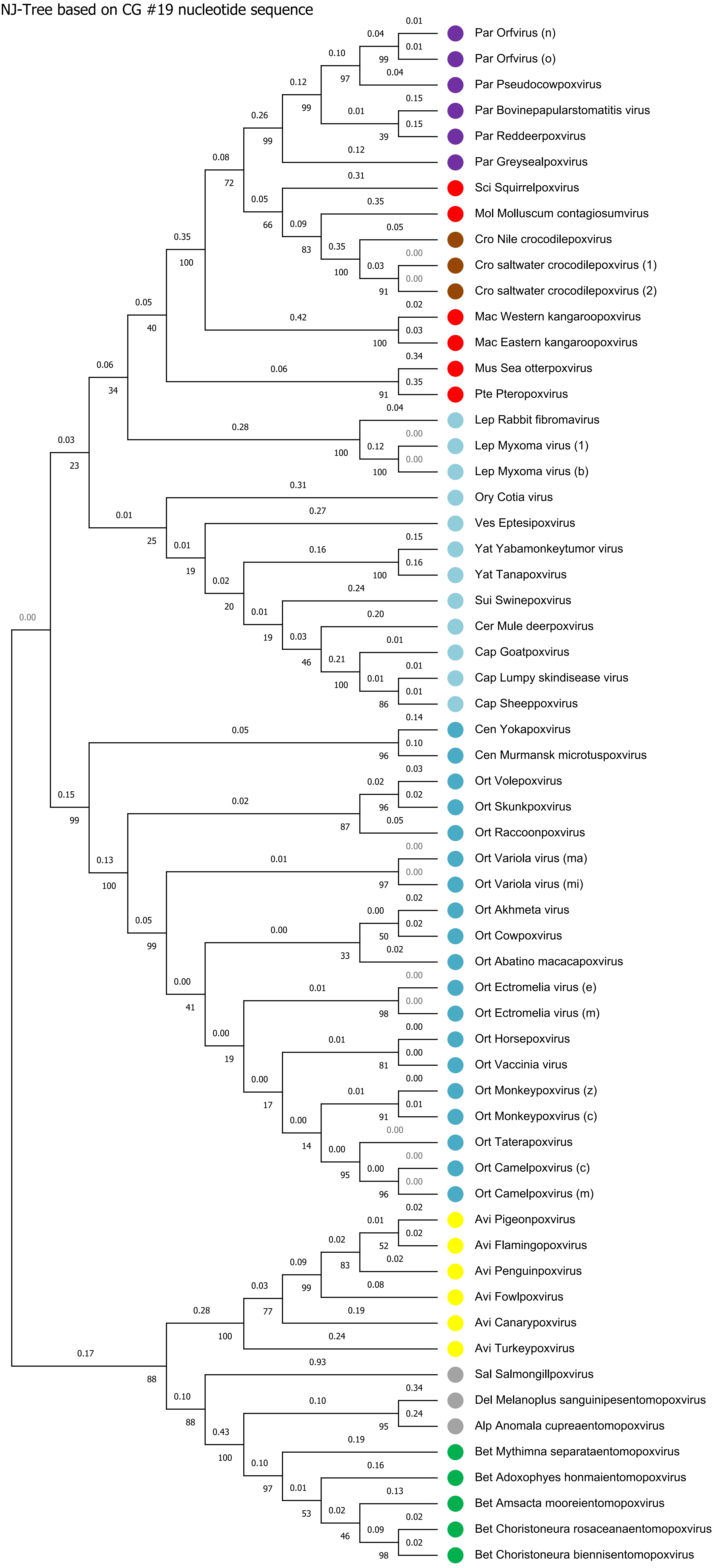

The NJ-Tree based on CG #20 nucleotide sequence

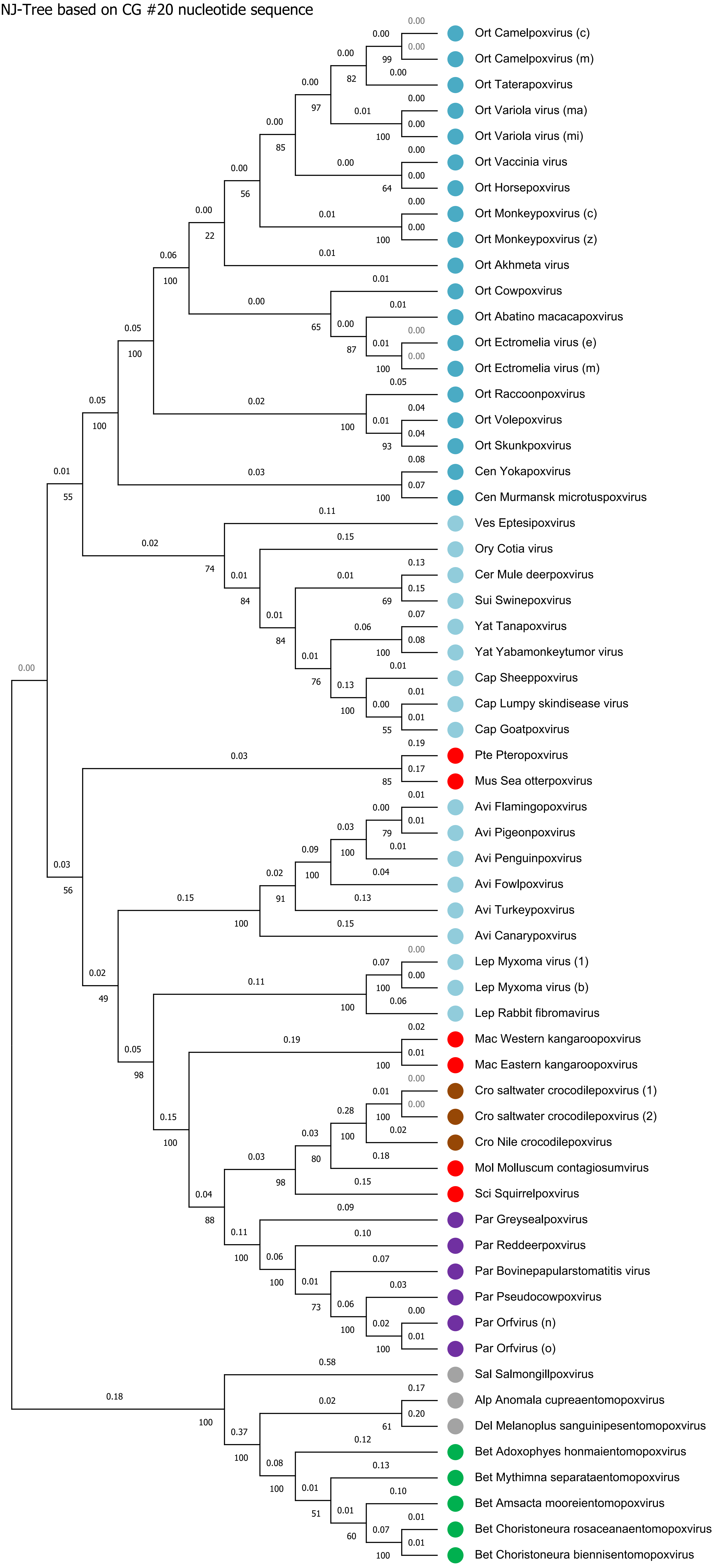

The NJ-Tree based on CG #21 nucleotide sequence

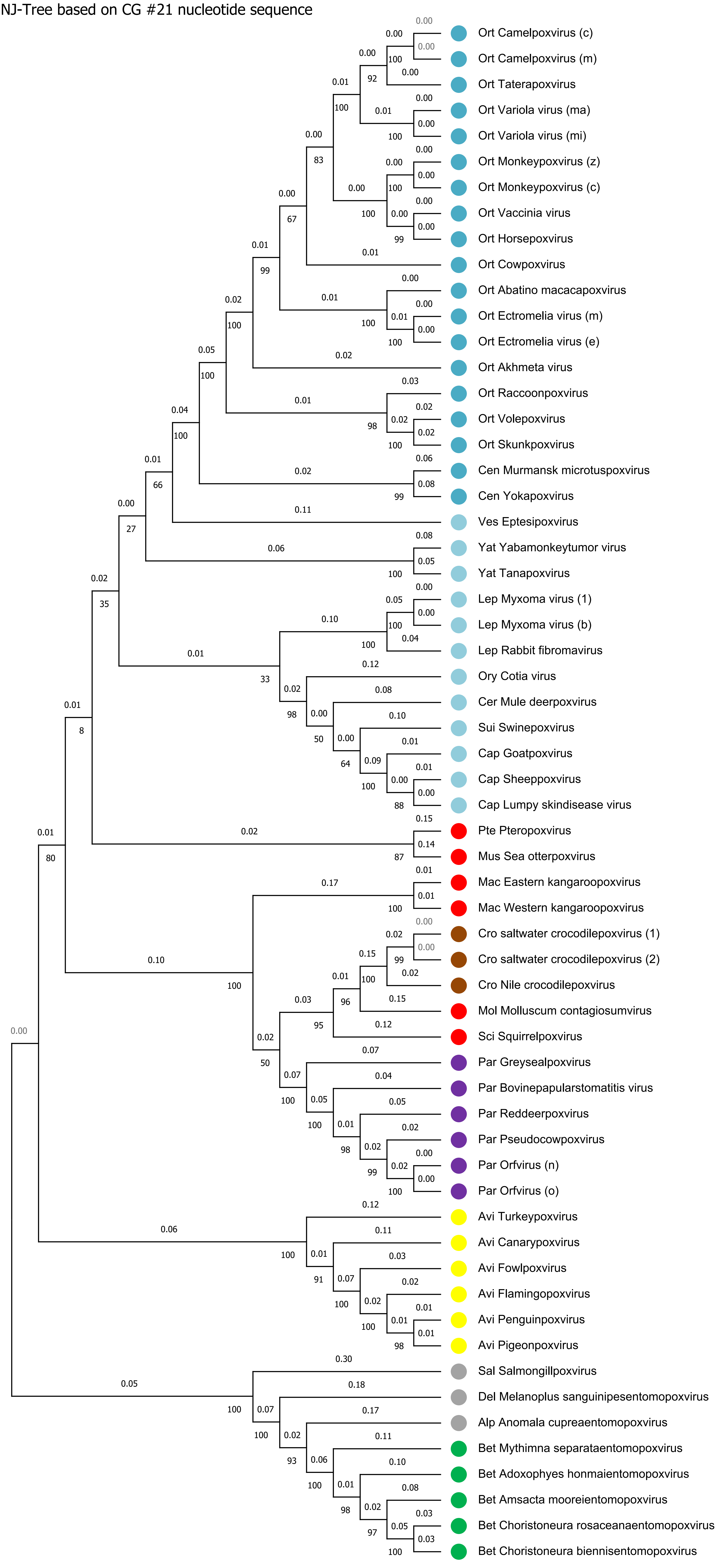

The NJ-Tree based on CG #22 nucleotide sequence

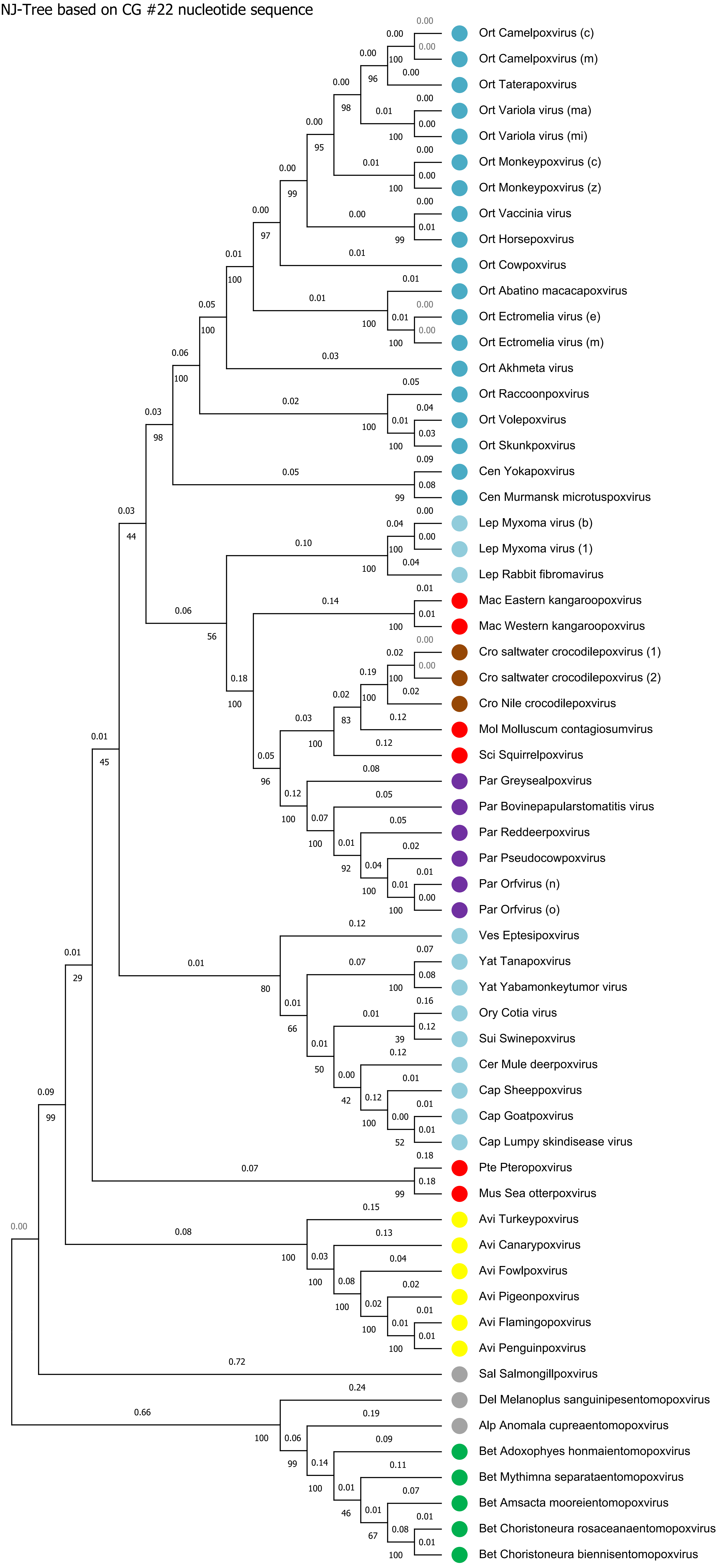

Supplement: Supplementary data 5 [file mmc5.pdf]
