# Supplementary material for: Genomic analysis of Poxviridae and exploring qualified gene sequences for phylogenetics
Source: Comput Struct Biotechnol J. 2021 Sep 28;19:5479–86. doi: 10.1016/j.csbj.2021.09.031 (PMC8515299; doi:10.1016/j.csbj.2021.09.031)

**Supplementary file 2:** The ML-Tree based on single nucleotide sequence of core genes. The numbers on the branches represent branch lengths/genetic distances and numbers below the branch points represent bootstrap values. The color of the branch endpoints represents the classification results based on synteny analysis.

**Legend**

- **Group *Ch*-A1**
- **Group *Ch*-A2**
- **Group *Ch*-B**
- **Group *Ch*-C**
- **Group *Ch*-D**
- **Group *Ch*-X**
- **Group *En-A***
- **NA**

The ML-Tree based on CG #1 nucleotide sequence

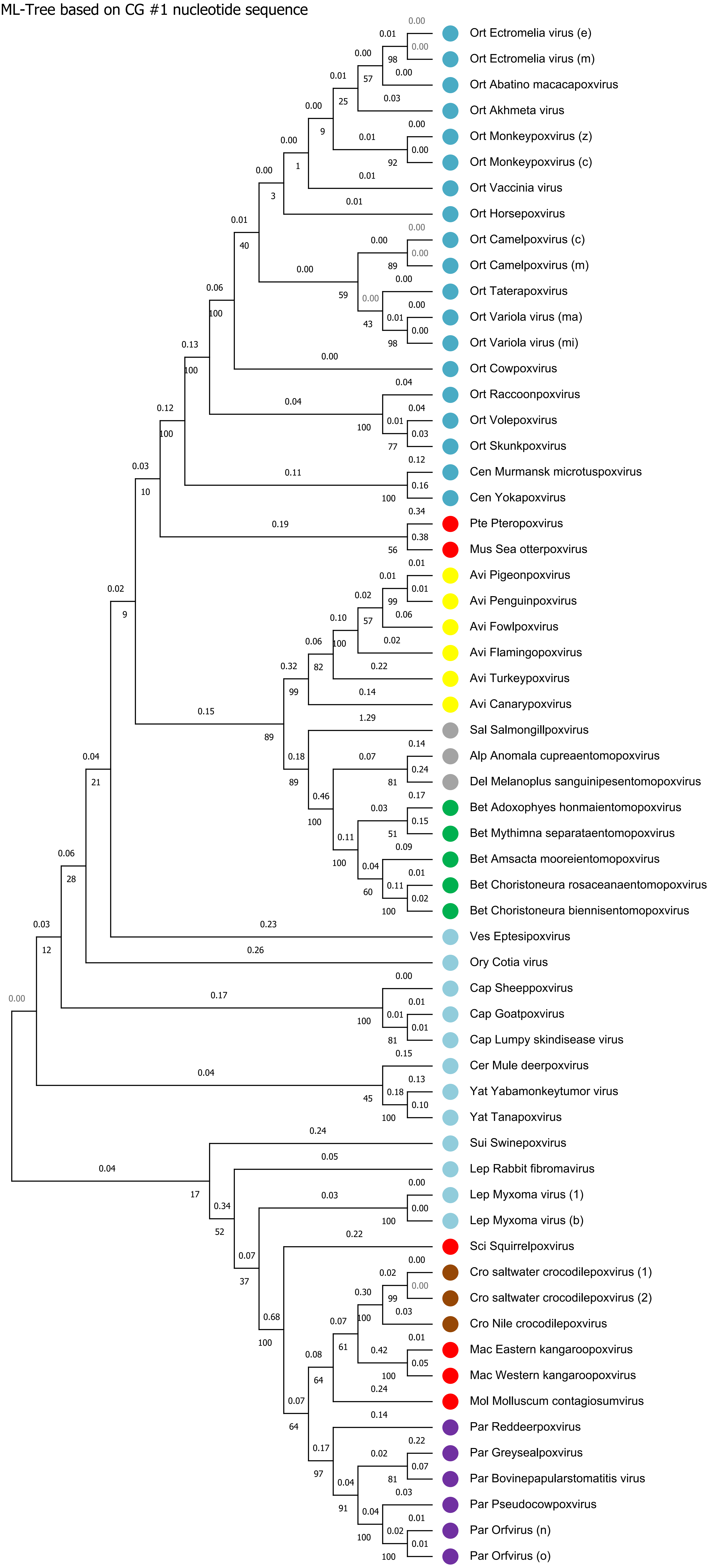

The ML-Tree based on CG #2 nucleotide sequence

Experience substantial substitution saturation

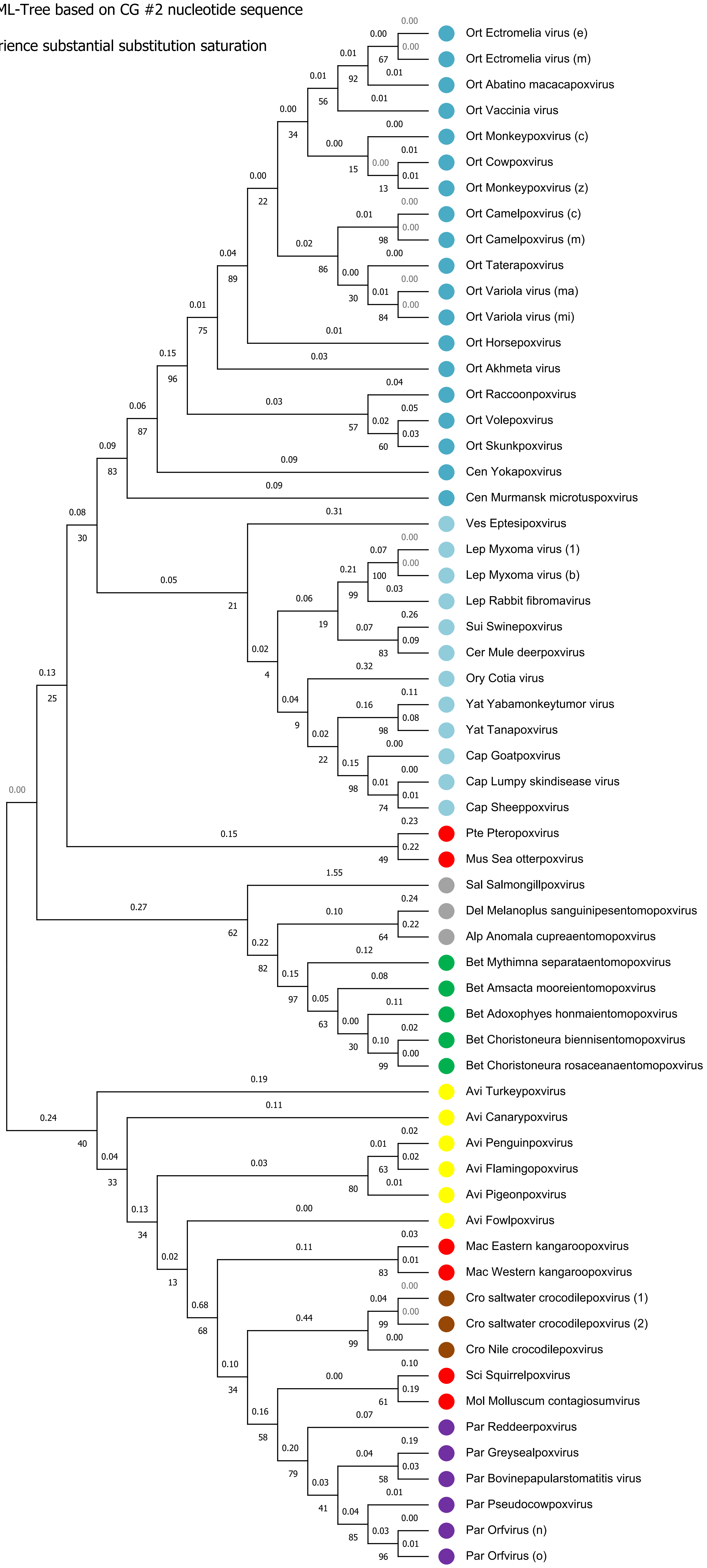

The ML-Tree based on CG #3 nucleotide sequence

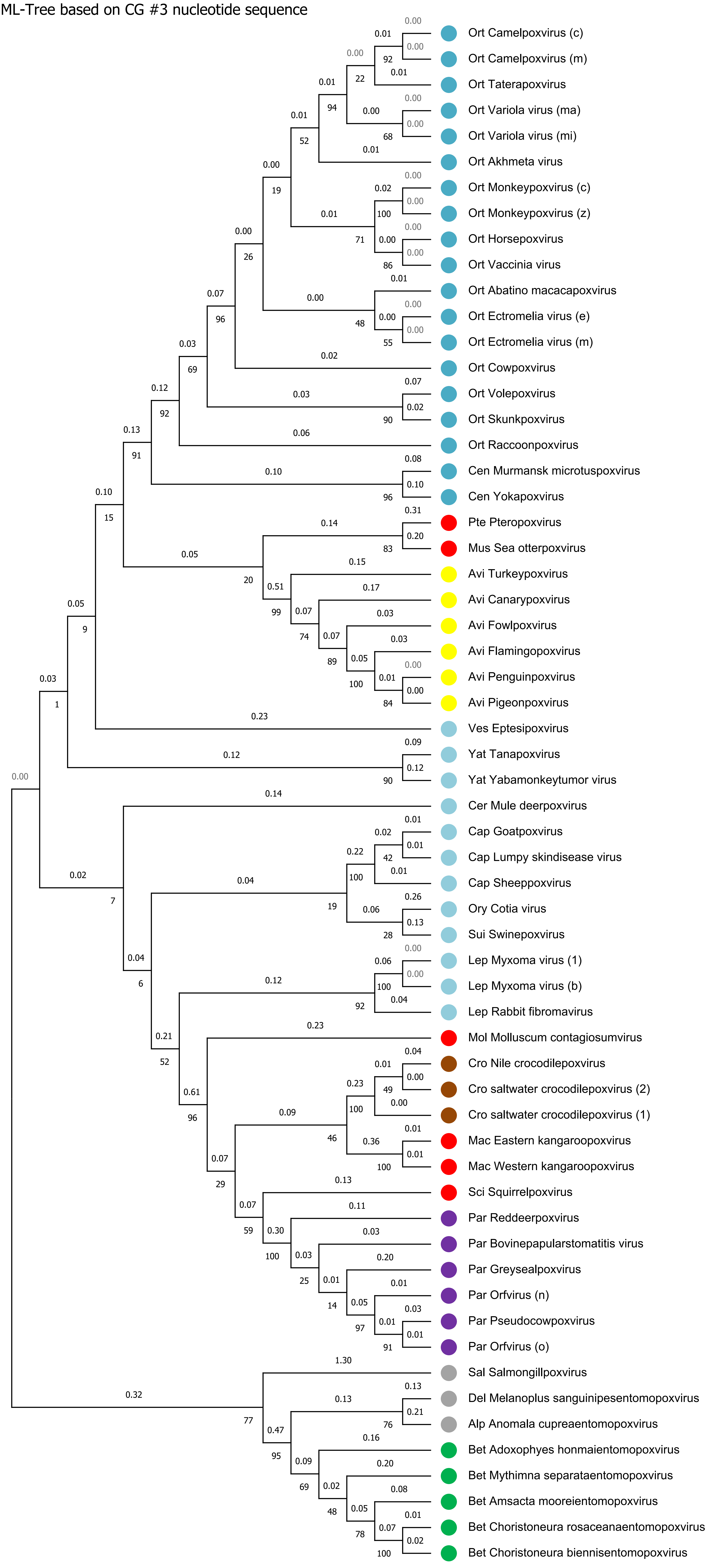

The ML-Tree based on CG #4 nucleotide sequence

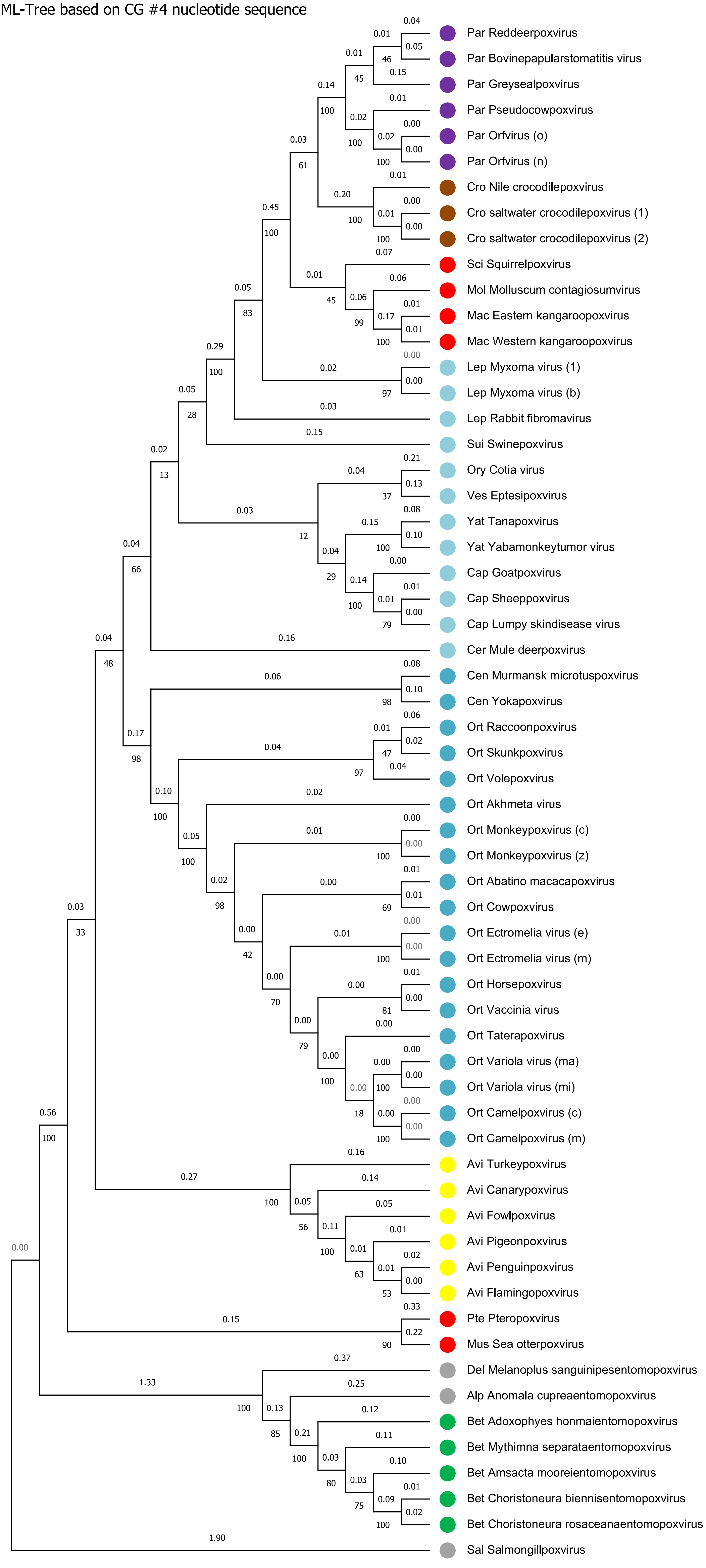

The ML-Tree based on CG #5 nucleotide sequence

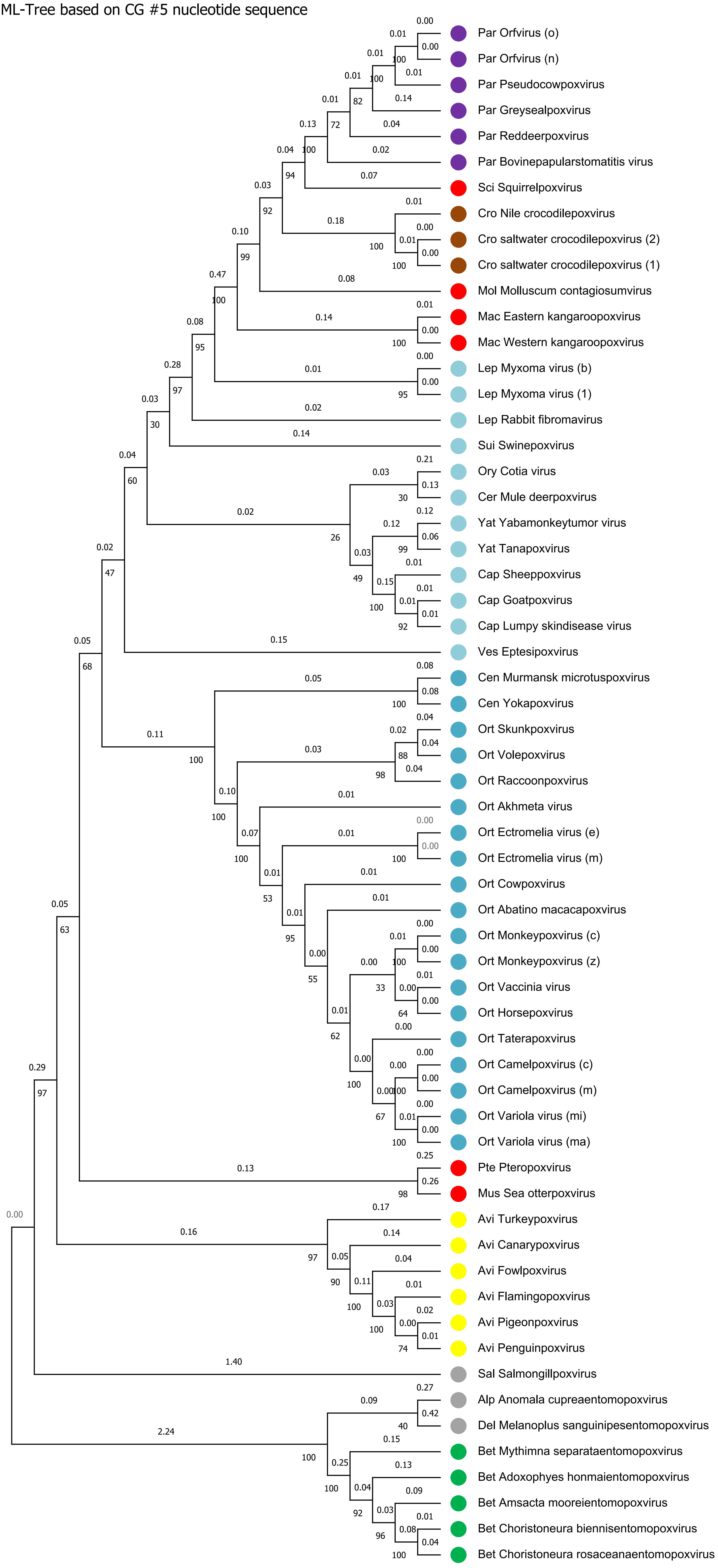

The ML-Tree based on CG #6 nucleotide sequence

Experience substantial substitution saturation

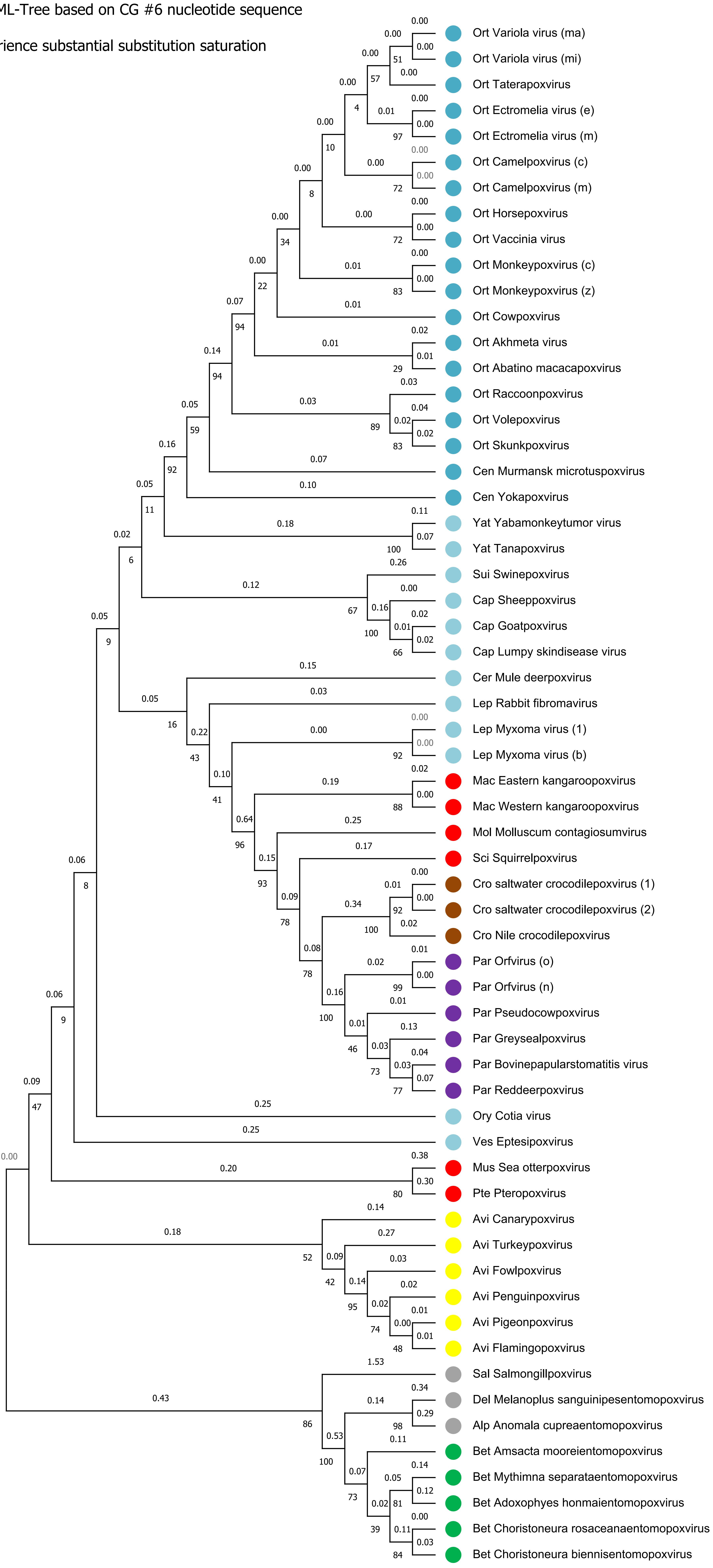

The ML-Tree based on CG #7 nucleotide sequence

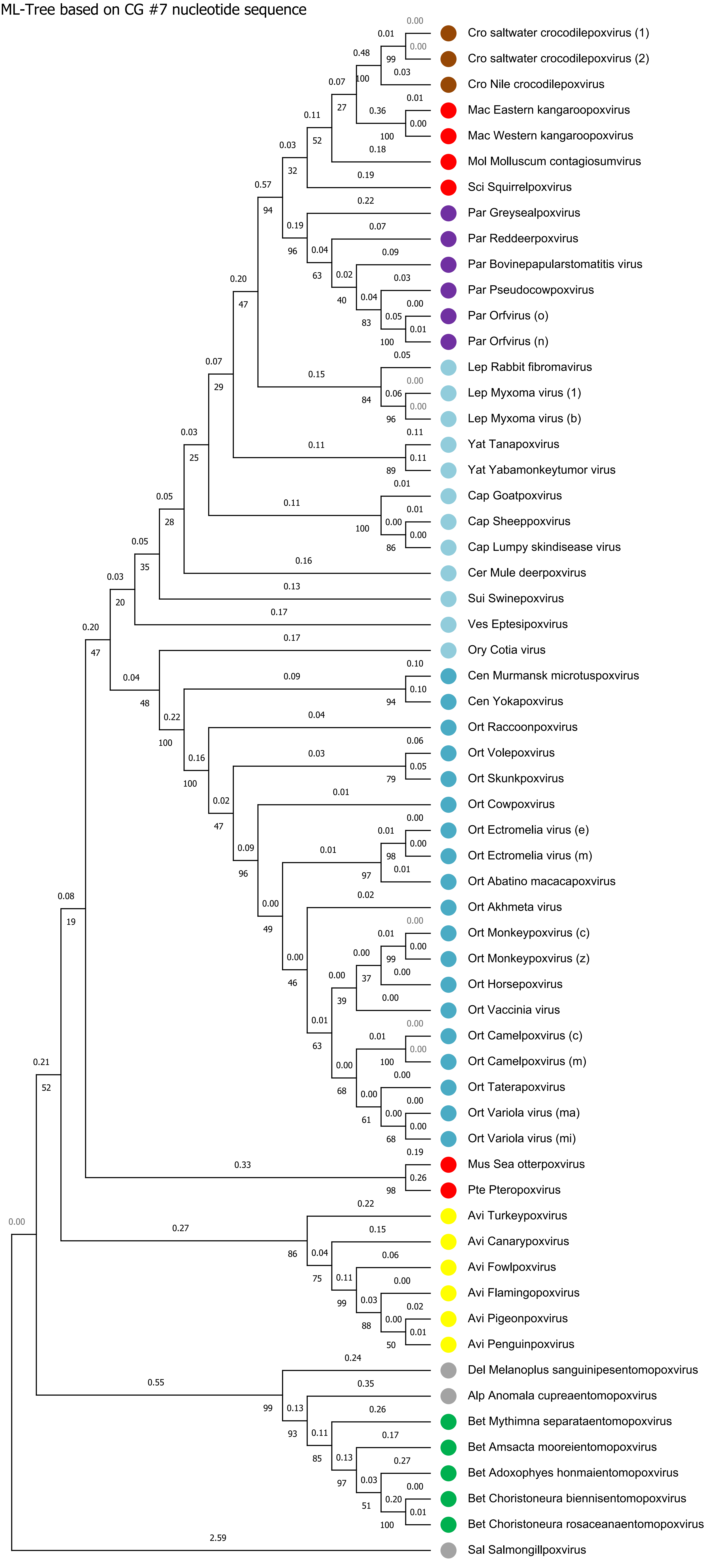

The ML-Tree based on CG #8 nucleotide sequence

Experience substantial substitution saturation

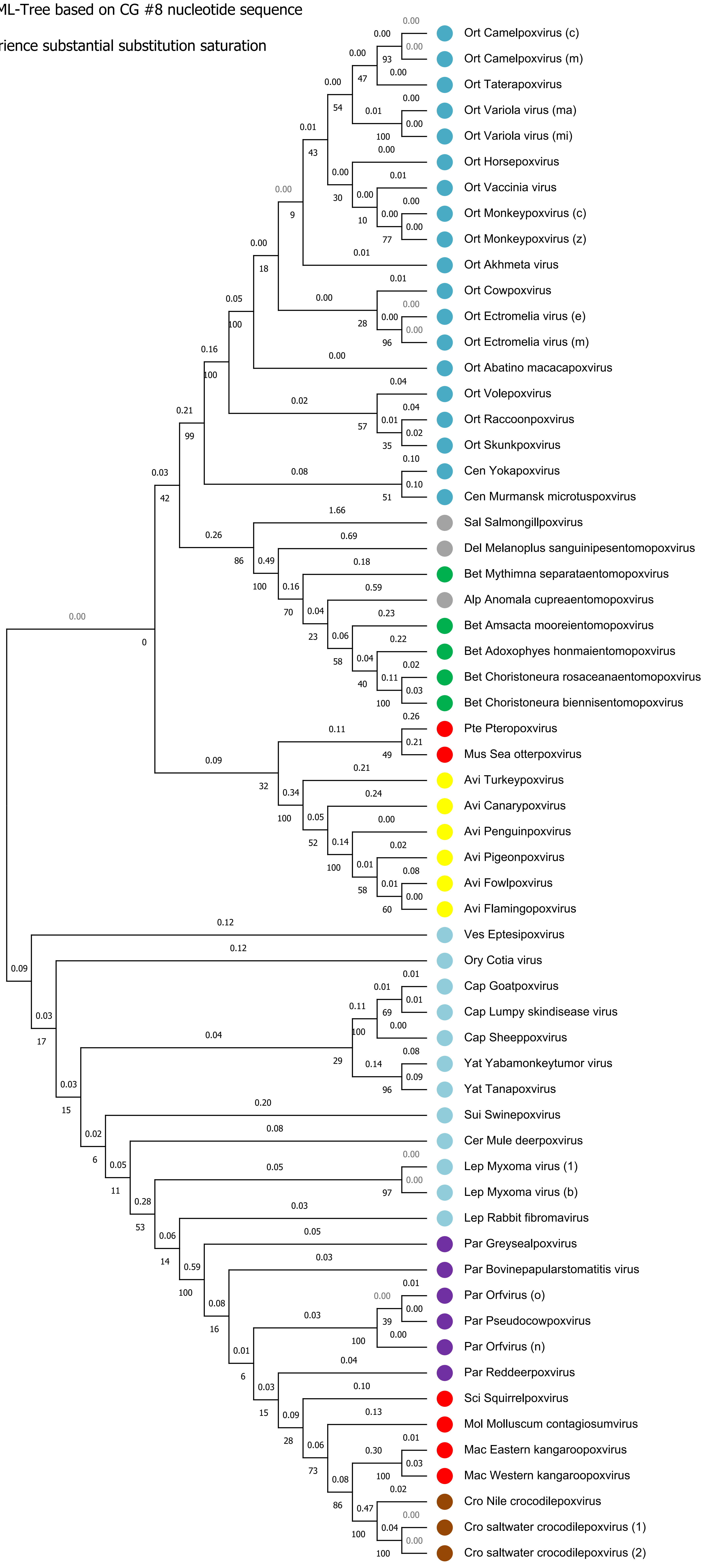

The ML-Tree based on CG #9 nucleotide sequence

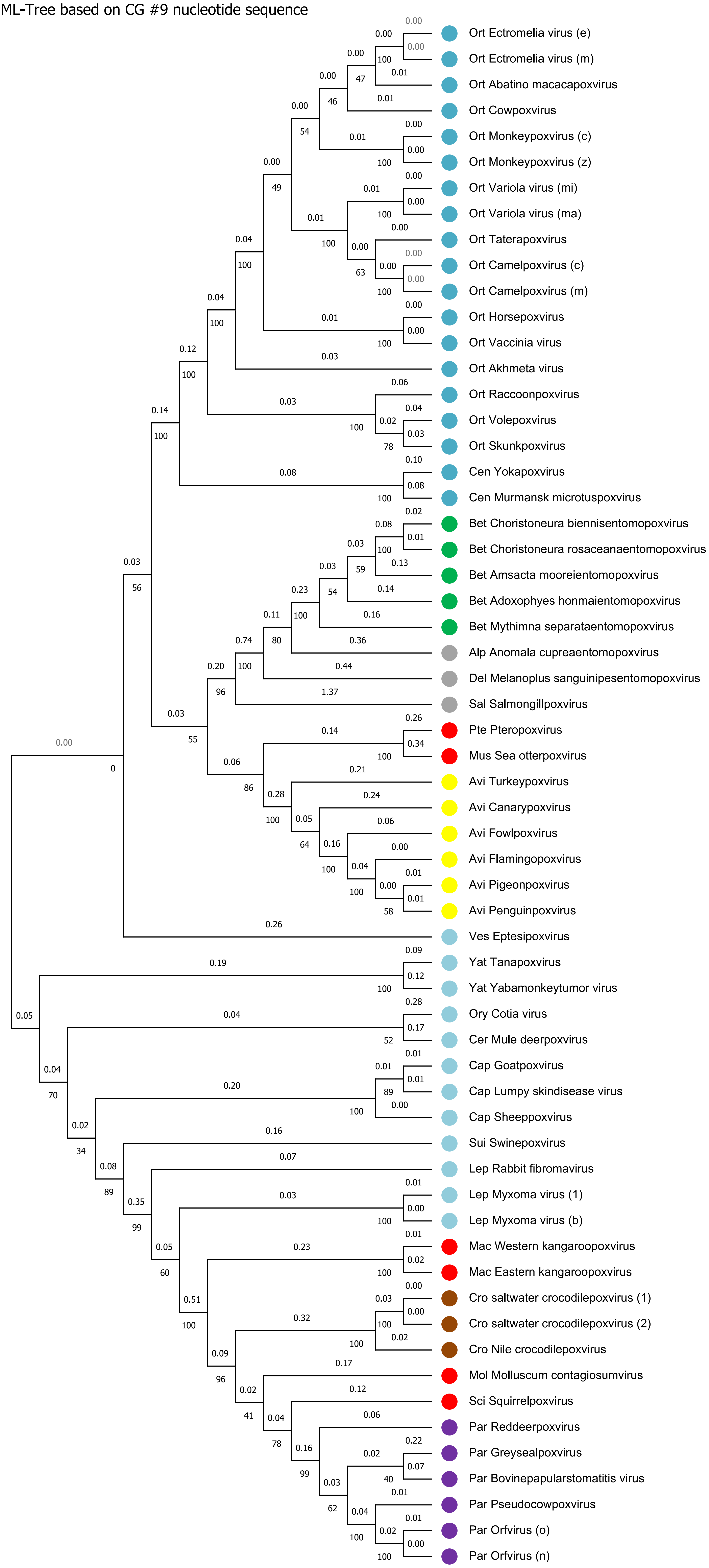

The ML-Tree based on CG #10 nucleotide sequence

Experience substantial substitution saturation

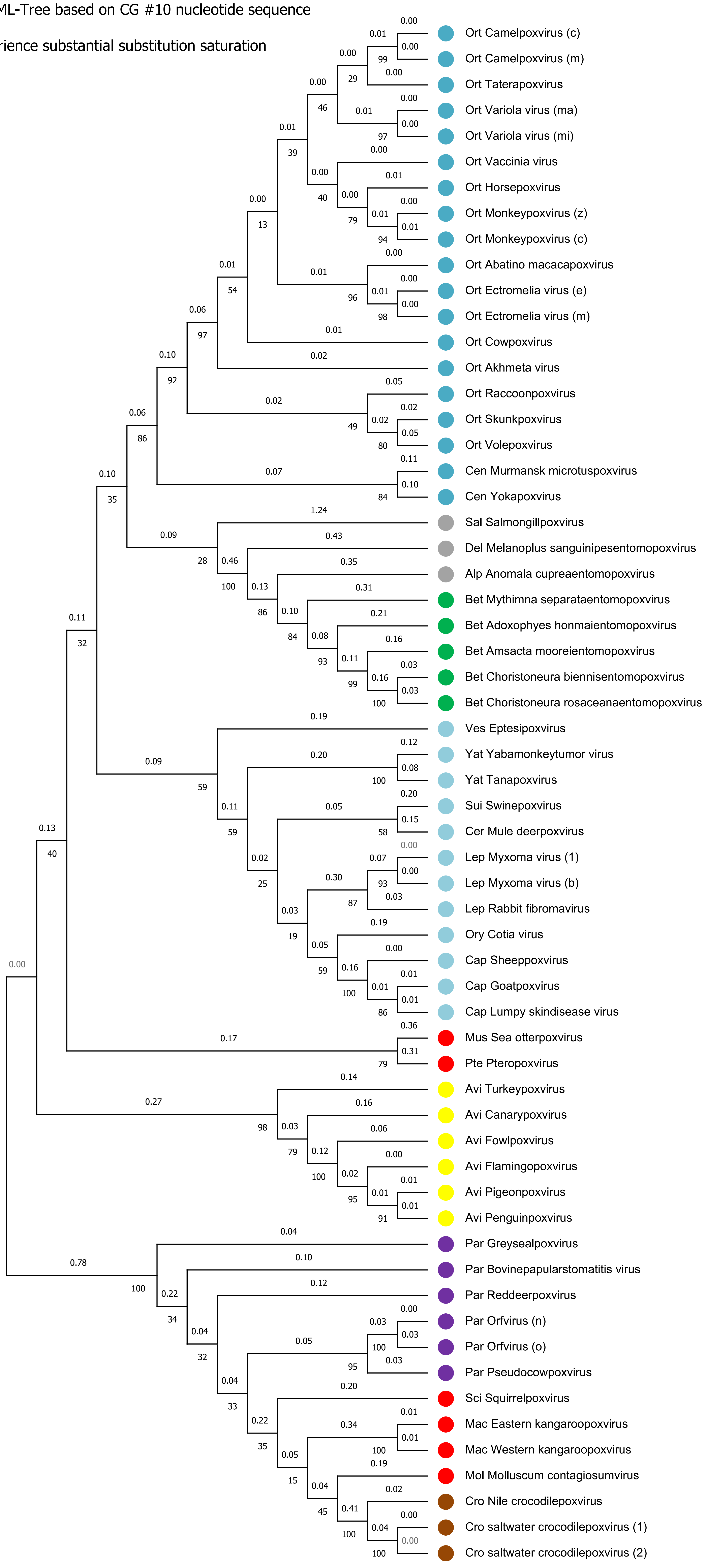

The ML-Tree based on CG #11 nucleotide sequence

Experience substantial substitution saturation

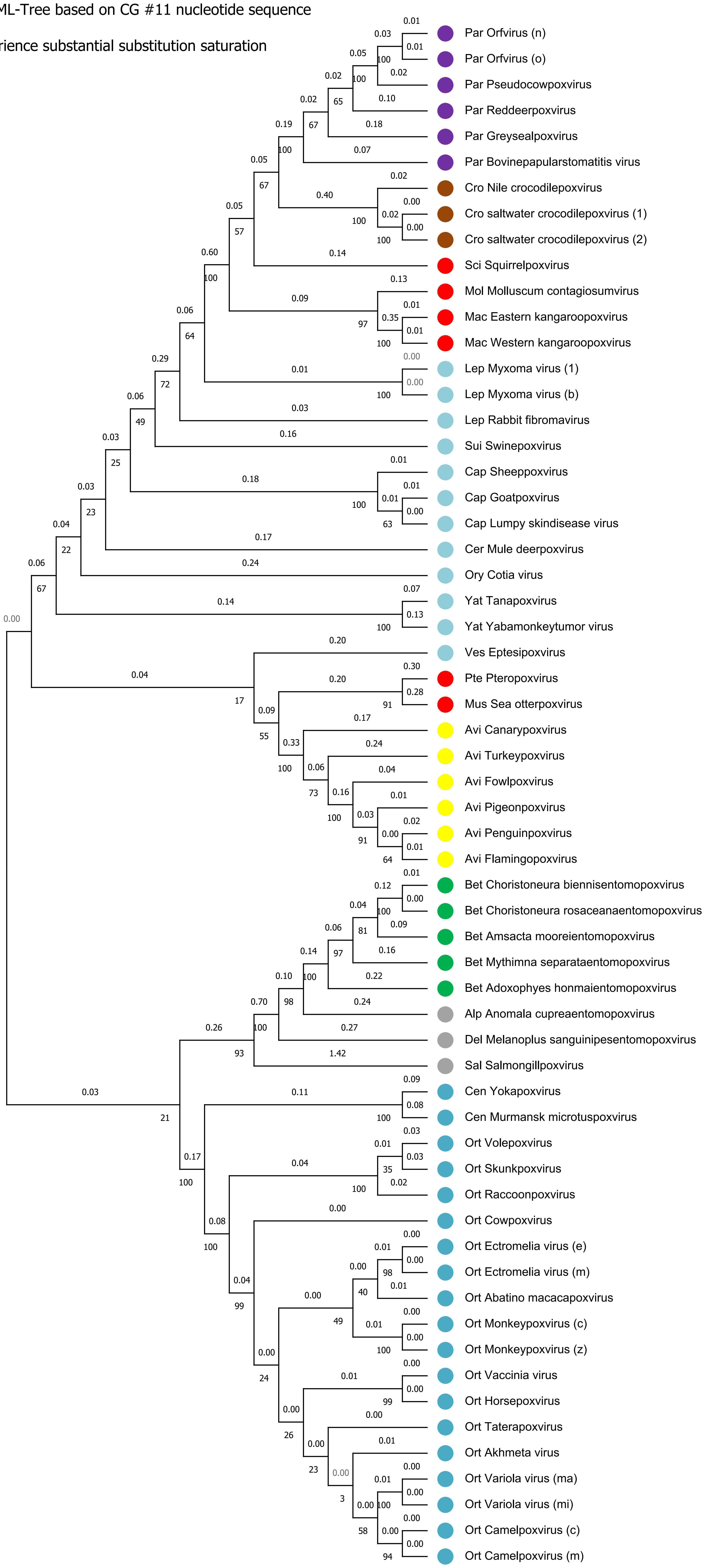

The ML-Tree based on CG #12 nucleotide sequence

Experience substantial substitution saturation

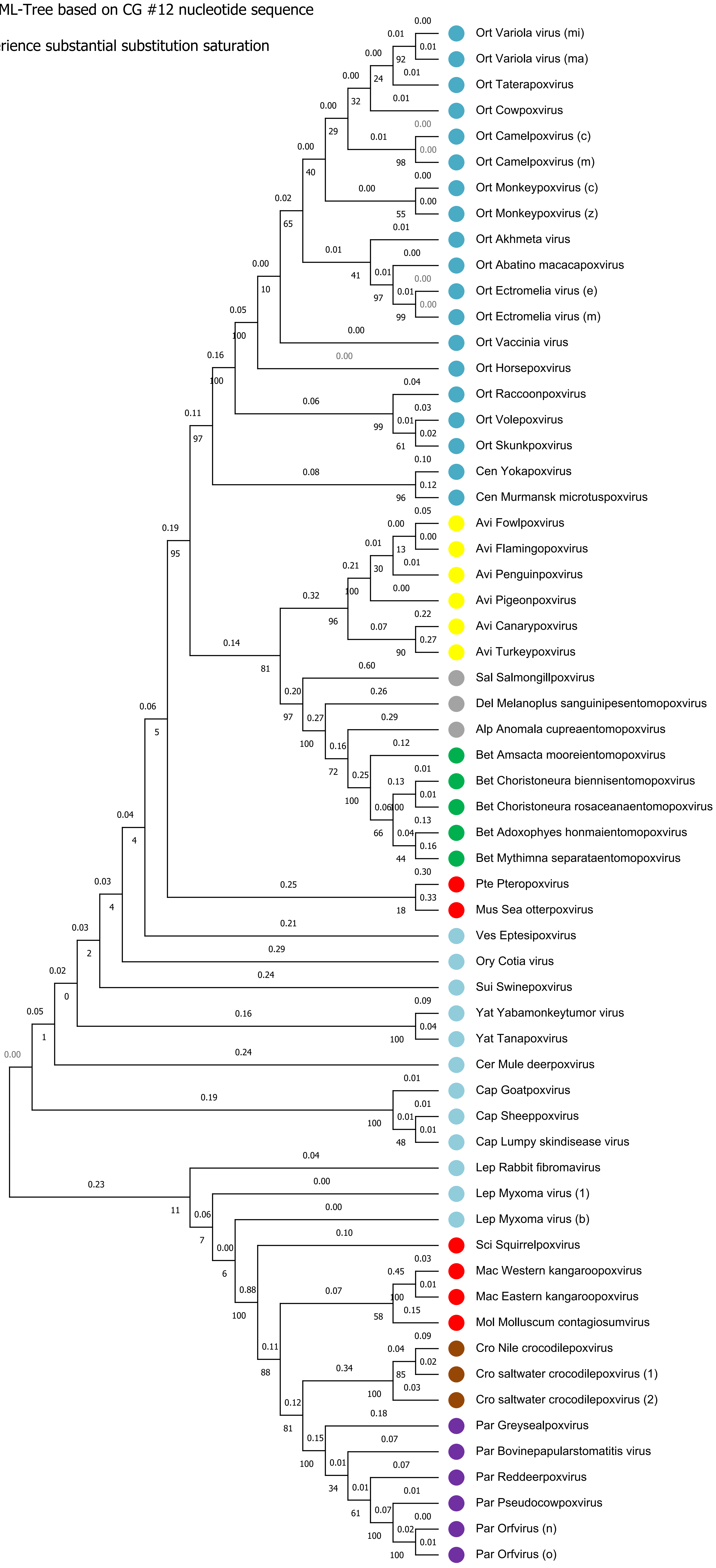

The ML-Tree based on CG #13 nucleotide sequence

Experience substantial substitution saturation

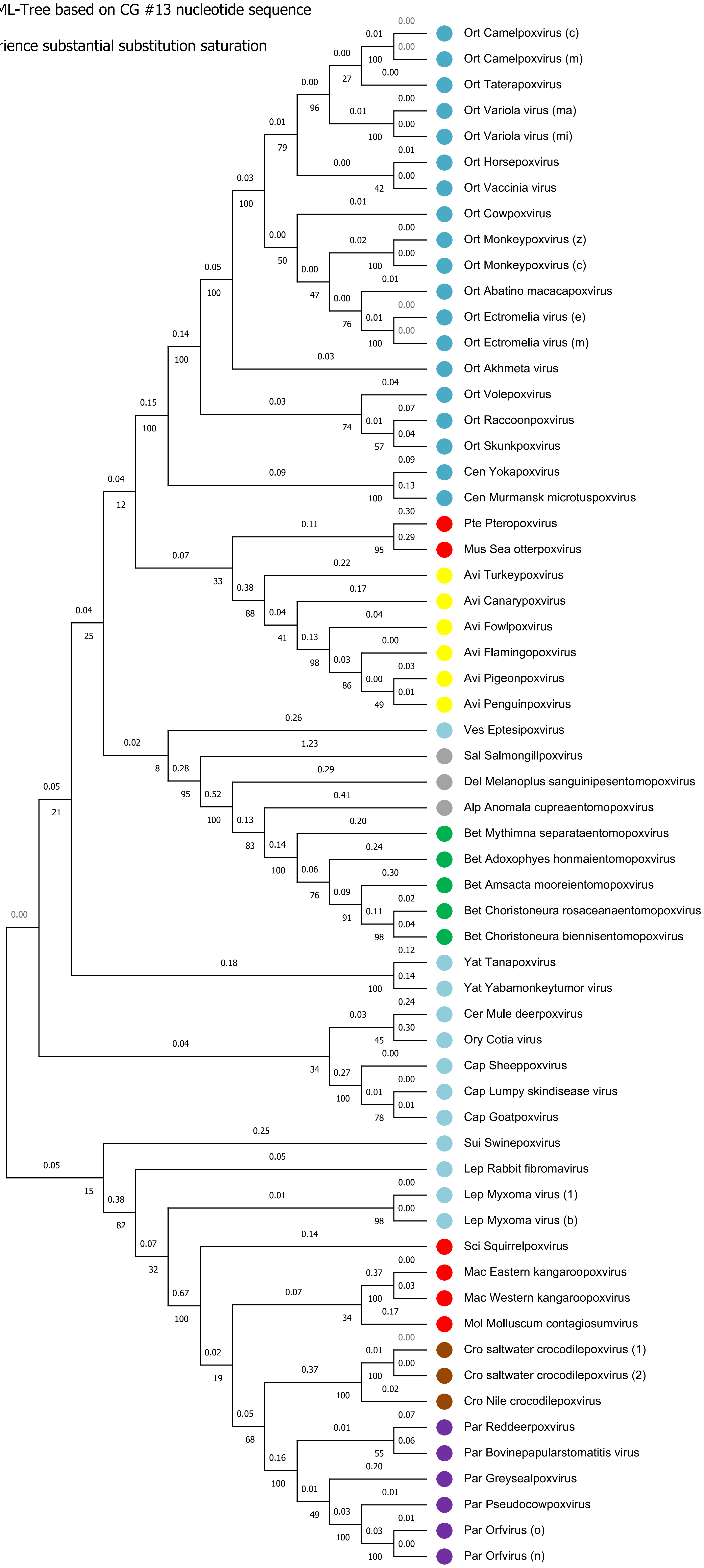

The ML-Tree based on CG #14 nucleotide sequence

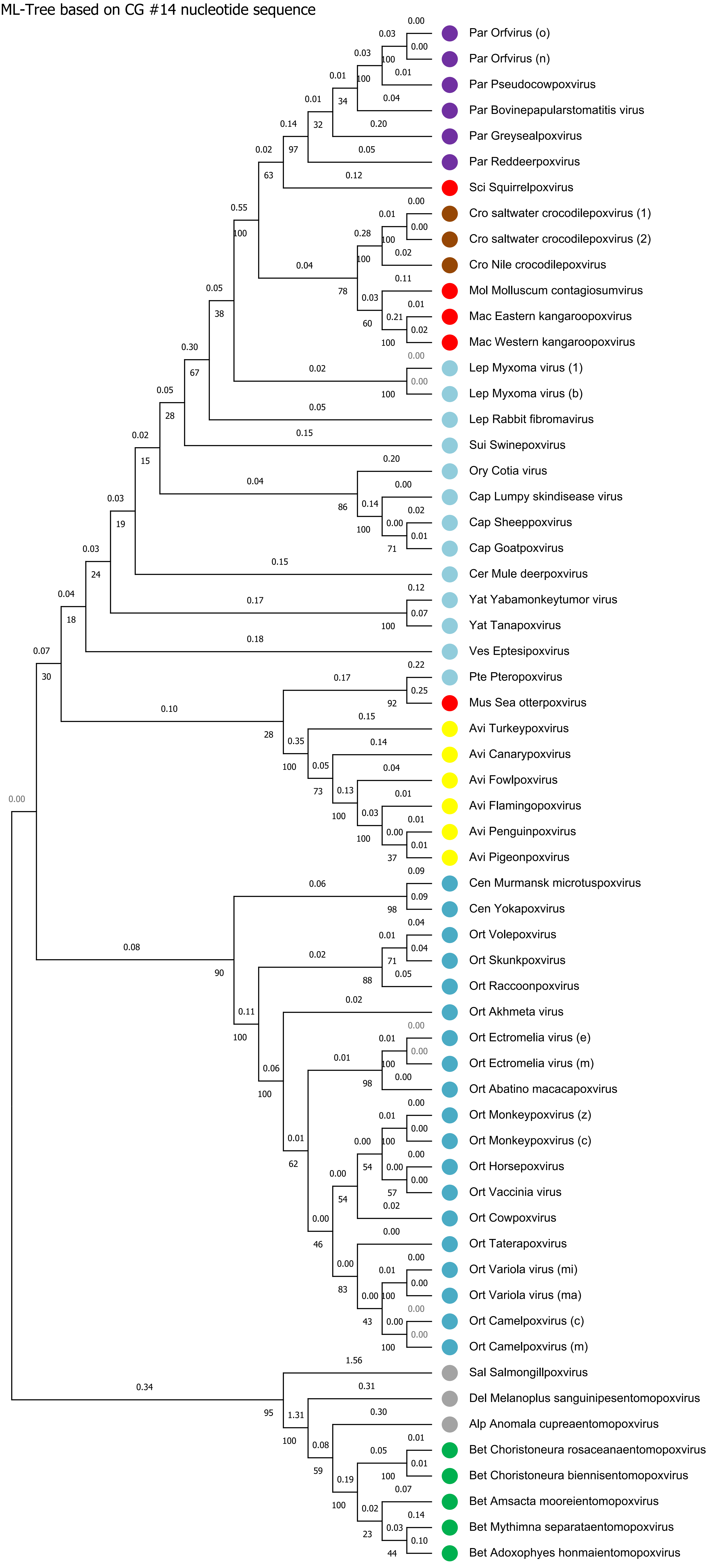

The ML-Tree based on CG #15 nucleotide sequence

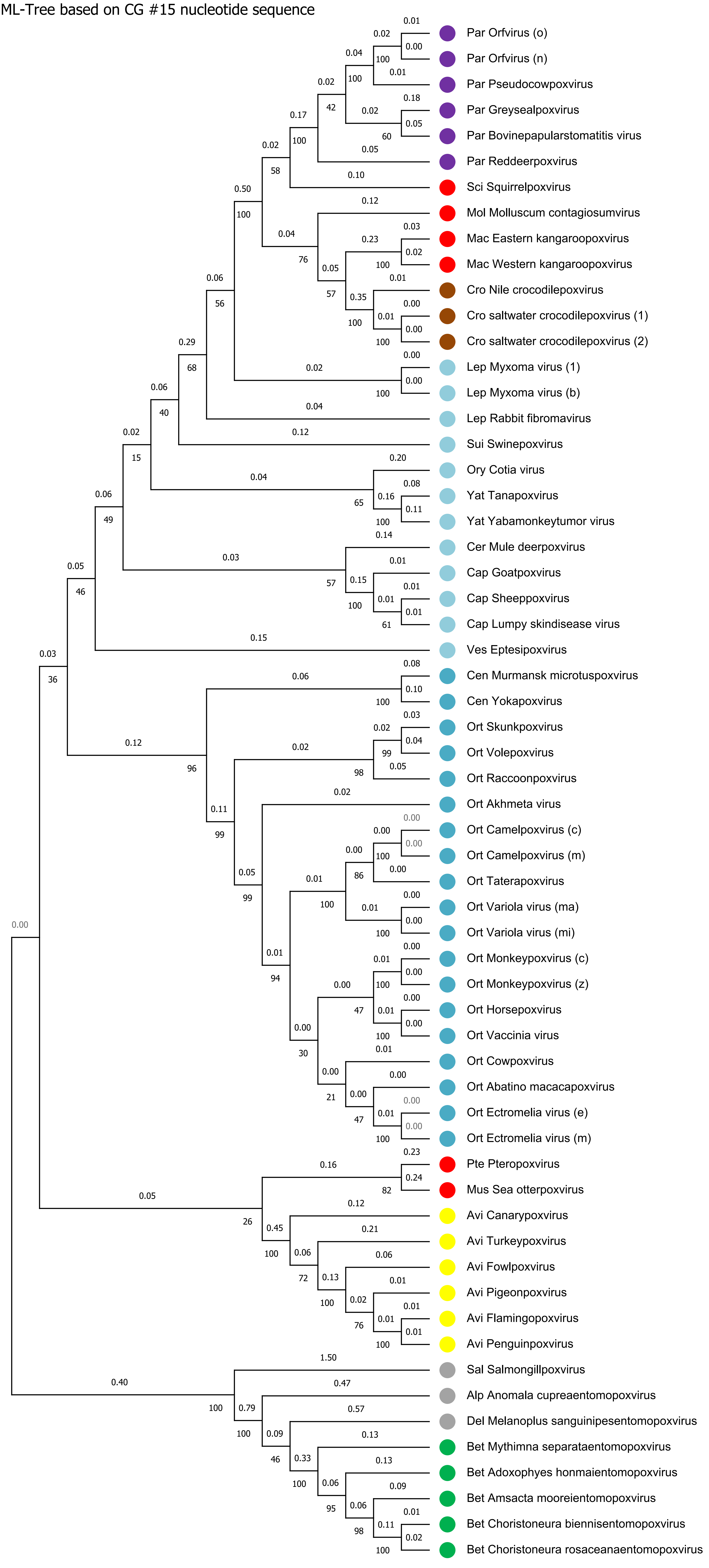

The ML-Tree based on CG #16 nucleotide sequence

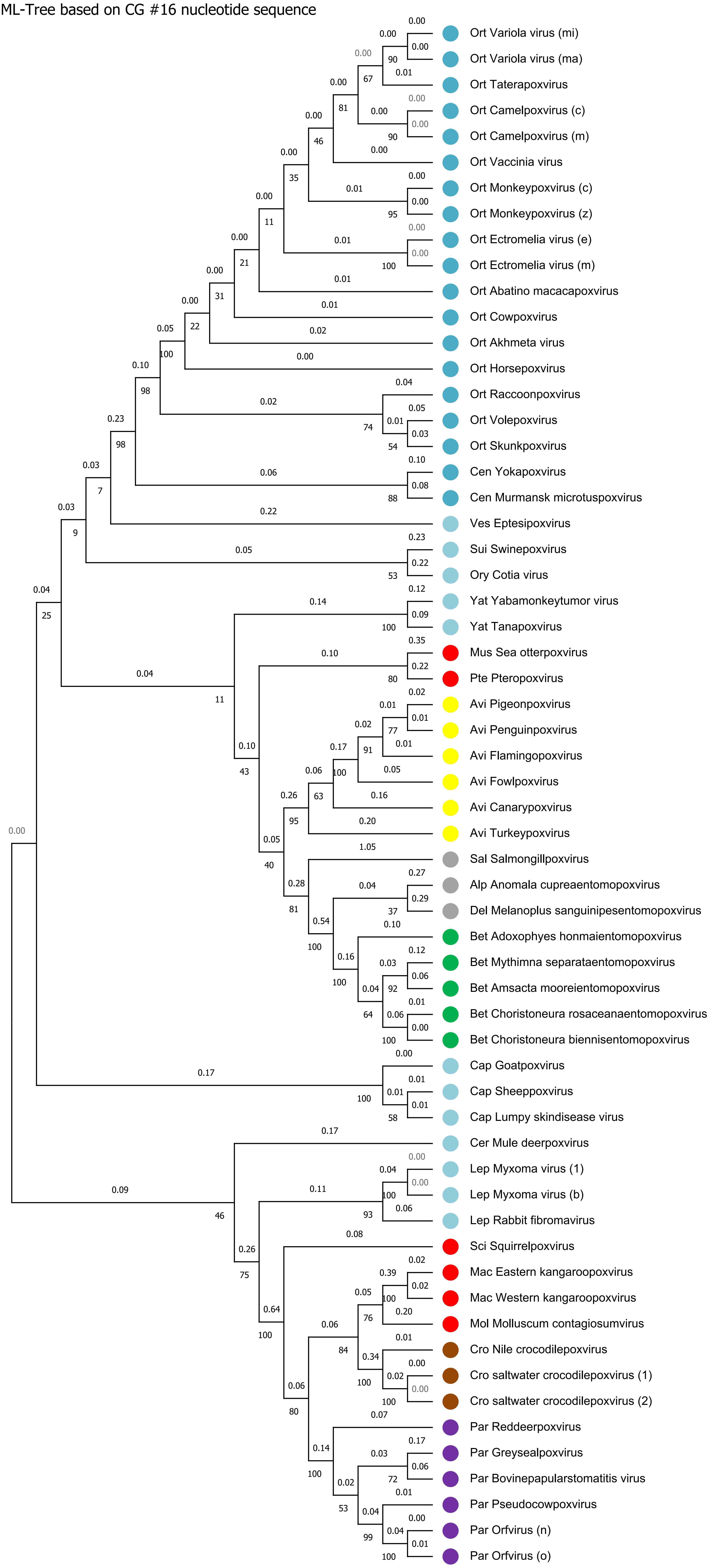

The ML-Tree based on CG #17 nucleotide sequence

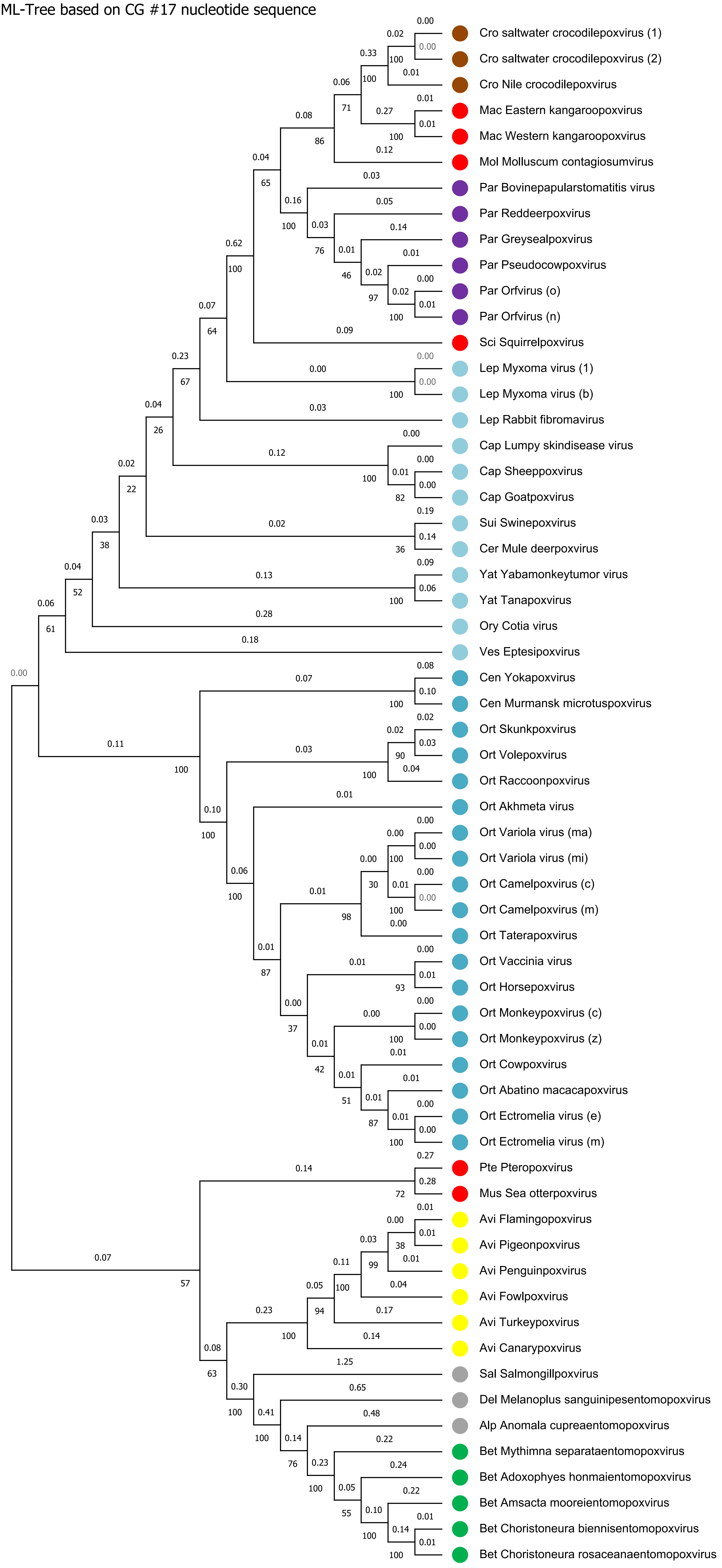

The ML-Tree based on CG #18 nucleotide sequence

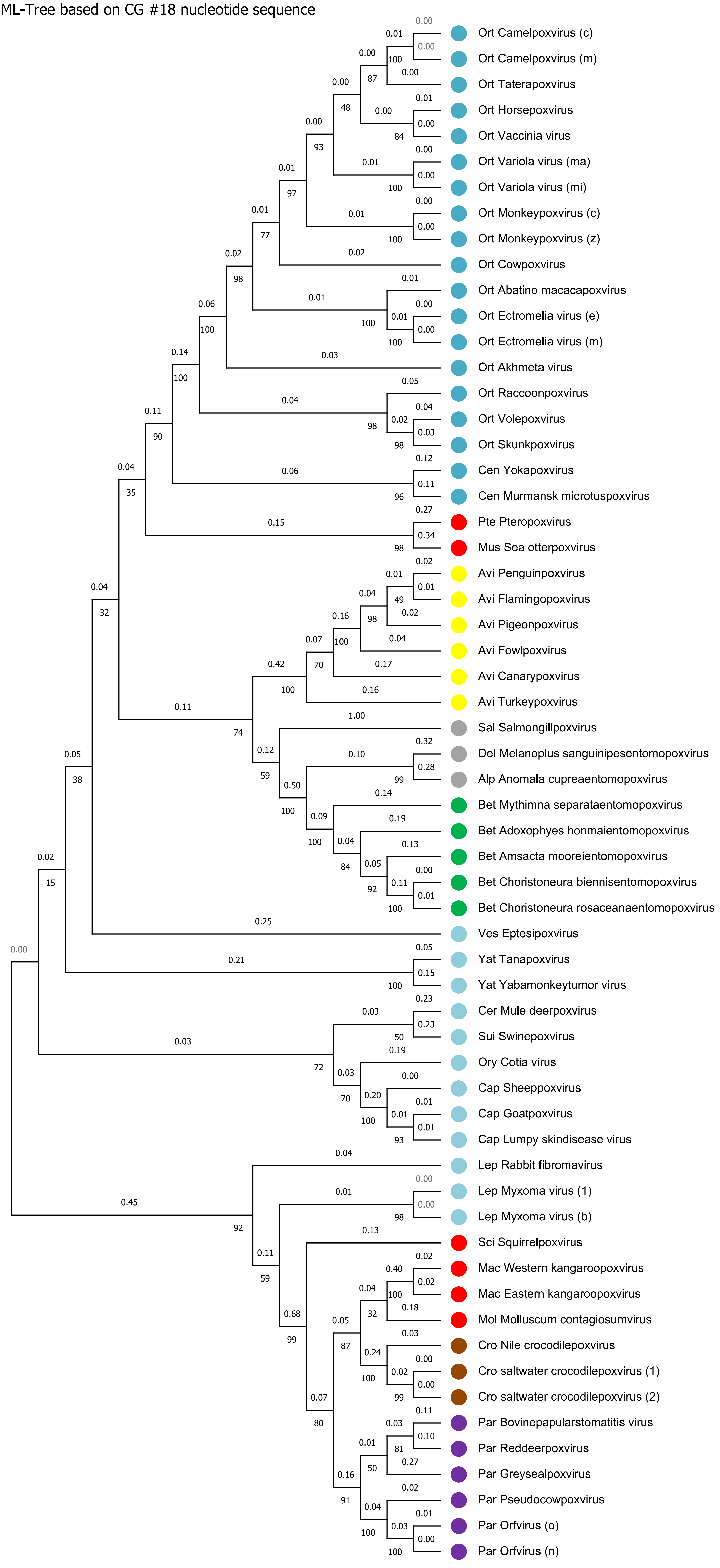

The ML-Tree based on CG #19 nucleotide sequence

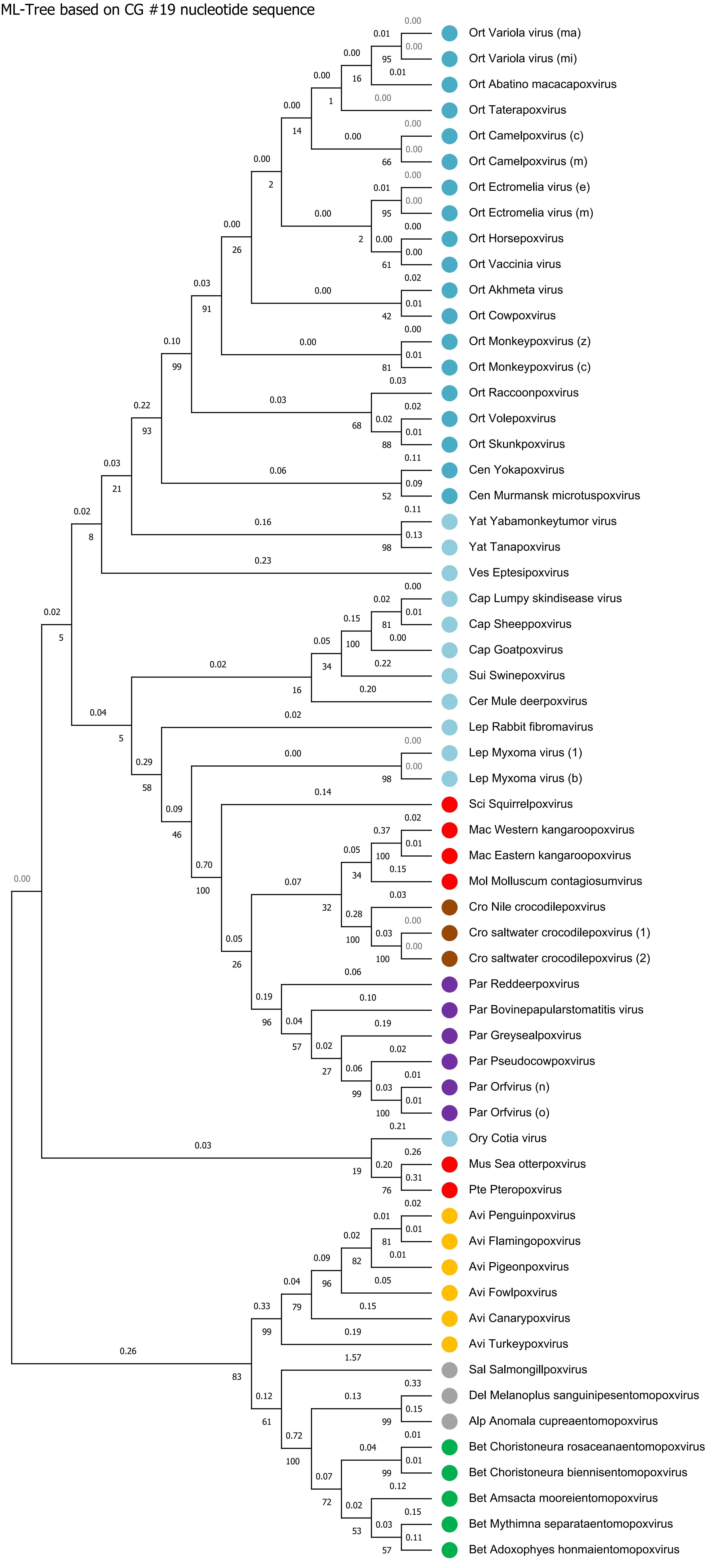

The ML-Tree based on CG #20 nucleotide sequence

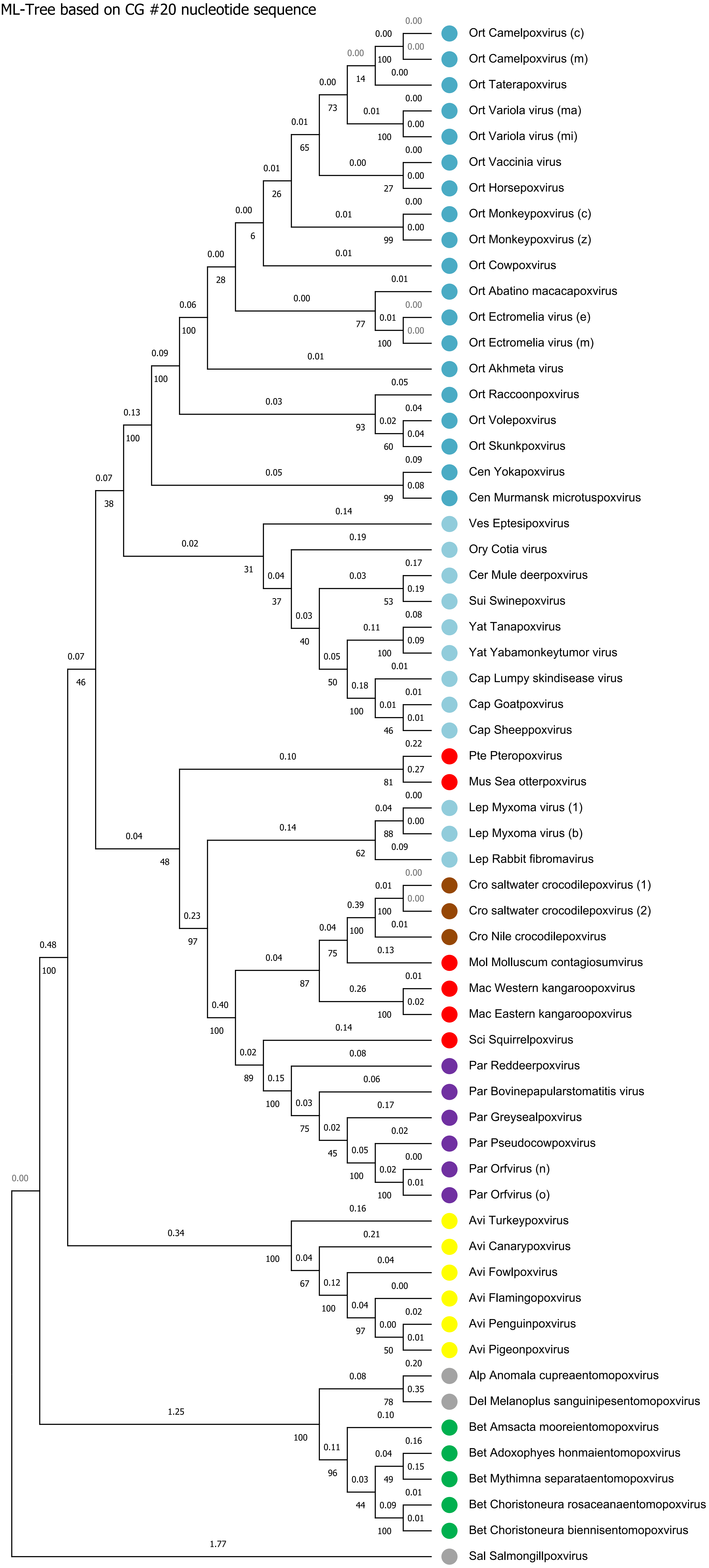

The ML-Tree based on CG #21 nucleotide sequence

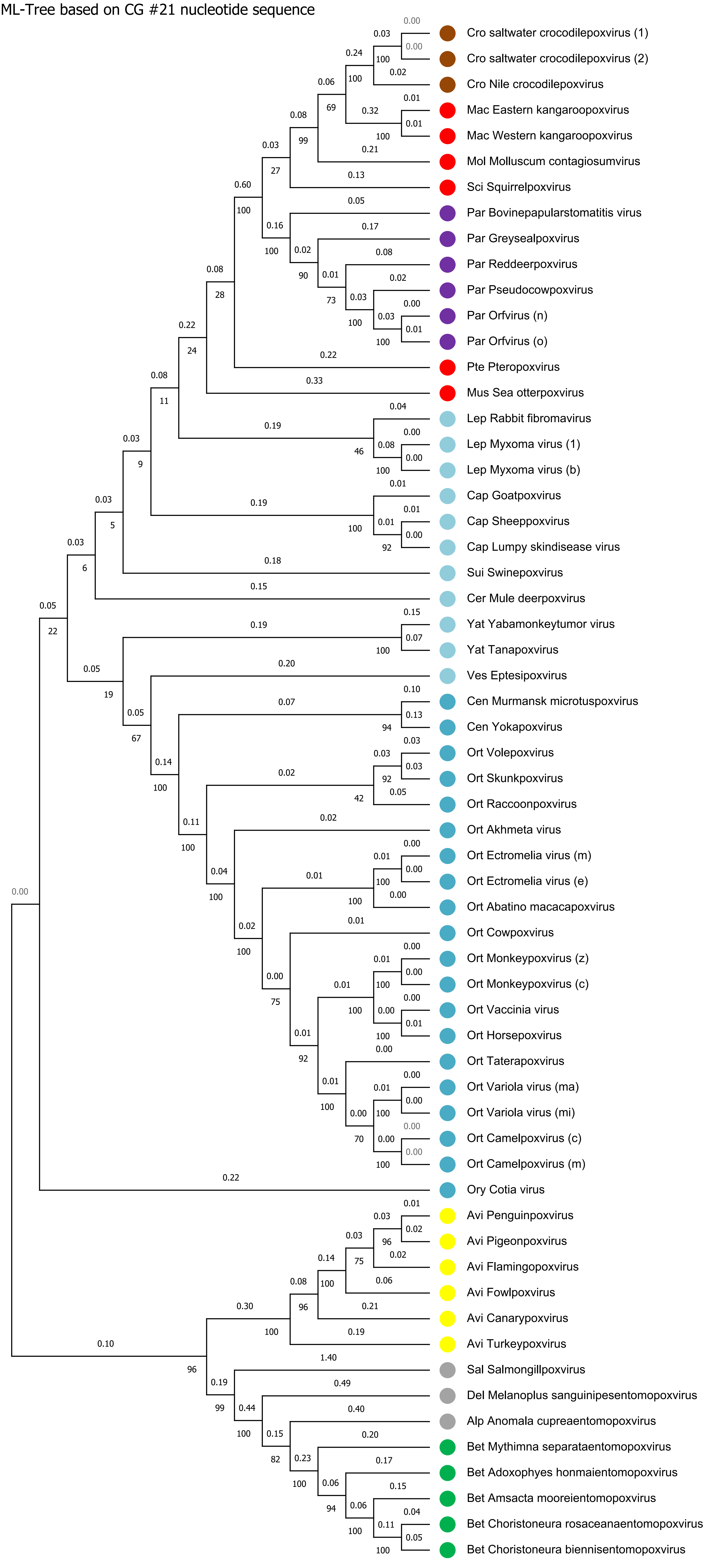

The ML-Tree based on CG #22 nucleotide sequence

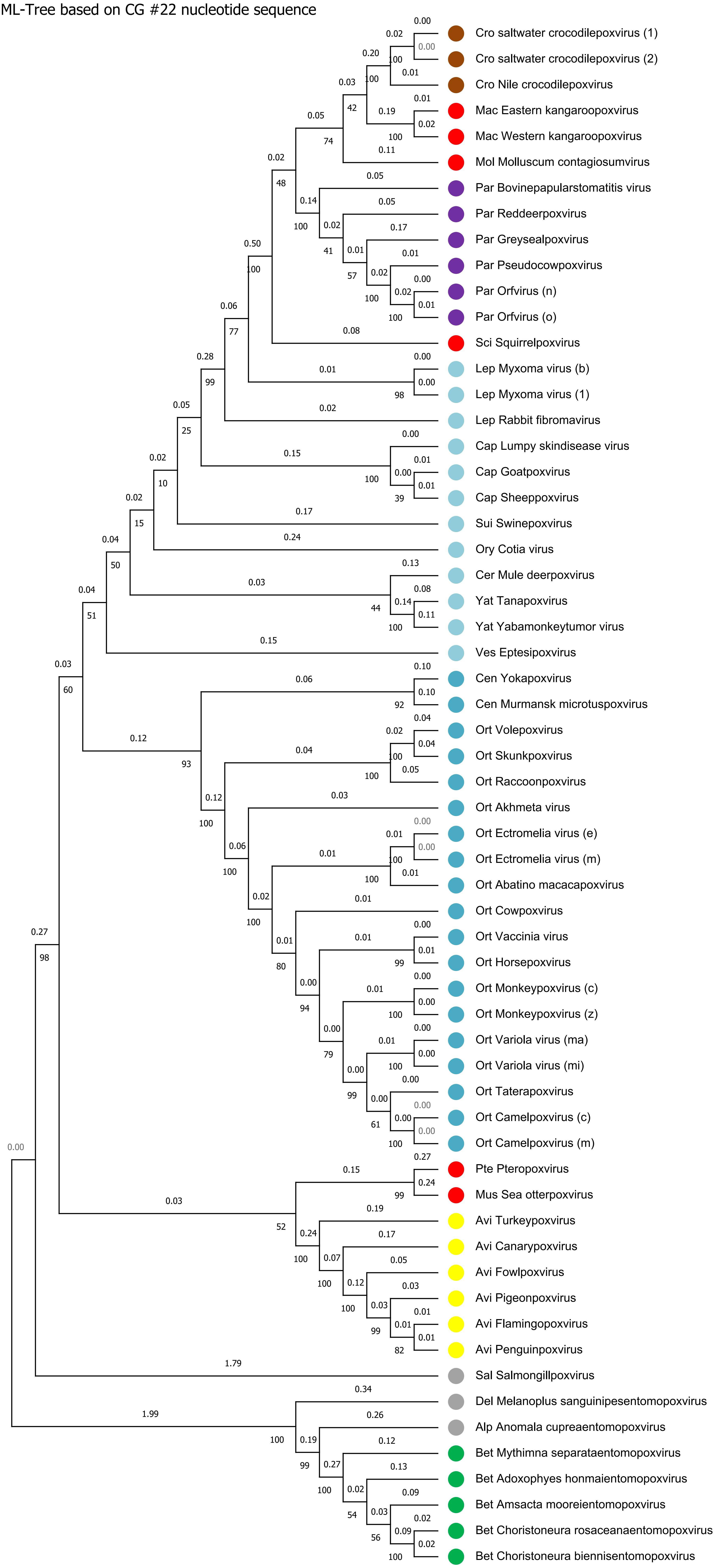

Supplement: Supplementary data 6 [file mmc6.pdf]
