# Supplementary material for: Genomic analysis of Poxviridae and exploring qualified gene sequences for phylogenetics
Source: Comput Struct Biotechnol J. 2021 Sep 28;19:5479–86. doi: 10.1016/j.csbj.2021.09.031 (PMC8515299; doi:10.1016/j.csbj.2021.09.031)

Supplementary file 3: The NJ-Tree based on single amino acid sequence of core genes. The numbers on the branches represent branch lengths/genetic distances and numbers below the branch points represent bootstrap values. The color of the branch endpoints represents the classification results based on synteny analysis.

Legend

- **Group *Ch*-A1**
- **Group *Ch*-A2**
- **Group *Ch*-B**
- **Group *Ch*-C**
- **Group *Ch*-D**
- **Group *Ch*-X**
- **Group *En-A***
- **NA**

The NJ-Tree based on CG #1 amino acid sequence

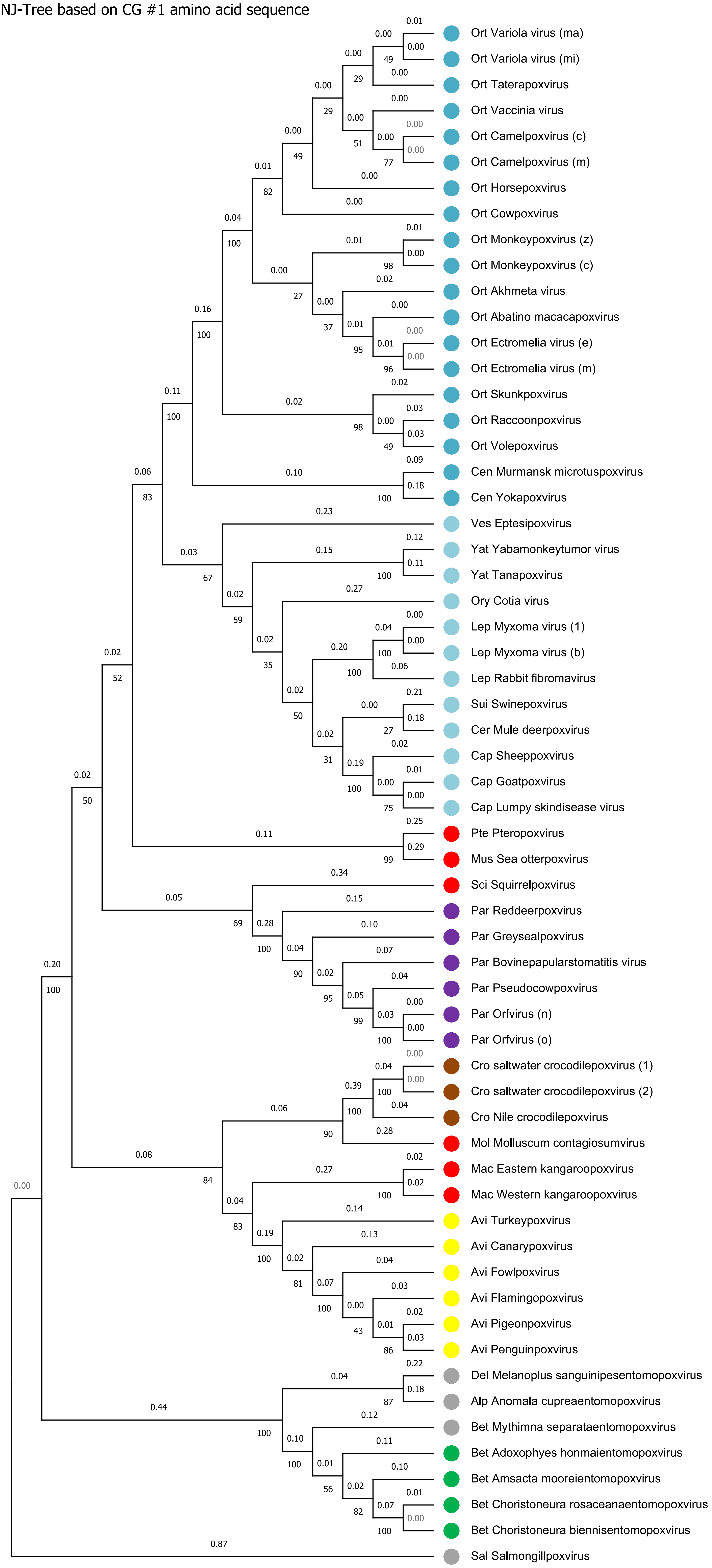

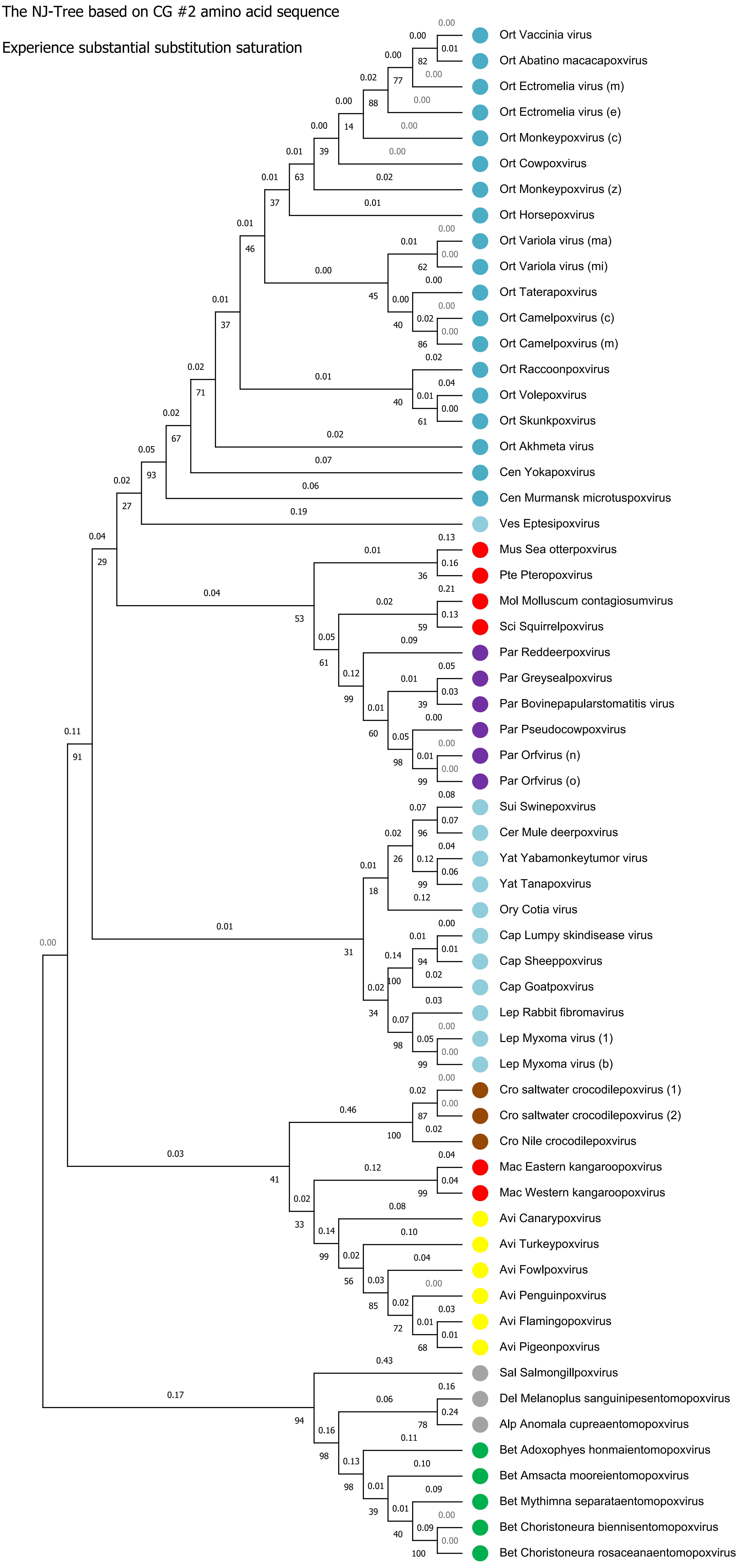
The NJ-Tree based on CG #2 amino acid sequence
Experience substantial substitution saturation
Ort Vaccinia virus
Ort Abatino macacapoxvirus
Ort Ectromelia virus (m)
Ort Ectromelia virus (e)
Ort Monkeypoxvirus (c)
Ort Cowpoxvirus
Ort Monkeypoxvirus (z)
Ort Horsepoxvirus
Ort Variola virus (ma)
Ort Variola virus (mi)
Ort Taterapoxvirus
Ort Camelpoxvirus (c)
Ort Camelpoxvirus (m)
Ort Raccoonpoxvirus
Ort Volepoxvirus
Ort Skunkpoxvirus
Ort Akhmeta virus
Cen Yokapoxvirus
Cen Murmansk microtuspoxvirus
Ves Eptesipoxvirus
Mus Sea otterpoxvirus
Pte Pteropoxvirus
Mol Molluscum contagiosumvirus
Sci Squirrelpoxvirus
Par Reddeerpoxvirus
Par Greysealpoxvirus
Par Bovinepapularstomatitis virus
Par Pseudocowpoxvirus
Par Orfvirus (n)
Par Orfvirus (o)
Sui Swinepoxvirus
Cer Mule deerpoxvirus
Yat Yabamonkeytumor virus
Yat Tanapoxvirus
Ory Cotia virus
Cap Lumpy skindisease virus
Cap Sheeppoxvirus
Cap Goatpoxvirus
Lep Rabbit fibromavirus
Lep Myxoma virus (1)
Lep Myxoma virus (b)
Cro saltwater crocodilepoxvirus (1)
Cro saltwater crocodilepoxvirus (2)
Cro Nile crocodilepoxvirus
Mac Eastern kangaroopoxvirus
Mac Western kangaroopoxvirus
Avi Canarypoxvirus
Avi Turkeypoxvirus
Avi Fowlpoxvirus
Avi Penguinpoxvirus
Avi Flamingopoxvirus
Avi Pigeonpoxvirus
Sal Salmongillpoxvirus
Del Melanoplus sanguinipesentomopoxvirus
Alp Anomala cupreaentomopoxvirus
Bet Adoxophyes honmaientomopoxvirus
Bet Amsacta mooreientomopoxvirus
Bet Mythimna separataentomopoxvirus
Bet Choristoneura biennisentomopoxvirus
Bet Choristoneura rosaceanaentomopoxvirus

The NJ-Tree based on CG #3 amino acid sequence

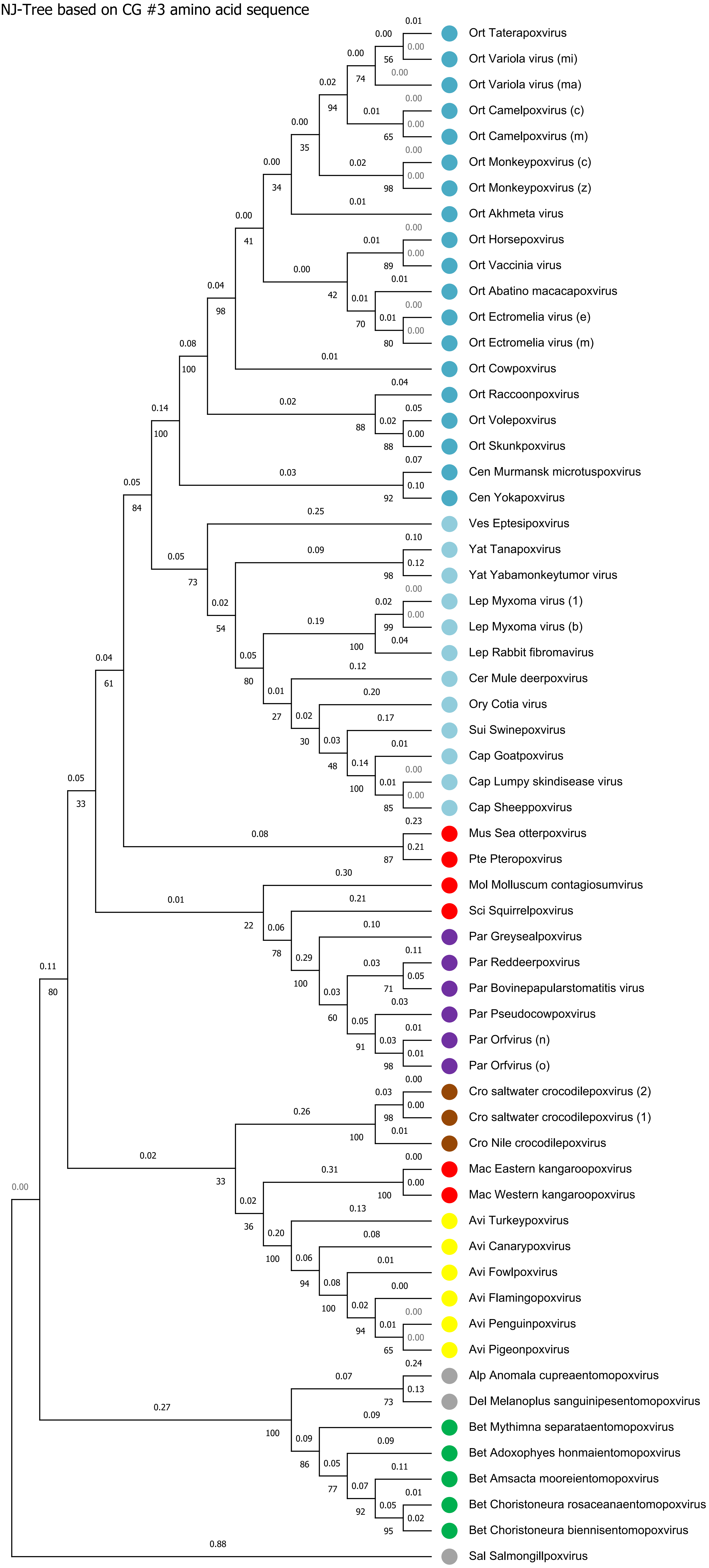

The NJ-Tree based on CG #4 amino acid sequence

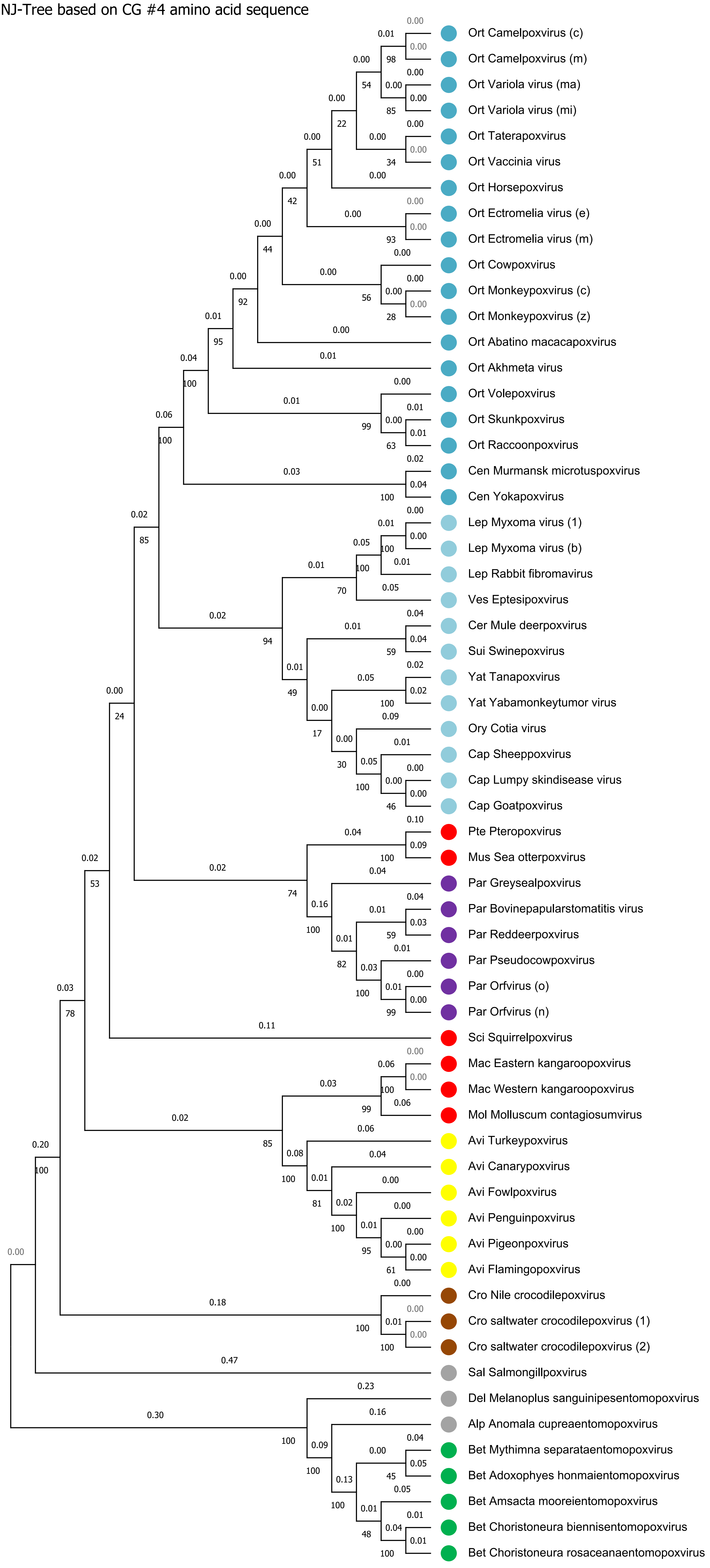

The NJ-Tree based on CG #5 amino acid sequence

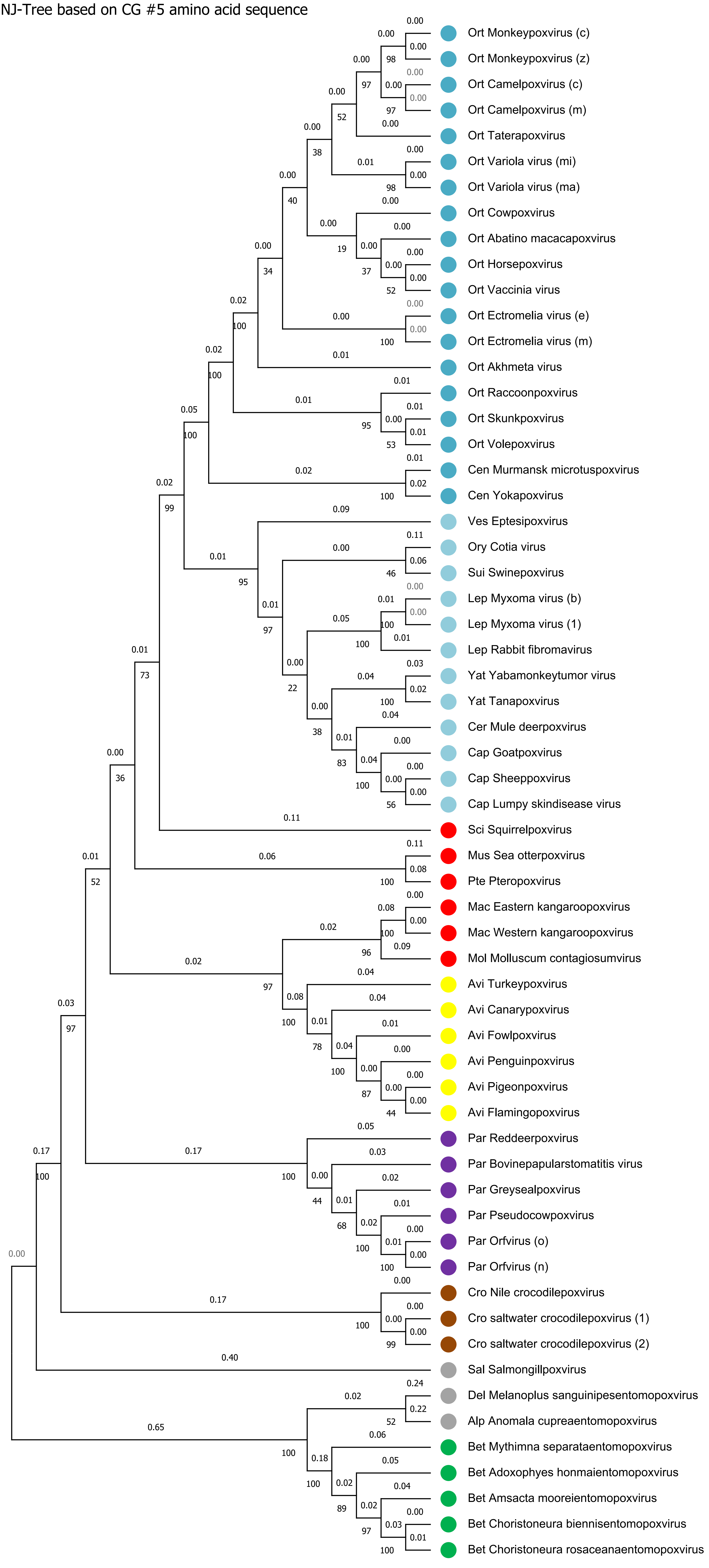

The NJ-Tree based on CG #6 amino acid sequence

Experience substantial substitution saturation

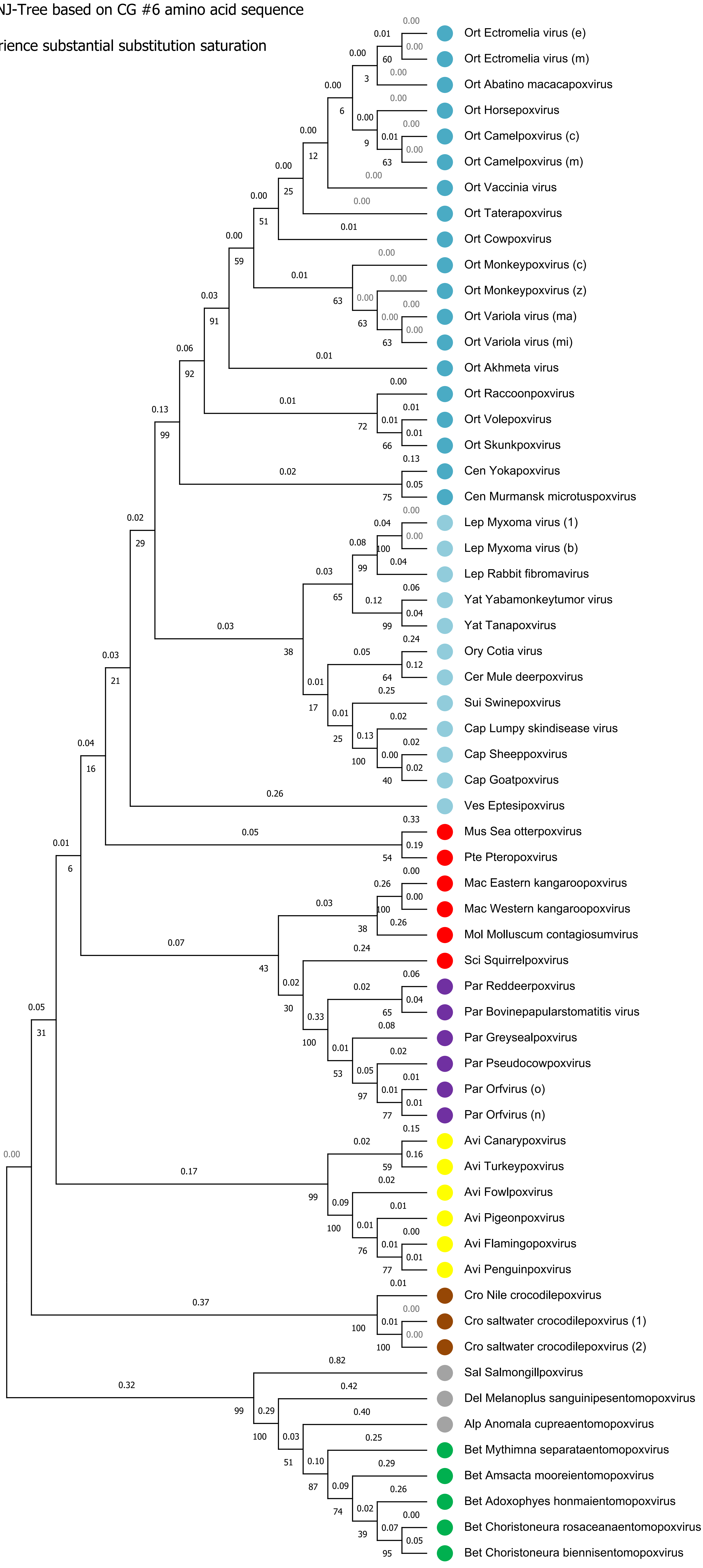

The NJ-Tree based on CG #7 amino acid sequence

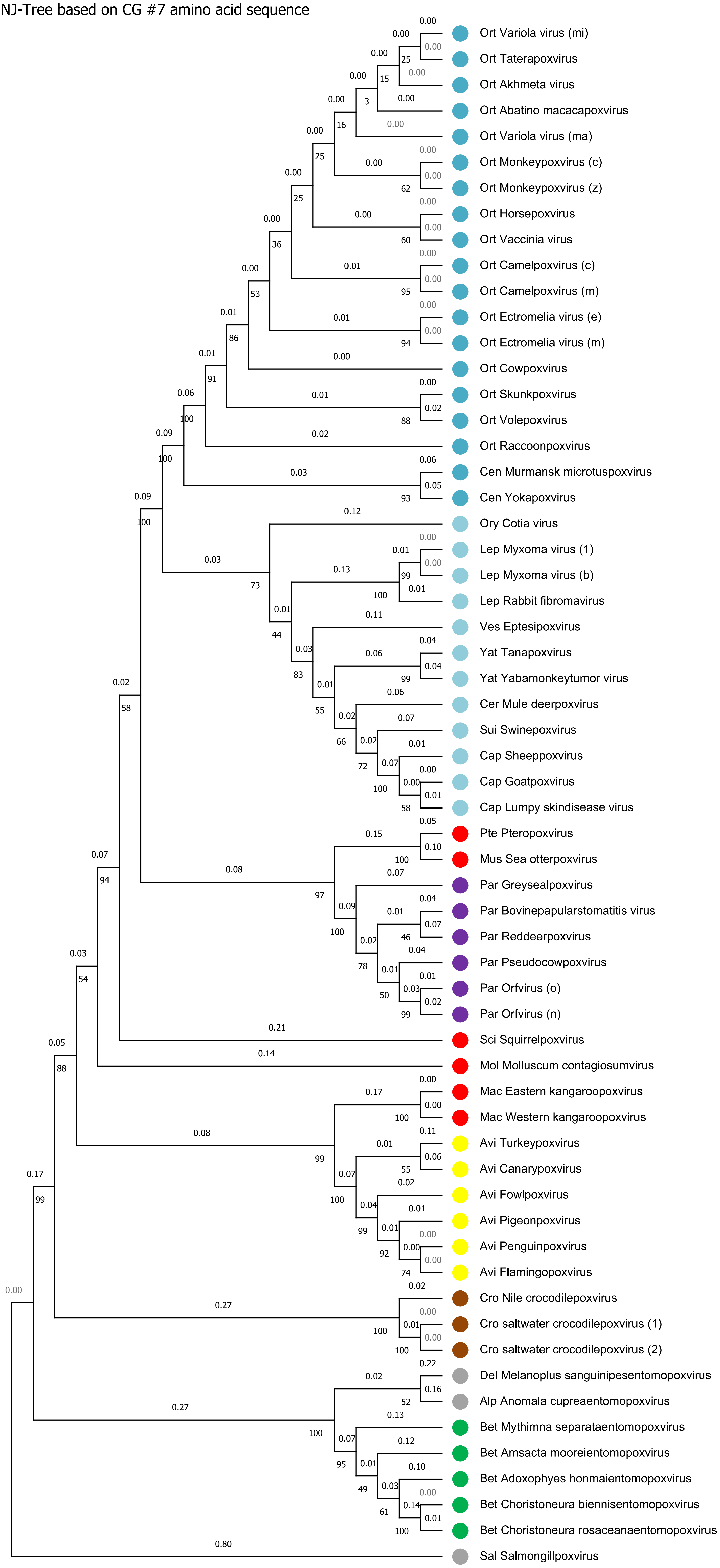

The NJ-Tree based on CG #8 amino acid sequence

Experience substantial substitution saturation

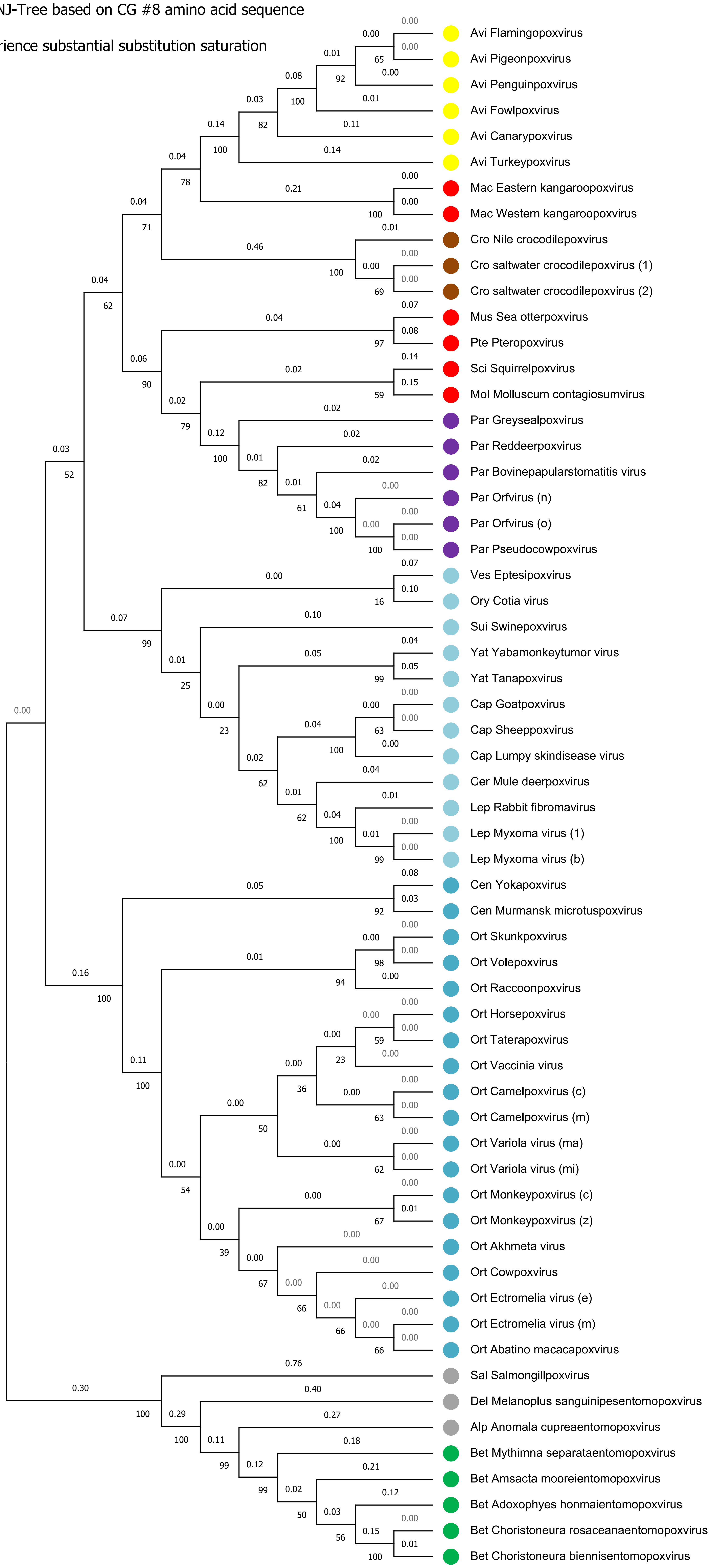

The NJ-Tree based on CG #9 amino acid sequence

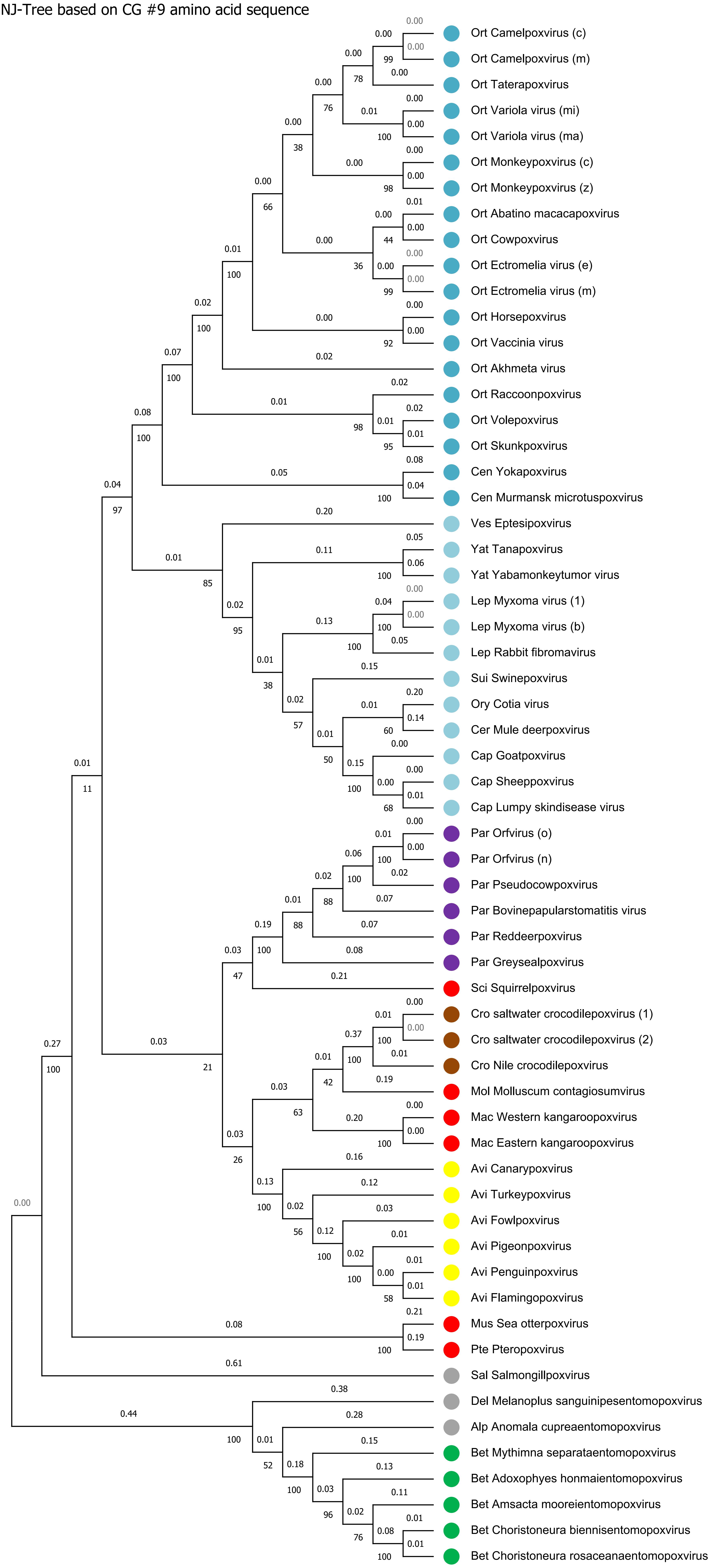

The NJ-Tree based on CG #10 amino acid sequence

Experience substantial substitution saturation

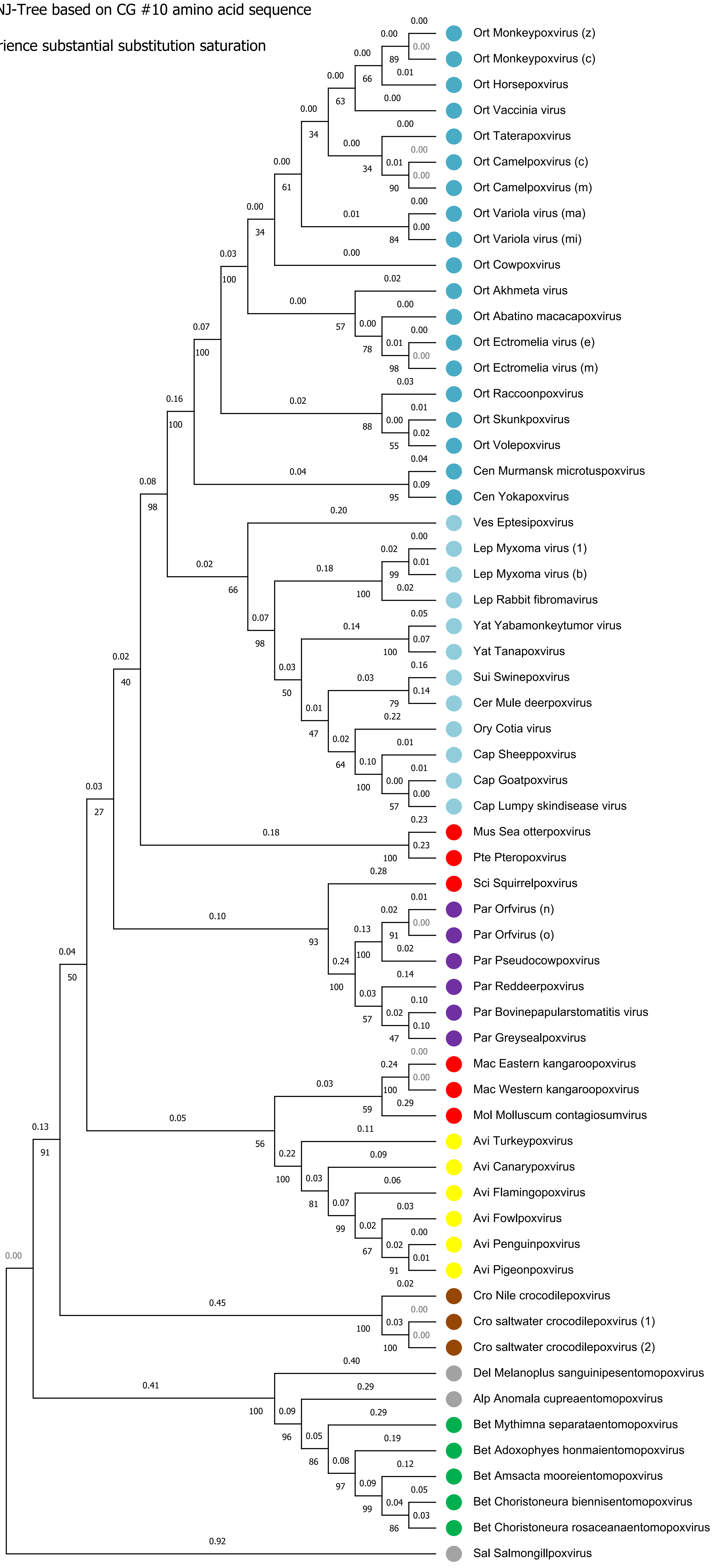

The NJ-Tree based on CG #11 amino acid sequence

Experience substantial substitution saturation

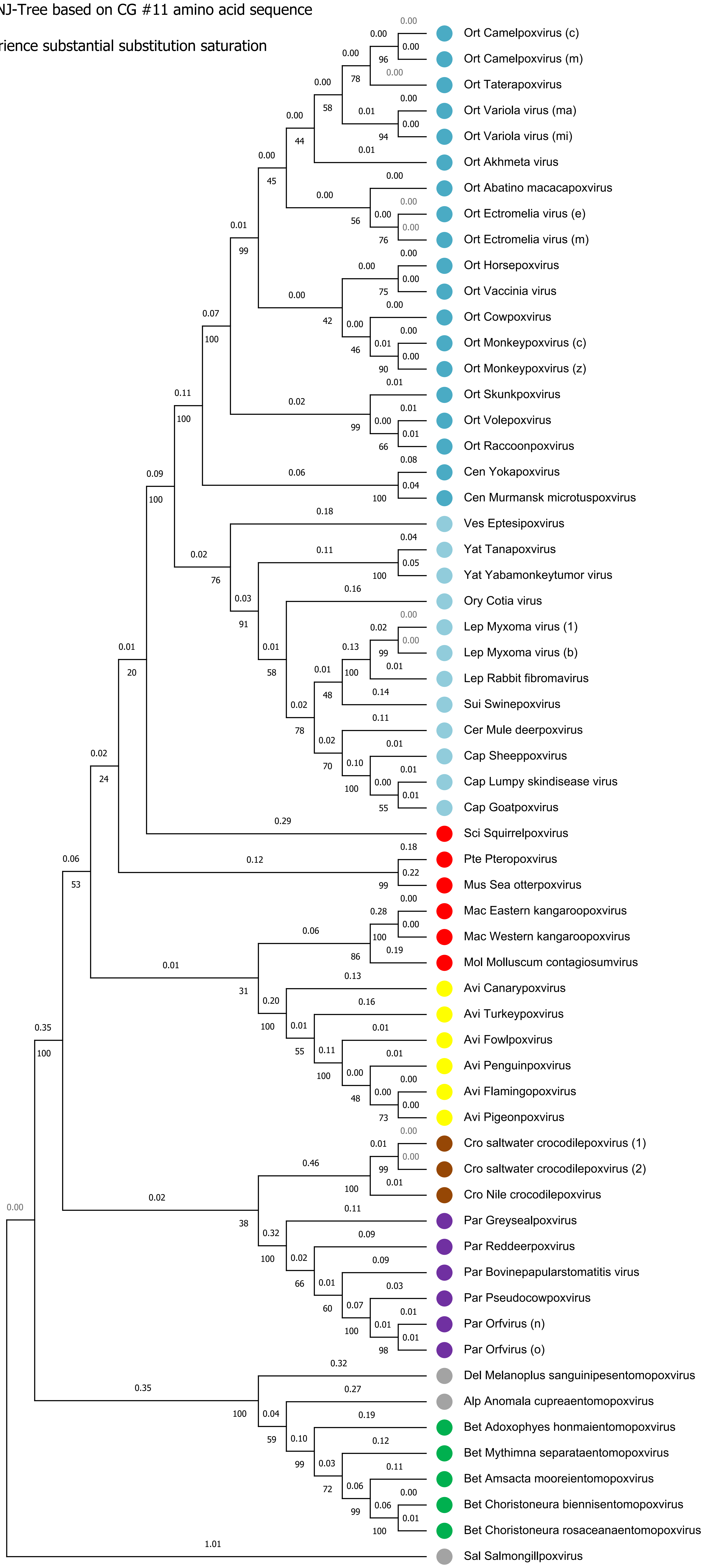

The NJ-Tree based on CG #12 amino acid sequence

Experience substantial substitution saturation

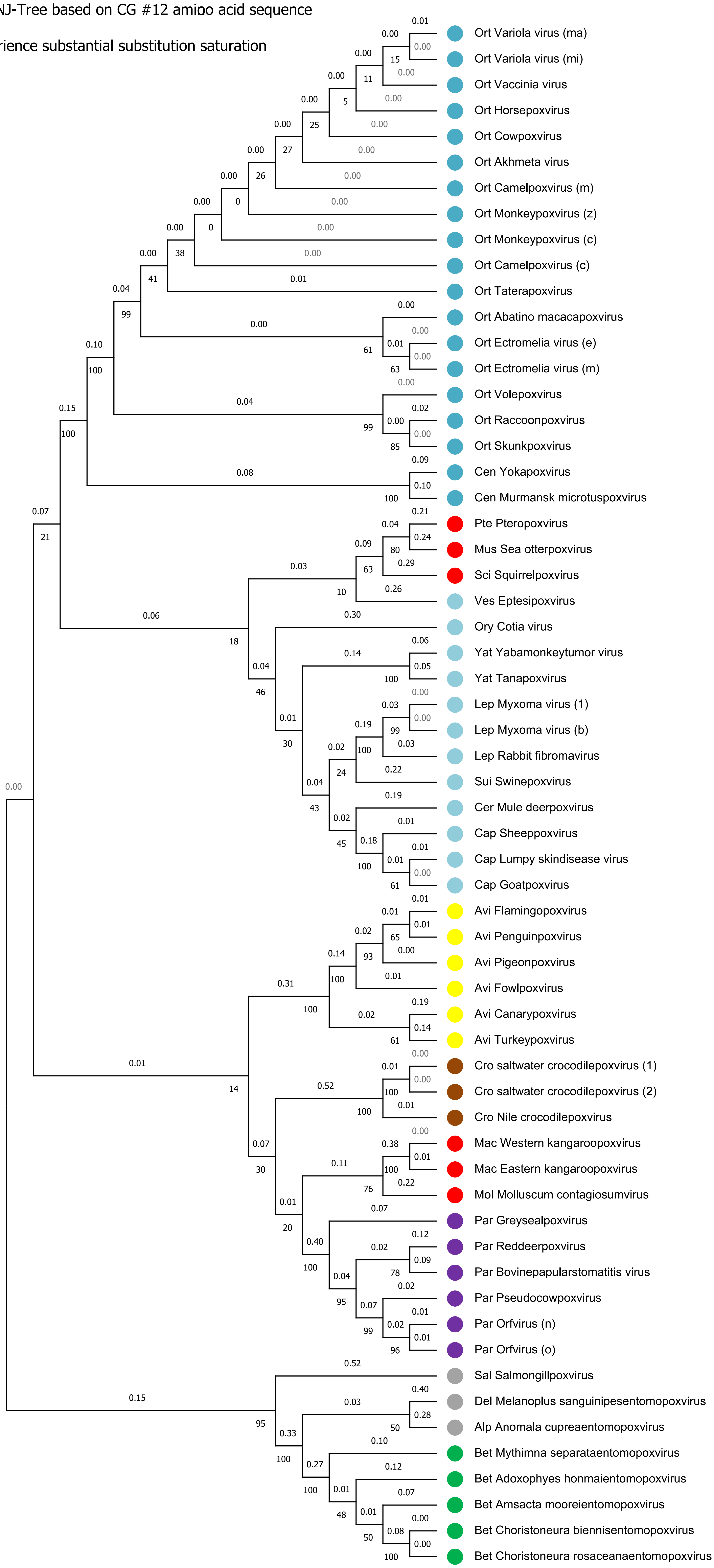

The NJ-Tree based on CG #13 amino acid sequence

Experience substantial substitution saturation

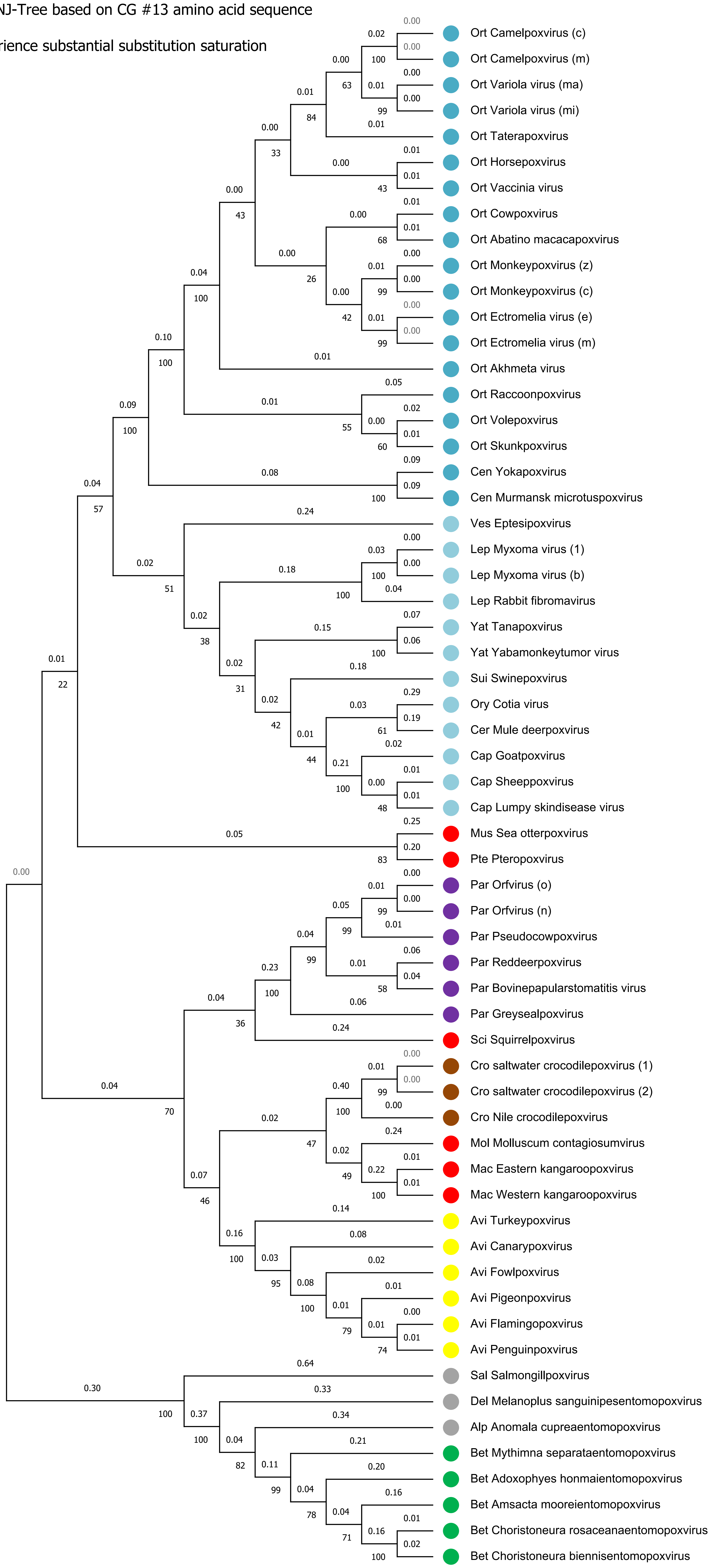

The NJ-Tree based on CG #14 amino acid sequence

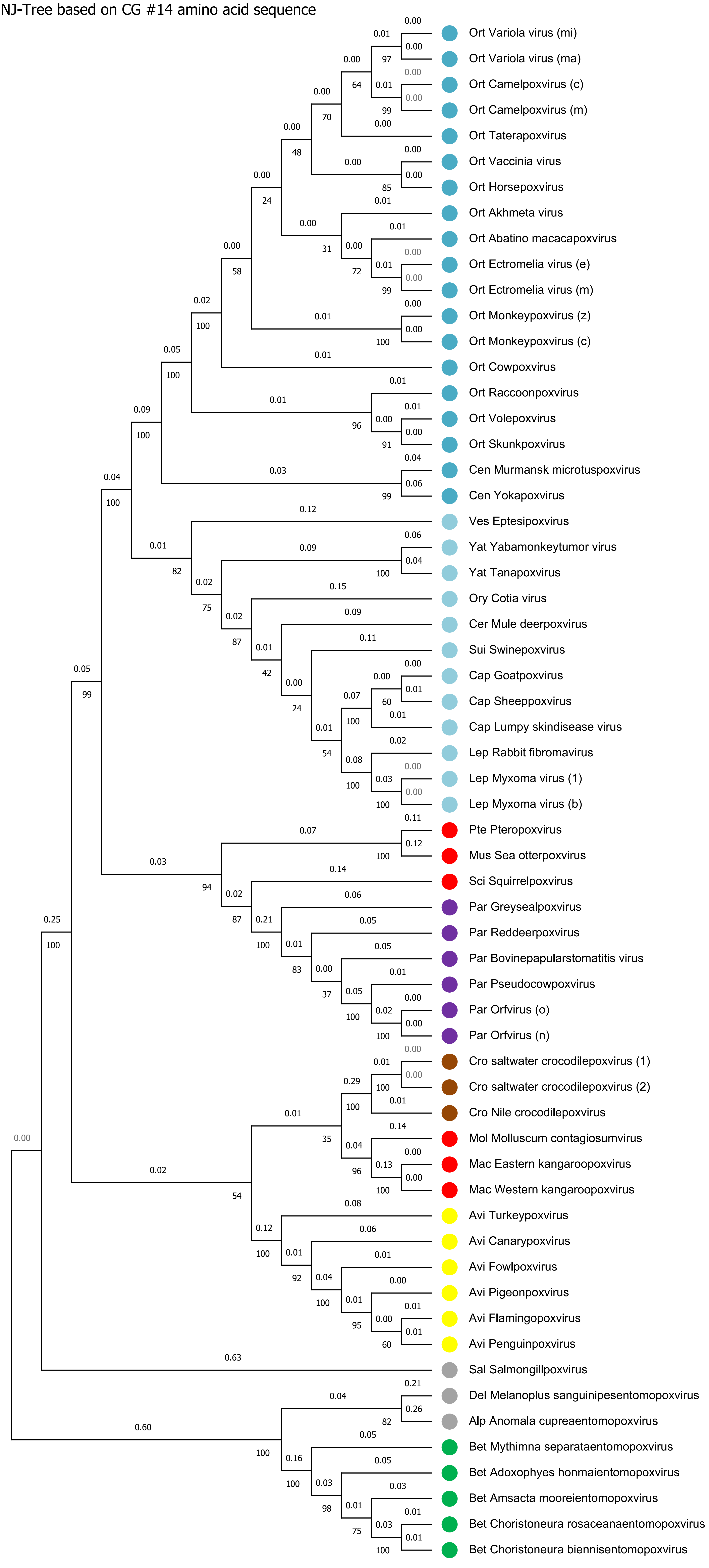

The NJ-Tree based on CG #15 amino acid sequence

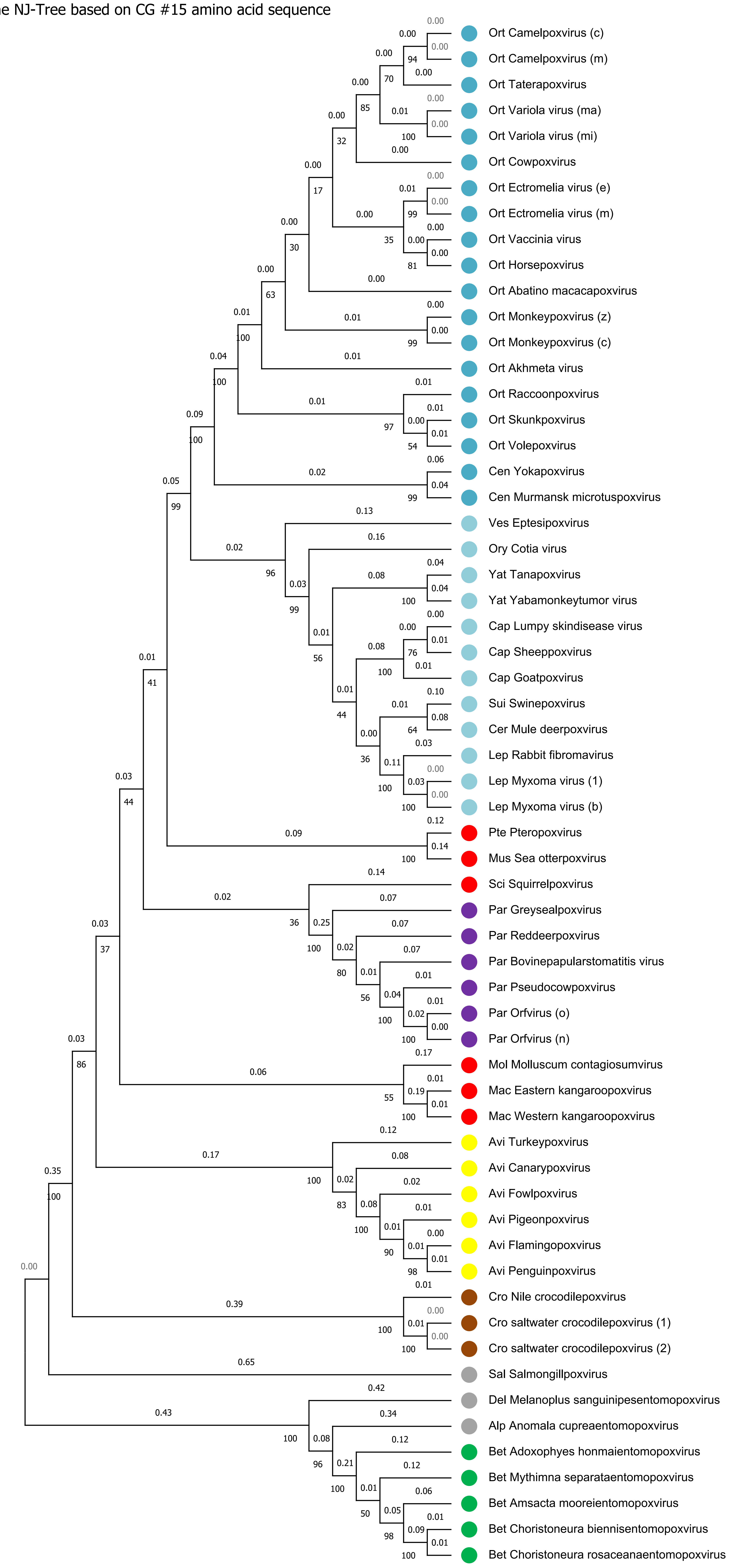

The NJ-Tree based on CG #16 amino acid sequence

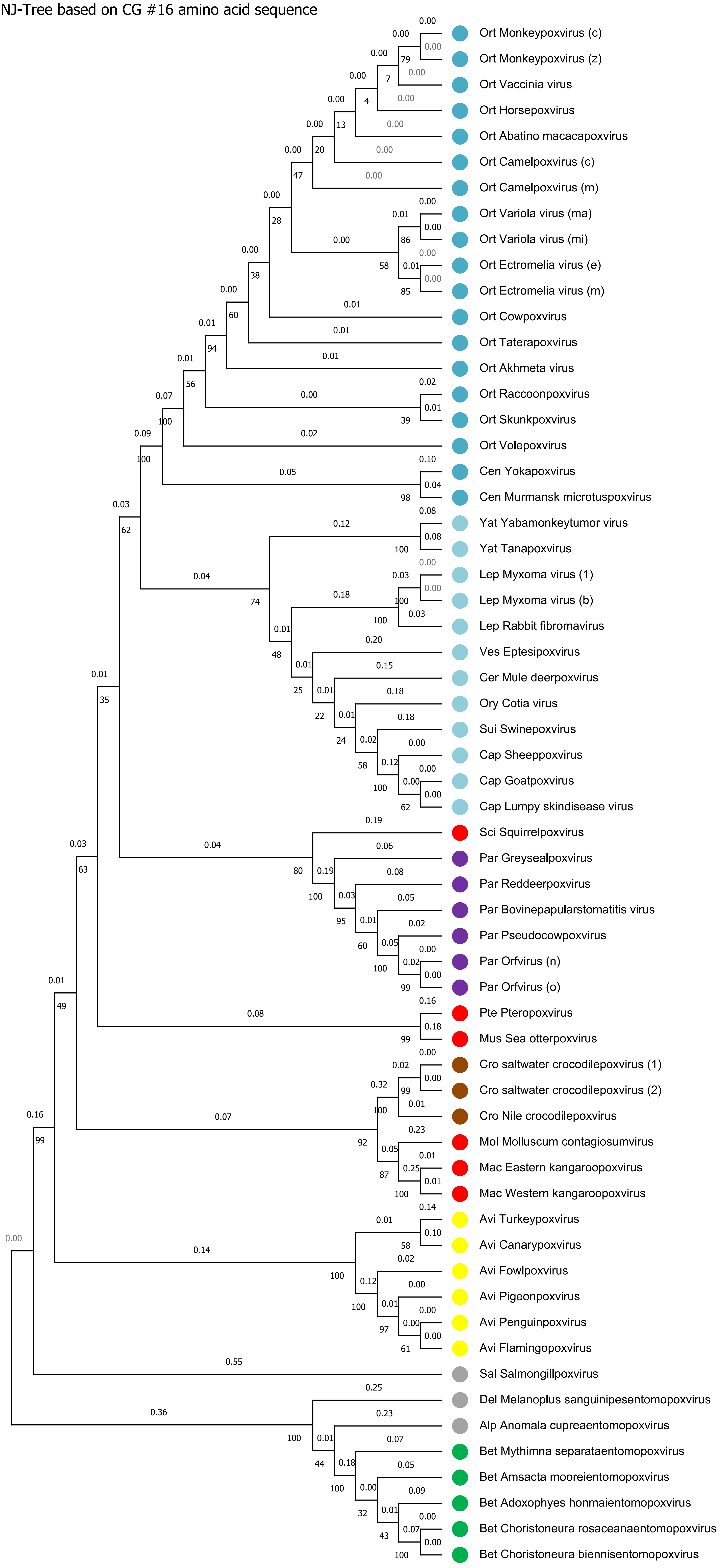

The NJ-Tree based on CG #17 amino acid sequence

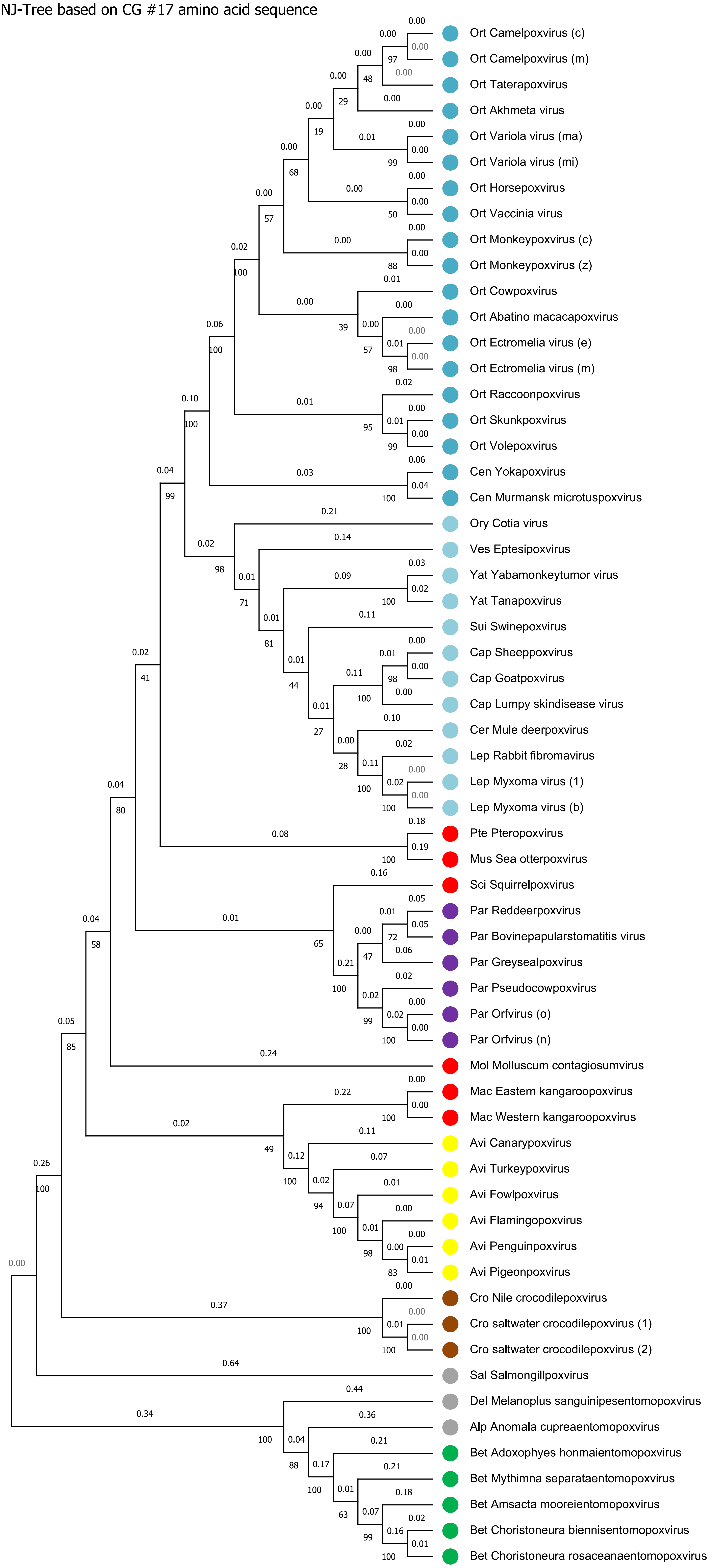

The NJ-Tree based on CG #18 amino acid sequence

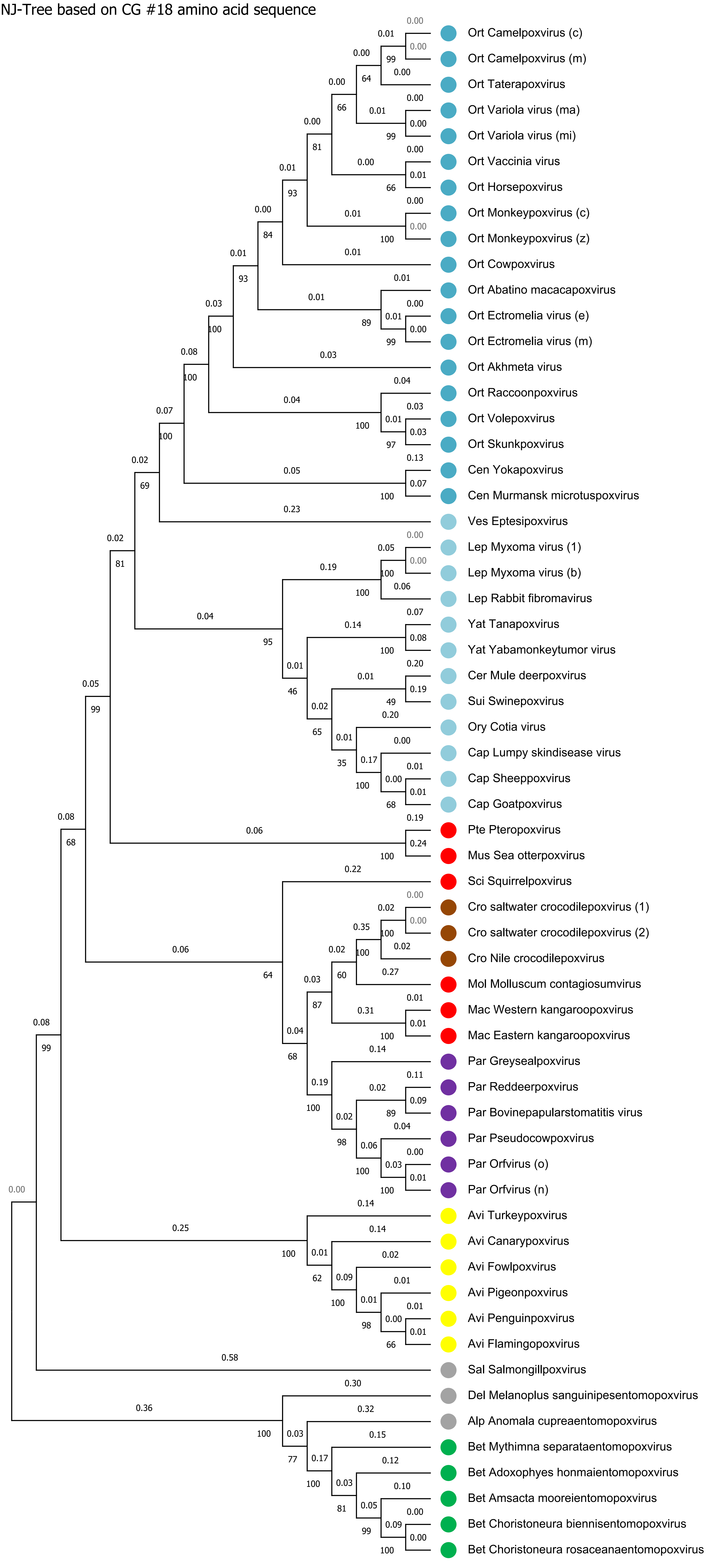

The NJ-Tree based on CG #19 amino acid sequence

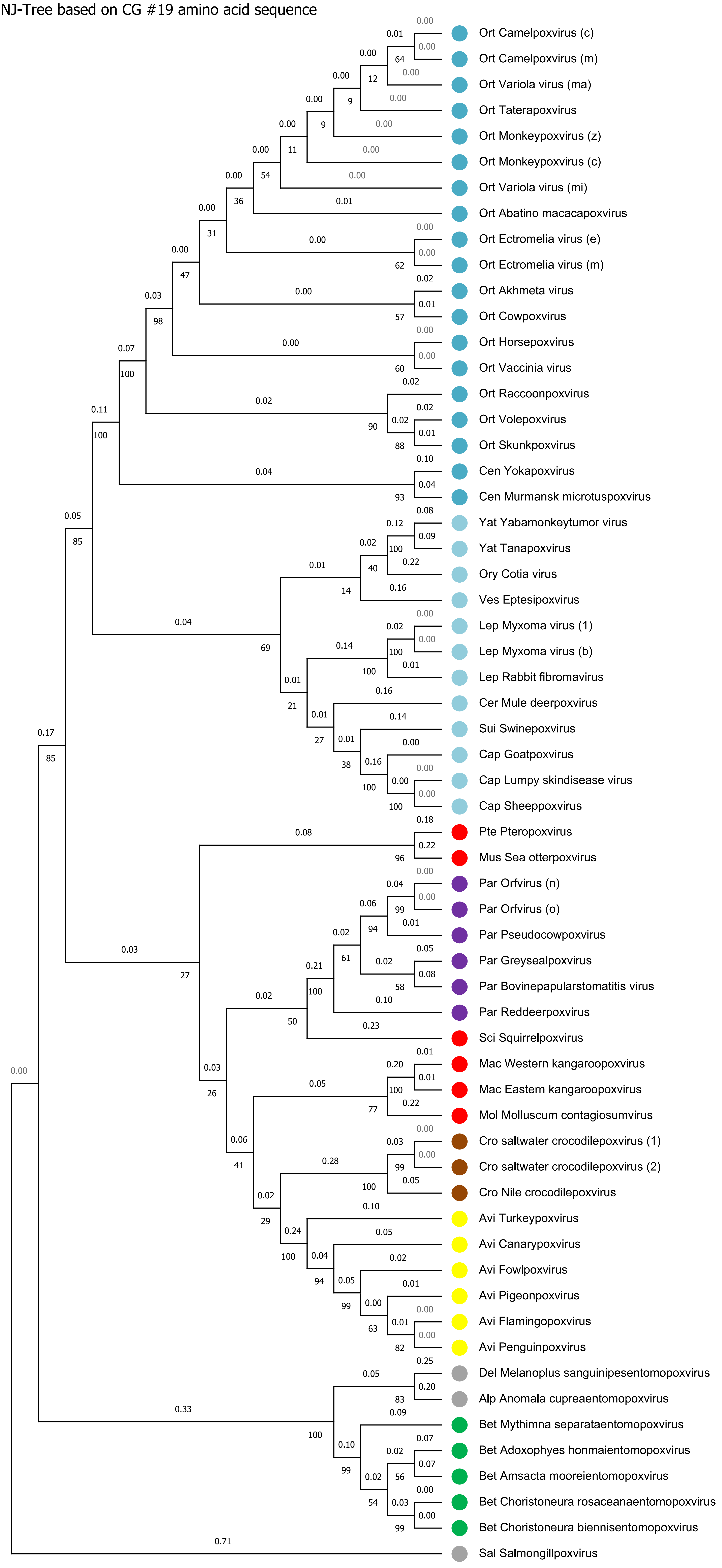

The NJ-Tree based on CG #20 amino acid sequence

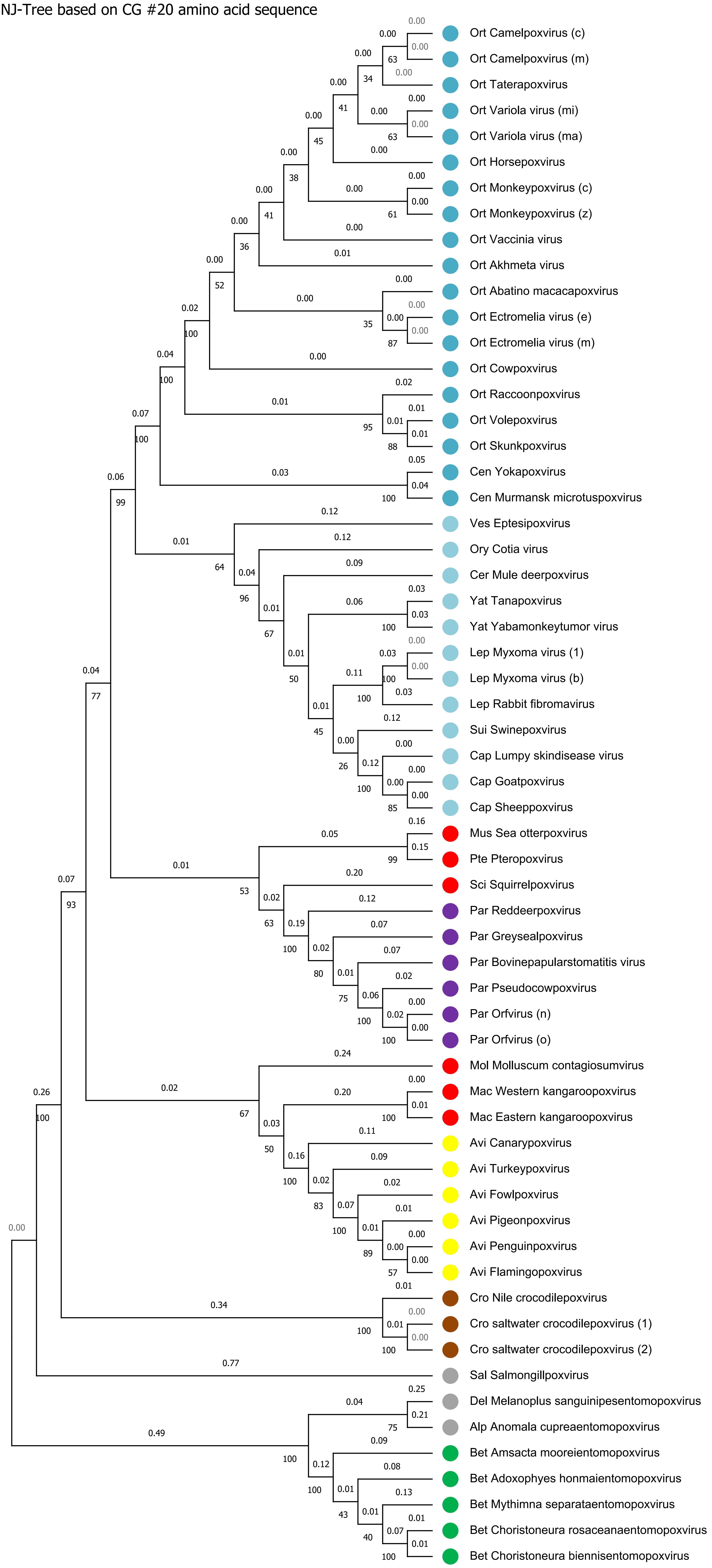

The NJ-Tree based on CG #21 amino acid sequence

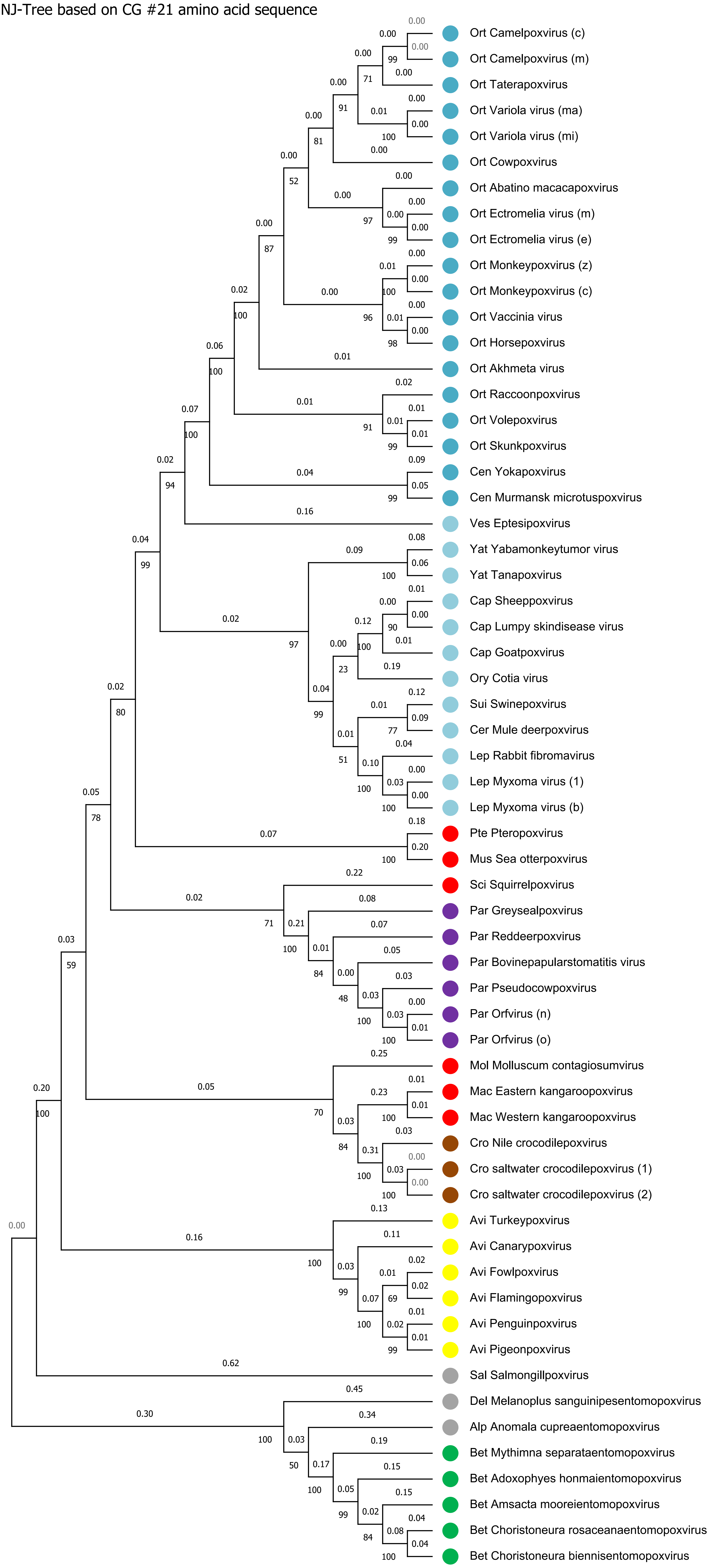

The NJ-Tree based on CG #22 amino acid sequence

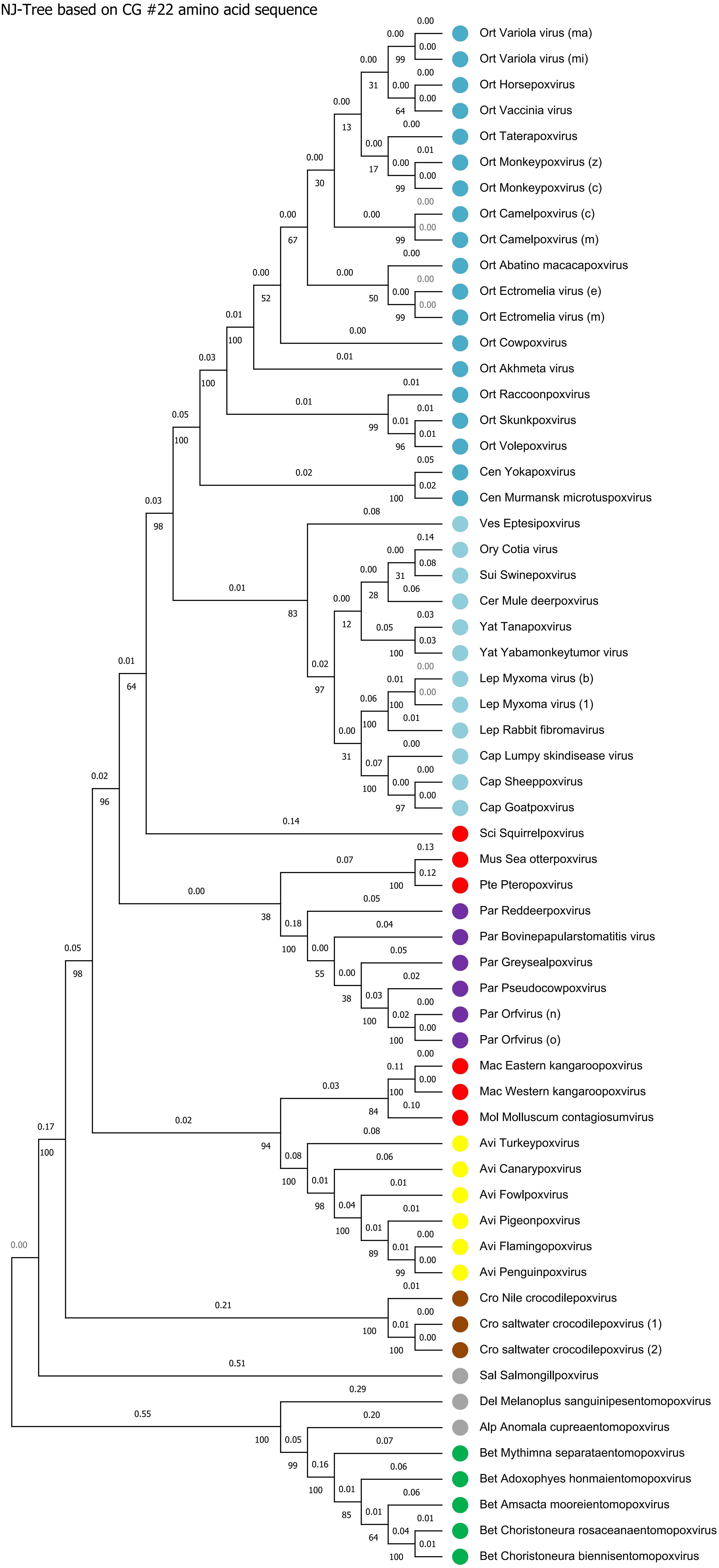

Supplement: Supplementary data 7 [file mmc7.pdf]
