# Supplementary material for: Genomic analysis of Poxviridae and exploring qualified gene sequences for phylogenetics
Source: Comput Struct Biotechnol J. 2021 Sep 28;19:5479–86. doi: 10.1016/j.csbj.2021.09.031 (PMC8515299; doi:10.1016/j.csbj.2021.09.031)

Supplementary file 4: The ML-Tree based on single amino acid sequence of core genes. The numbers on the branches represent branch lengths/genetic distances and numbers below the branch points represent bootstrap values. The color of the branch endpoints represents the classification results based on synteny analysis.

Legend

- **Group *Ch*-A1**
- **Group *Ch*-A2**
- **Group *Ch*-B**
- **Group *Ch*-C**
- **Group *Ch*-D**
- **Group *Ch*-X**
- **Group *En-A***
- **NA**

The ML-Tree based on CG #1 amino acid sequence

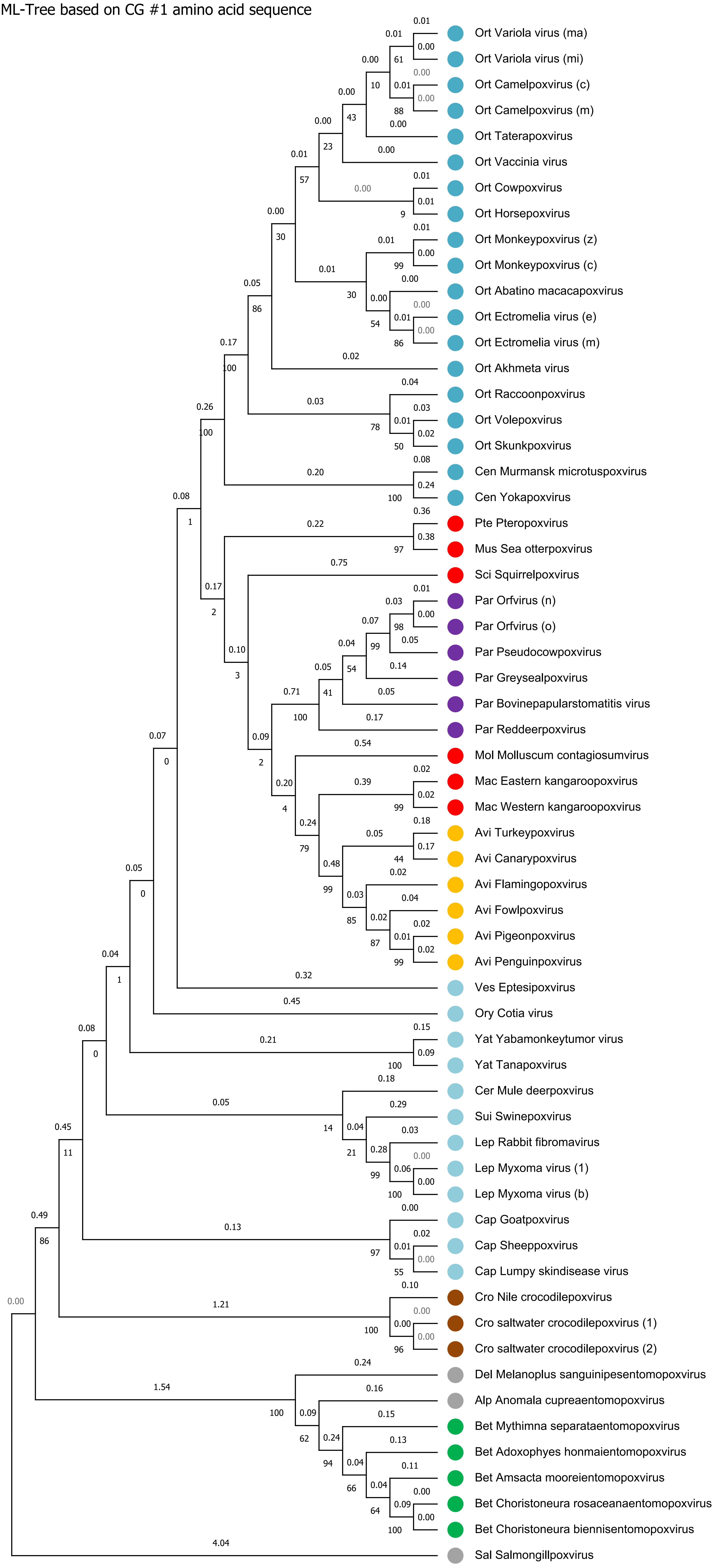

The ML-Tree based on CG #2 amino acid sequence

Experience substantial substitution saturation

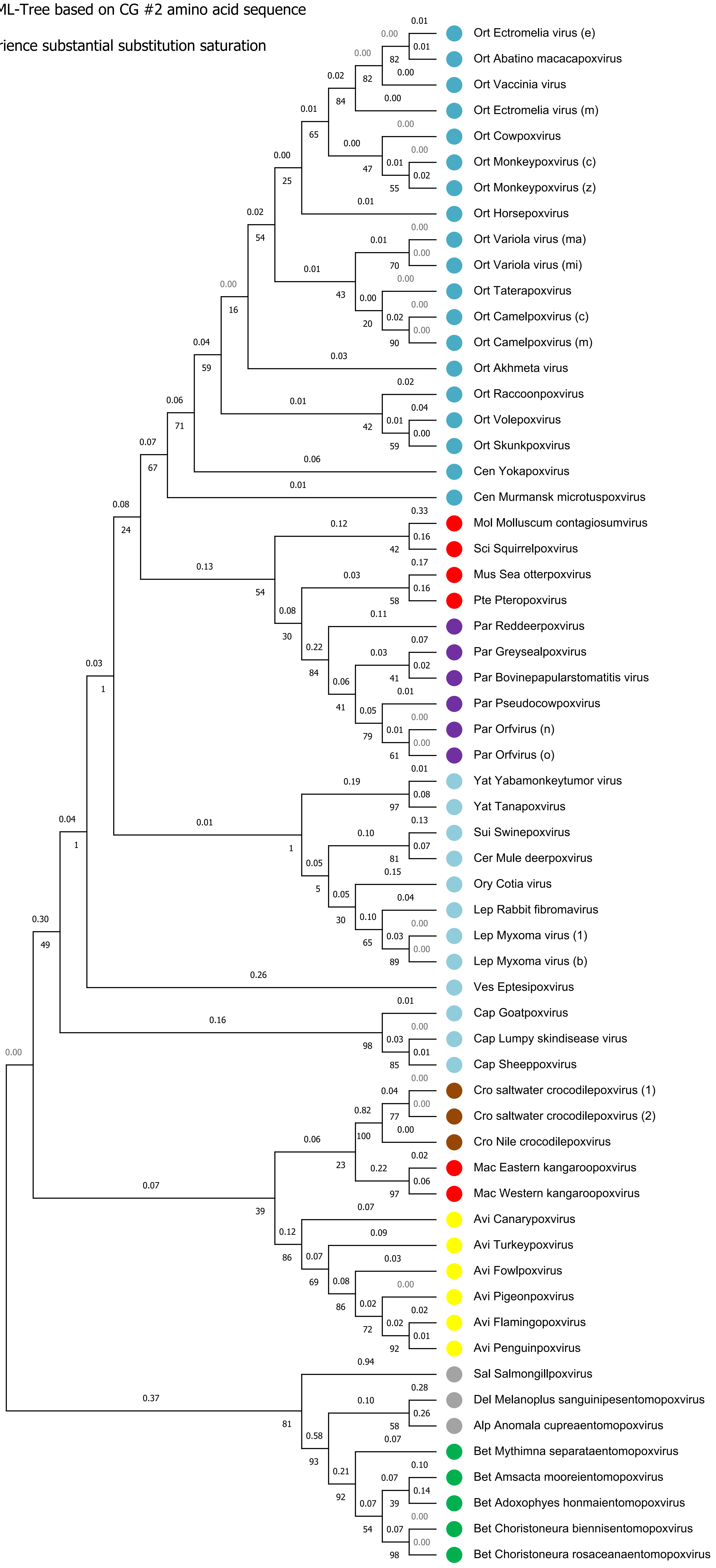

The ML-Tree based on CG #3 amino acid sequence

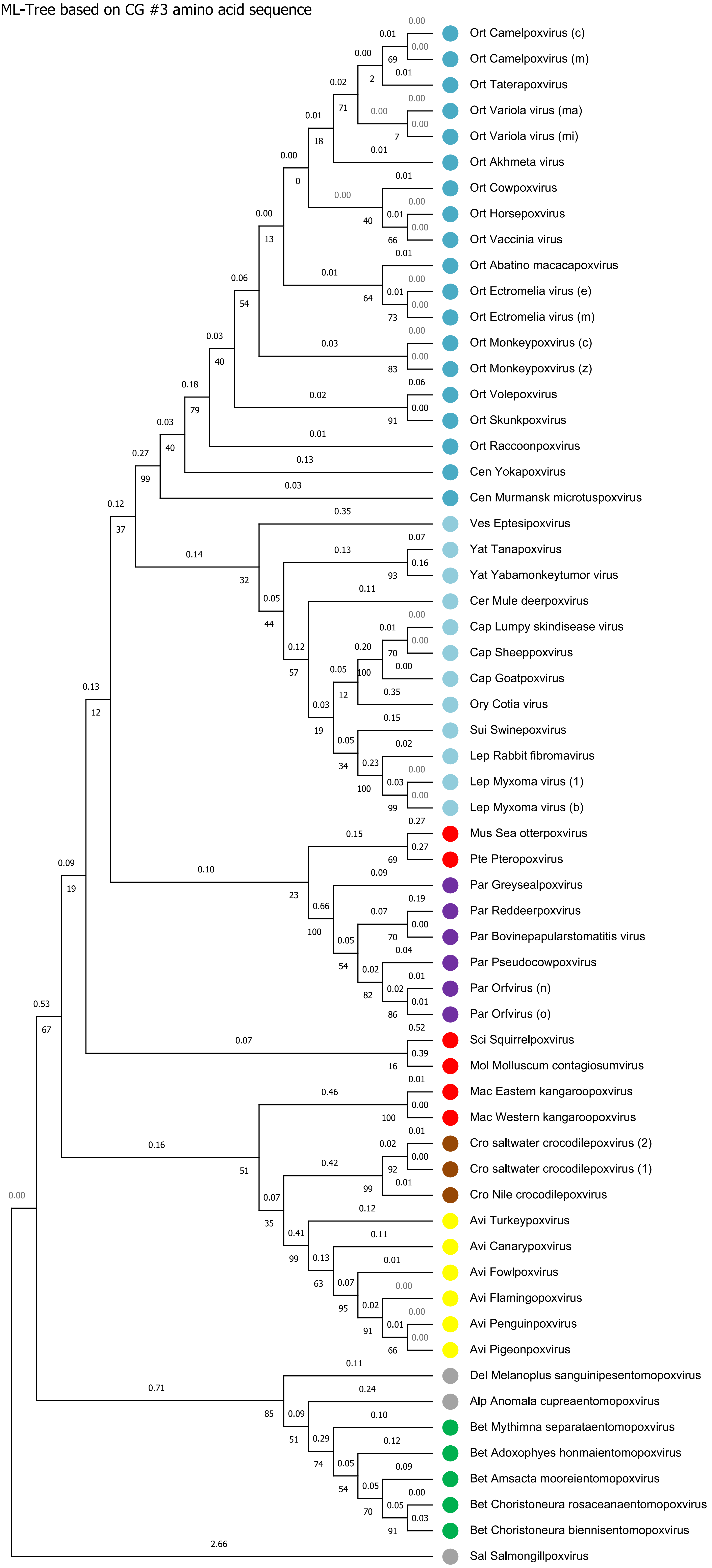

The ML-Tree based on CG #4 amino acid sequence

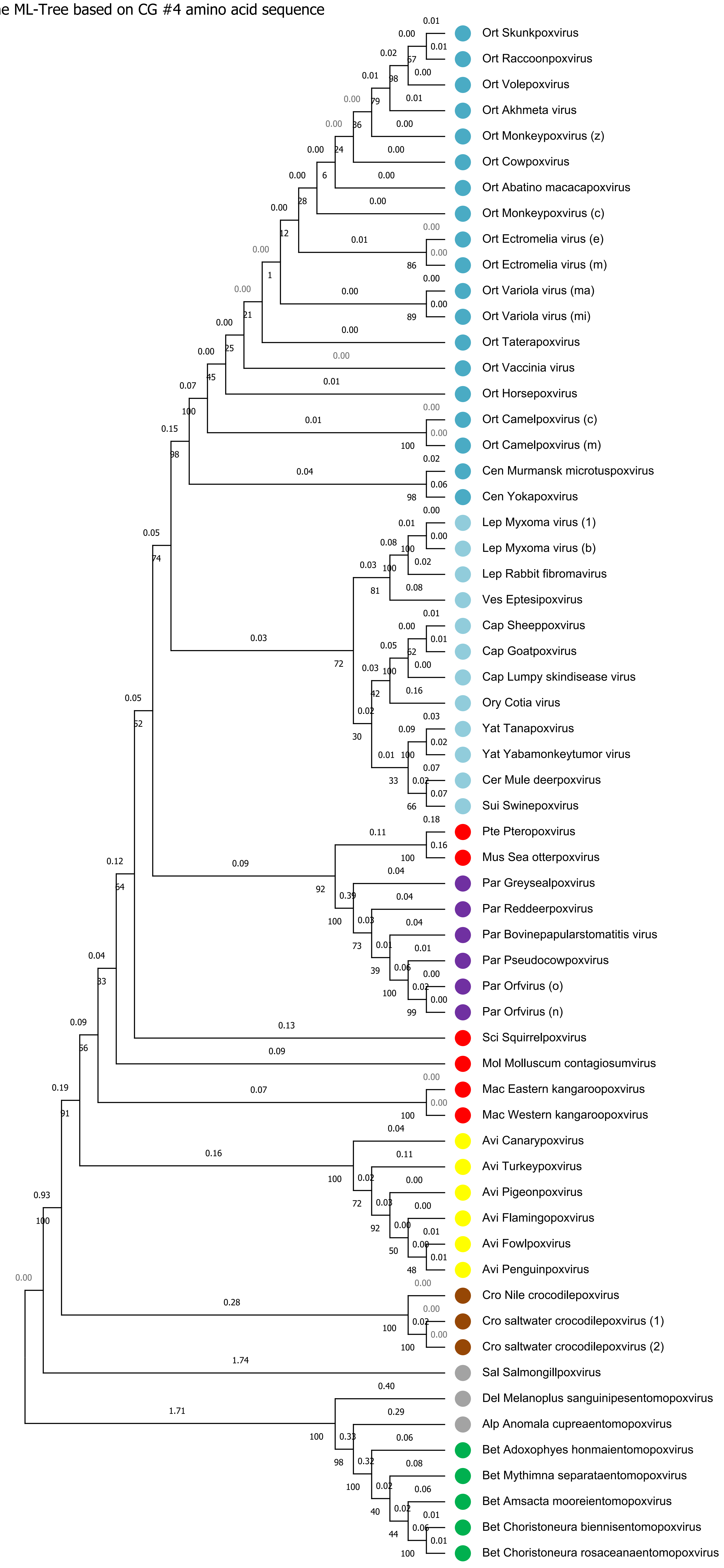

The ML-Tree based on CG #5 amino acid sequence

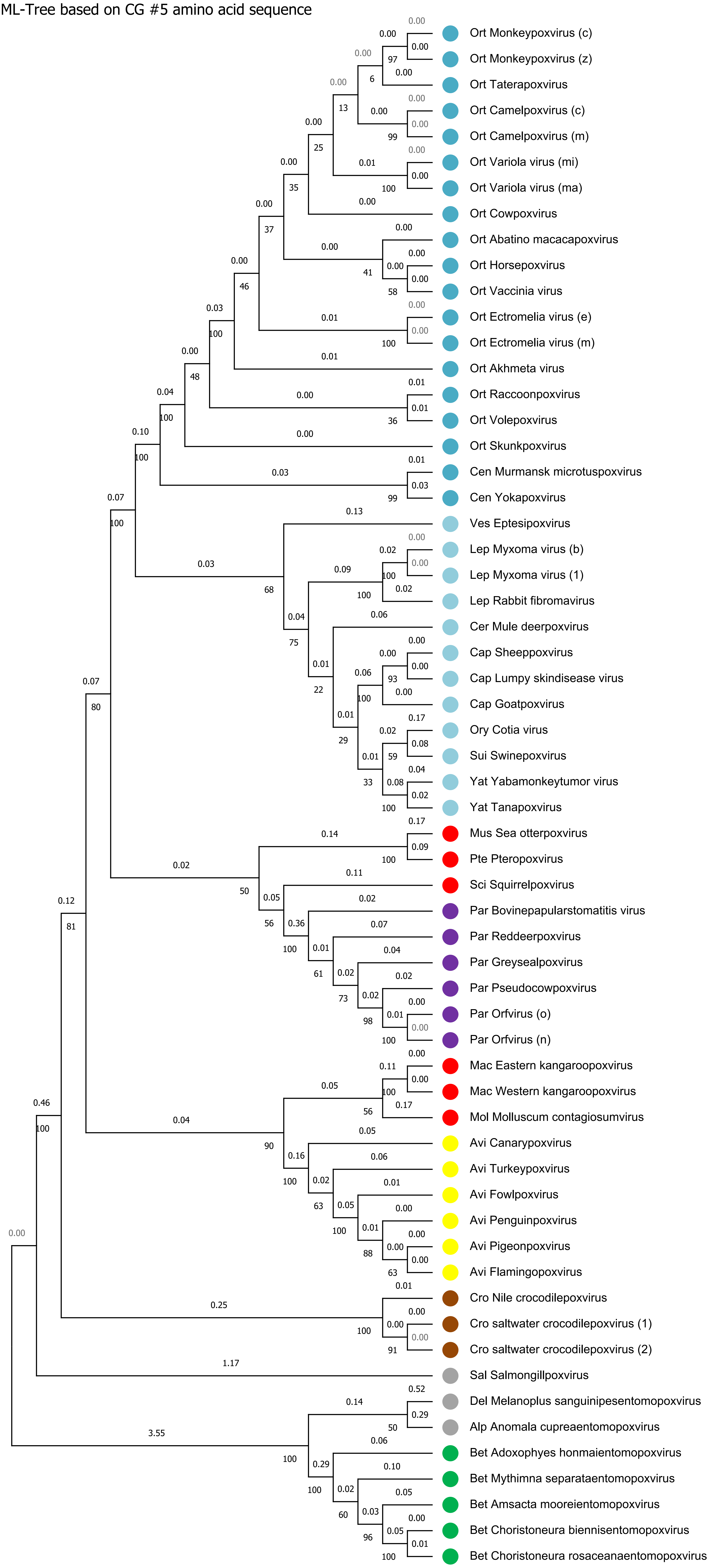

The ML-Tree based on CG #6 amino acid sequence

Experience substantial substitution saturation

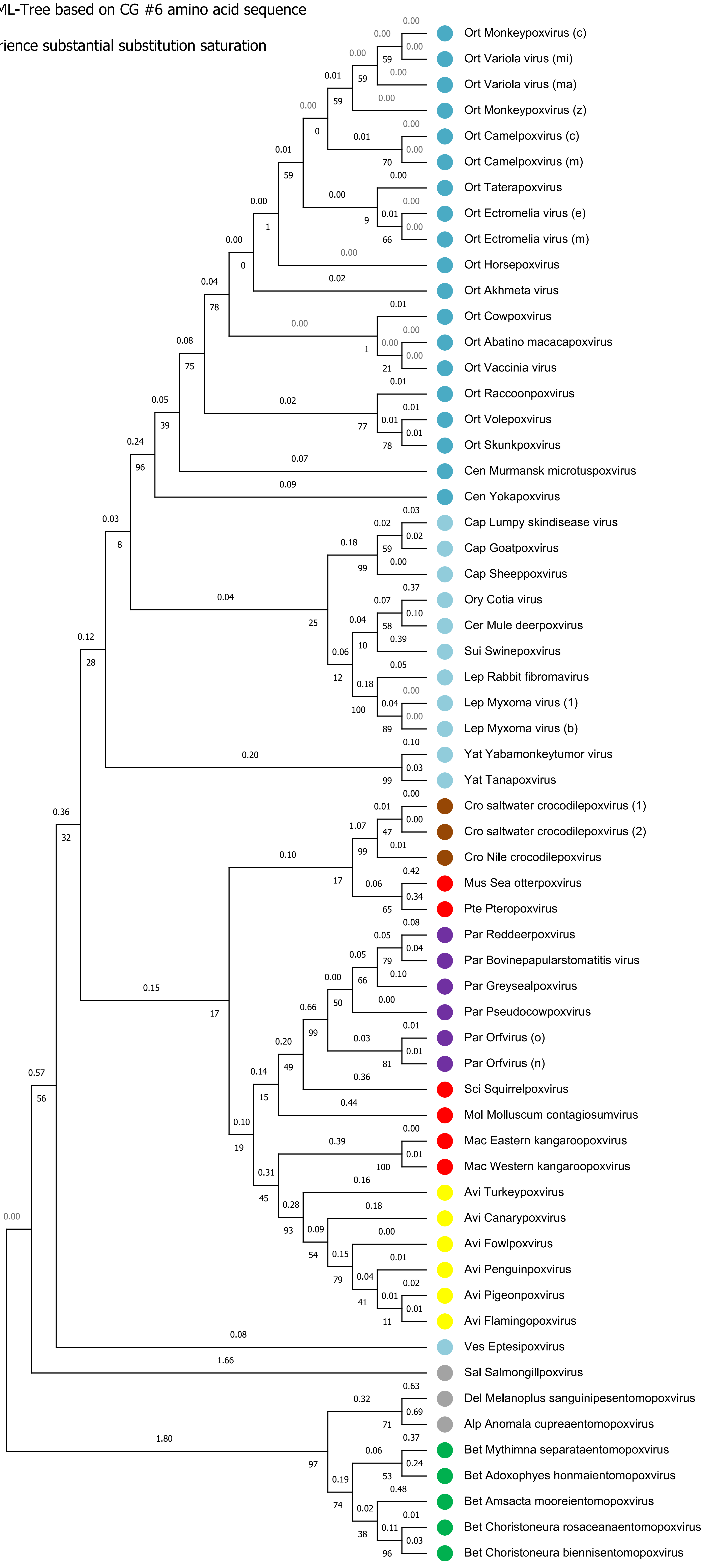

The ML-Tree based on CG #7 amino acid sequence

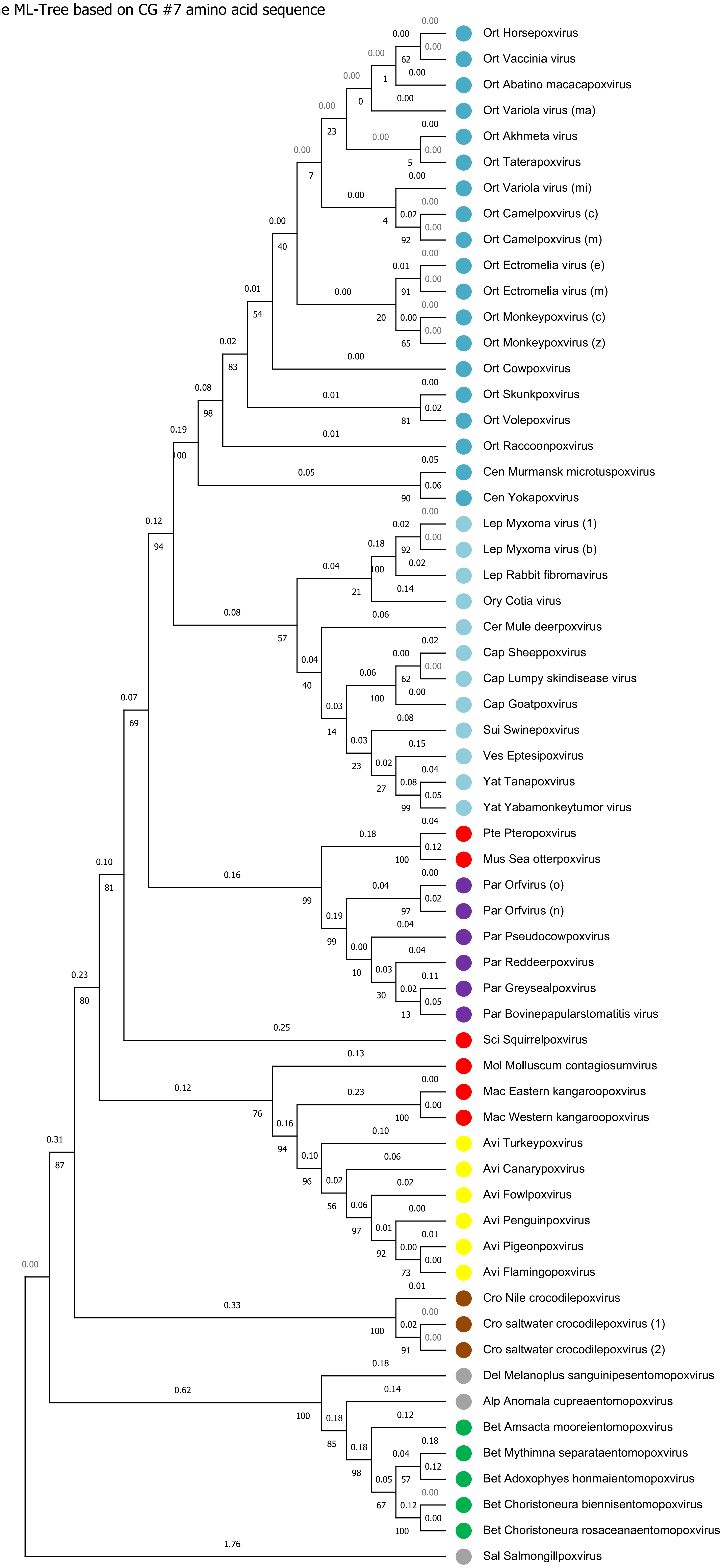

The ML-Tree based on CG #8 amino acid sequence

Experience substantial substitution saturation

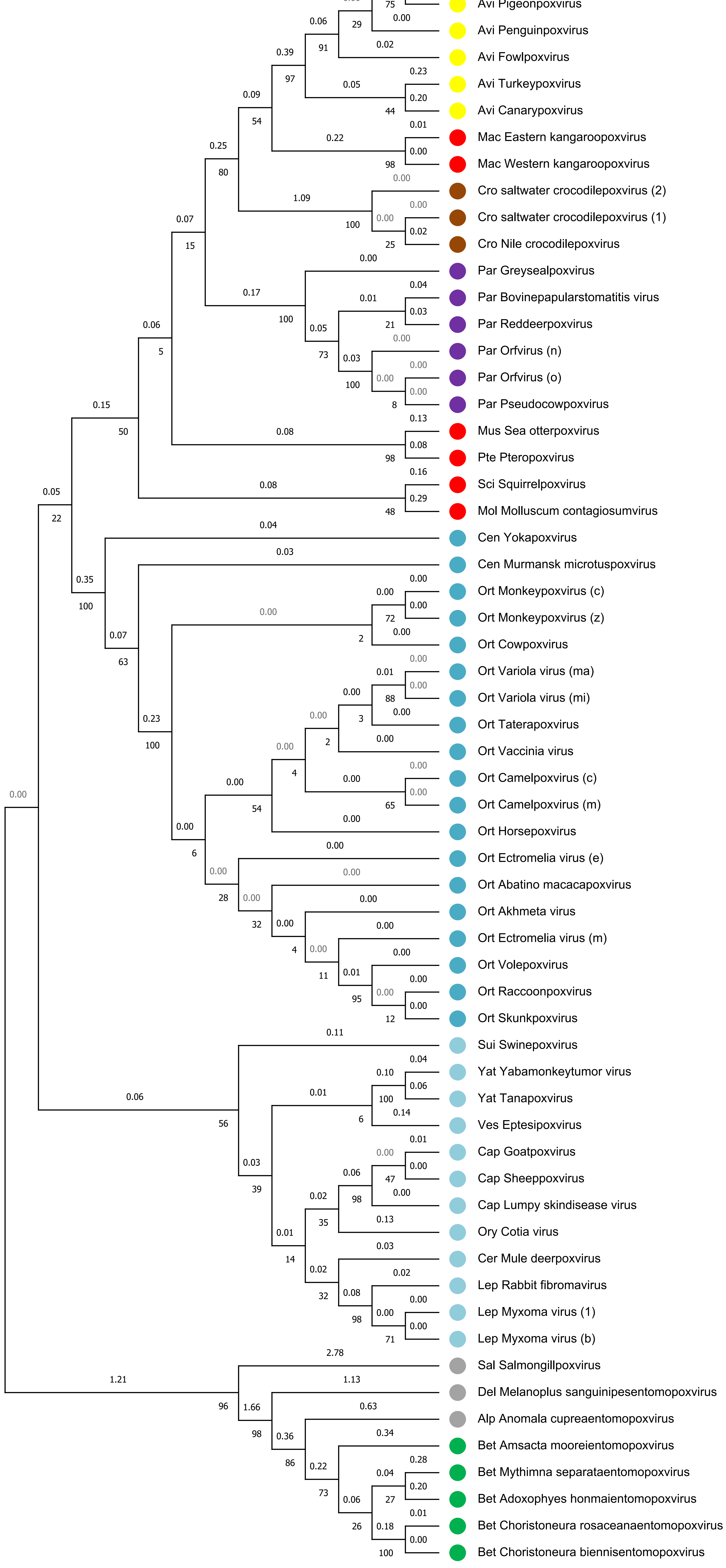

The ML-Tree based on CG #9 amino acid sequence

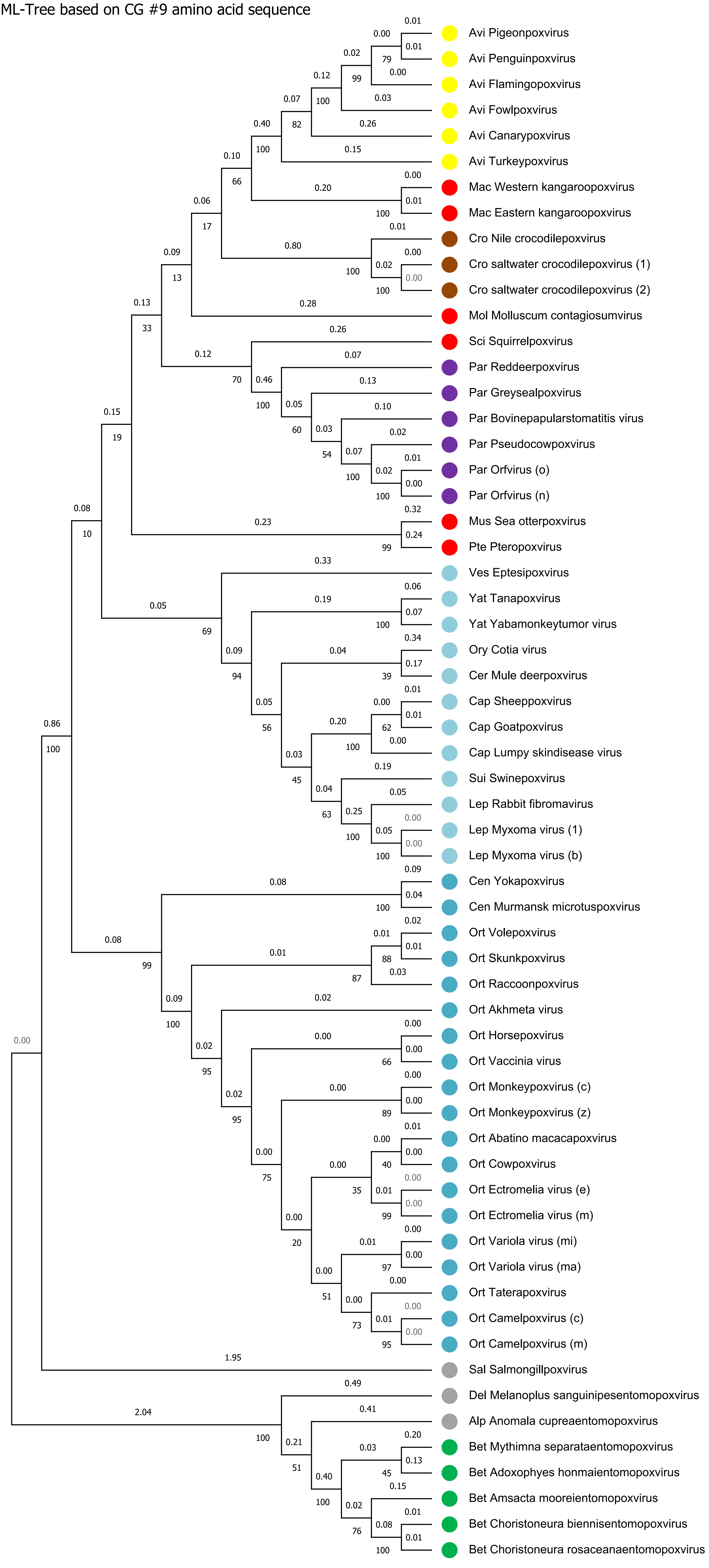

The ML-Tree based on CG #10 amino acid sequence

Experience substantial substitution saturation

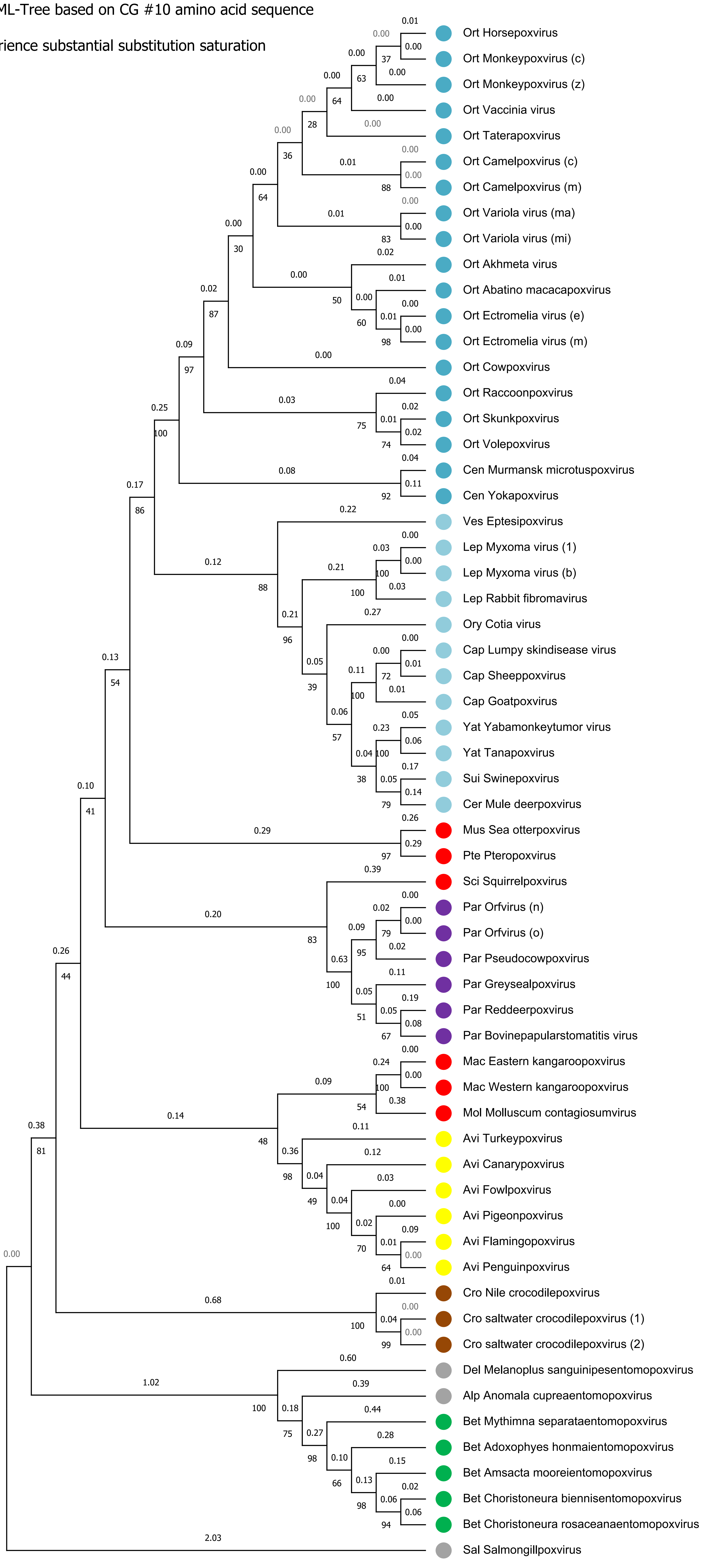

The ML-Tree based on CG #11 amino acid sequence

Experience substantial substitution saturation

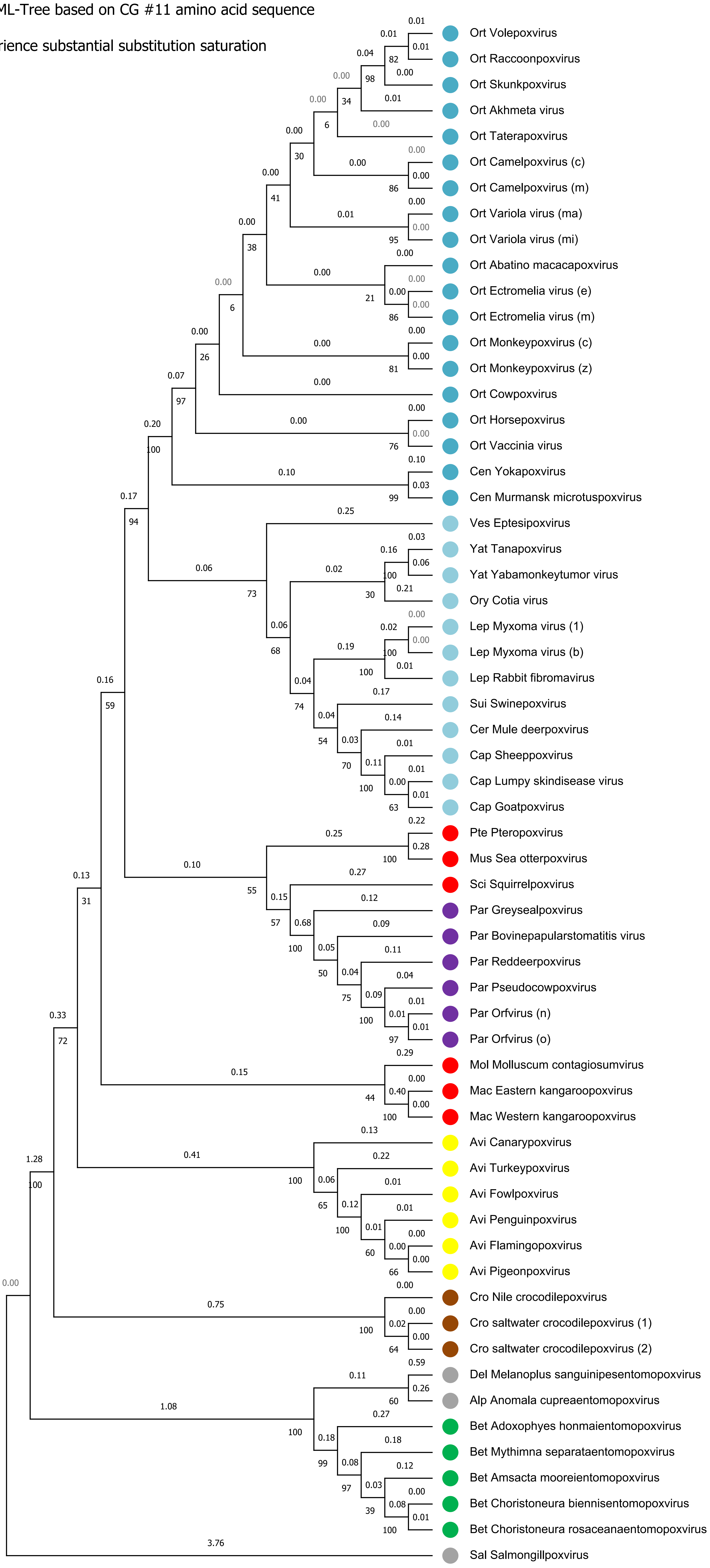

The ML-Tree based on CG #12 amino acid sequence

Experience substantial substitution saturation

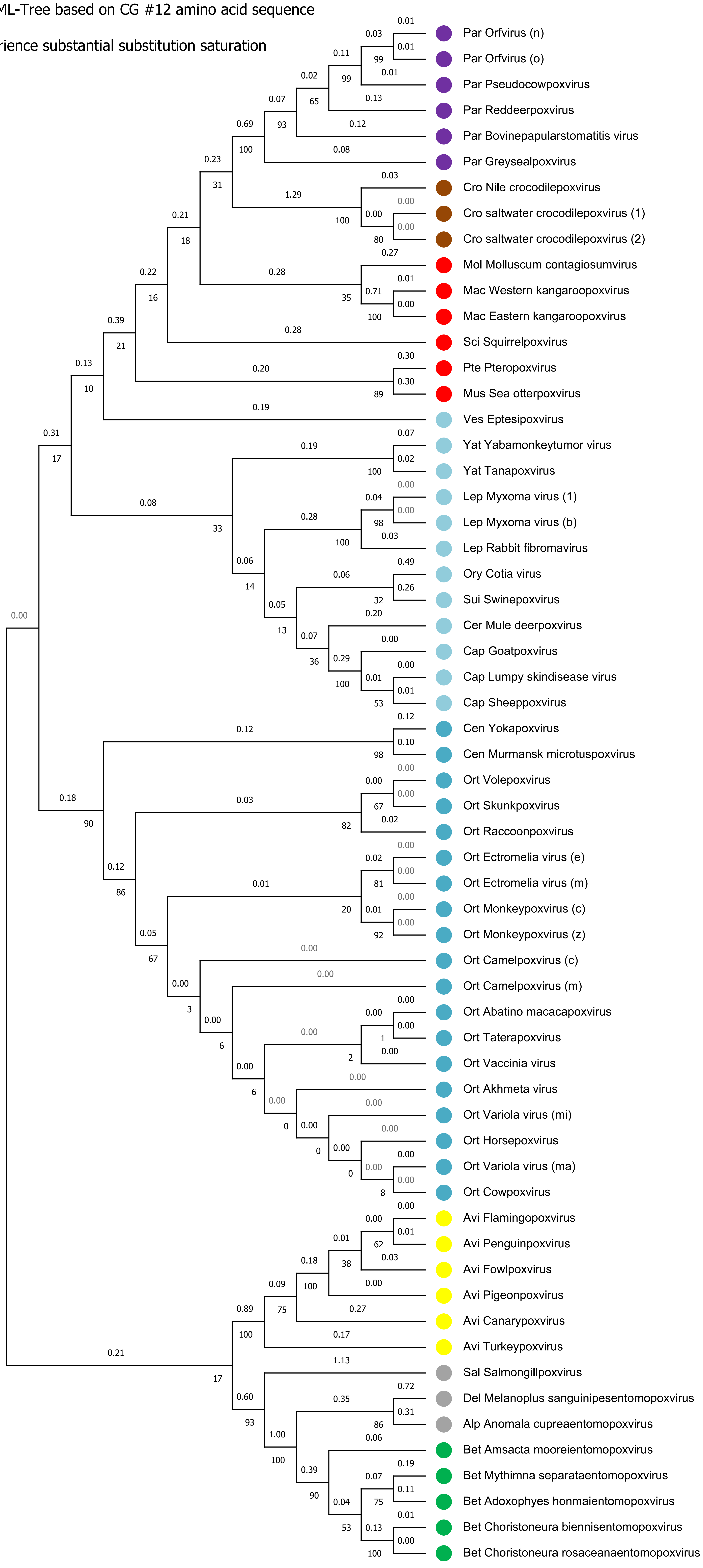

The ML-Tree based on CG #13 amino acid sequence

Experience substantial substitution saturation

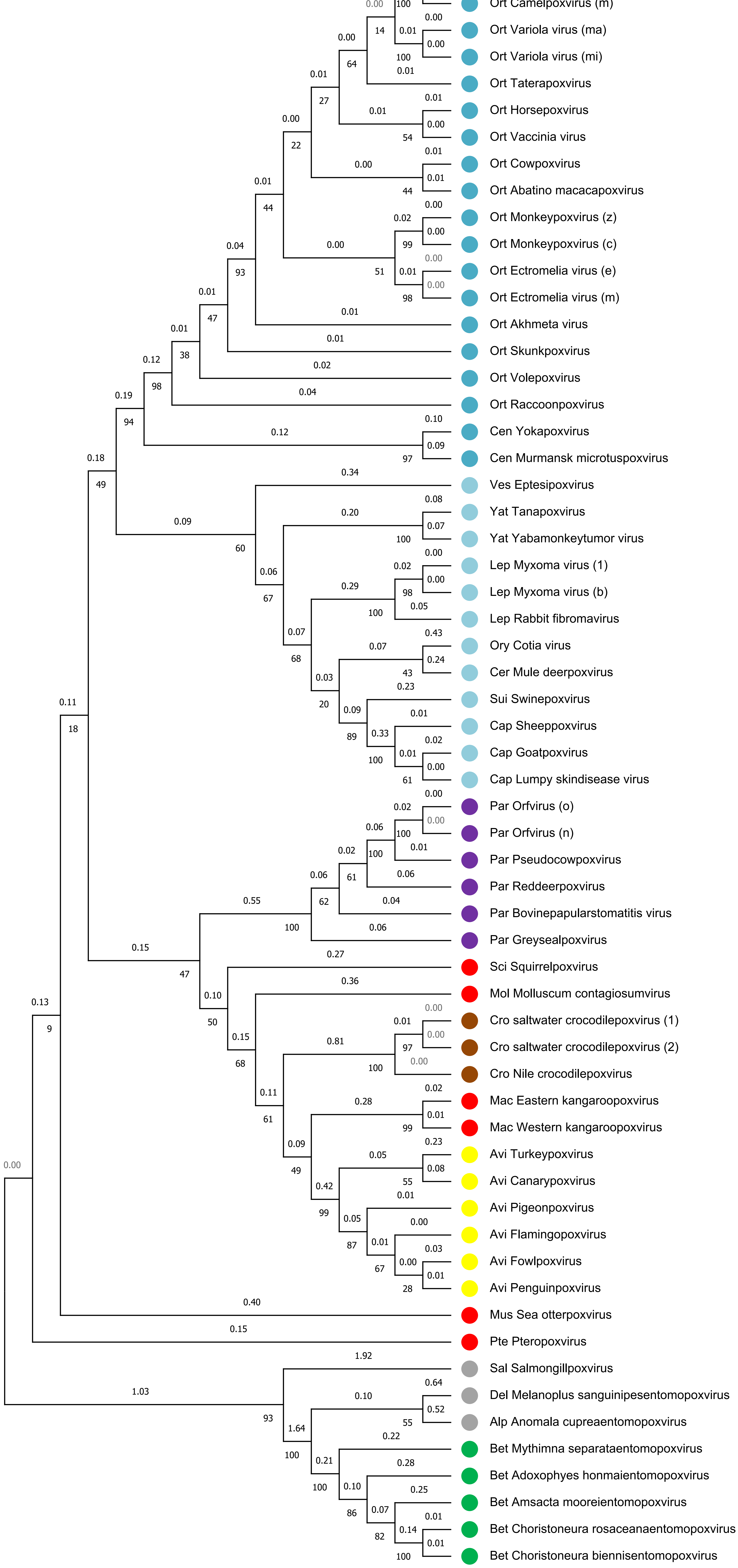

The ML-Tree based on CG #14 amino acid sequence

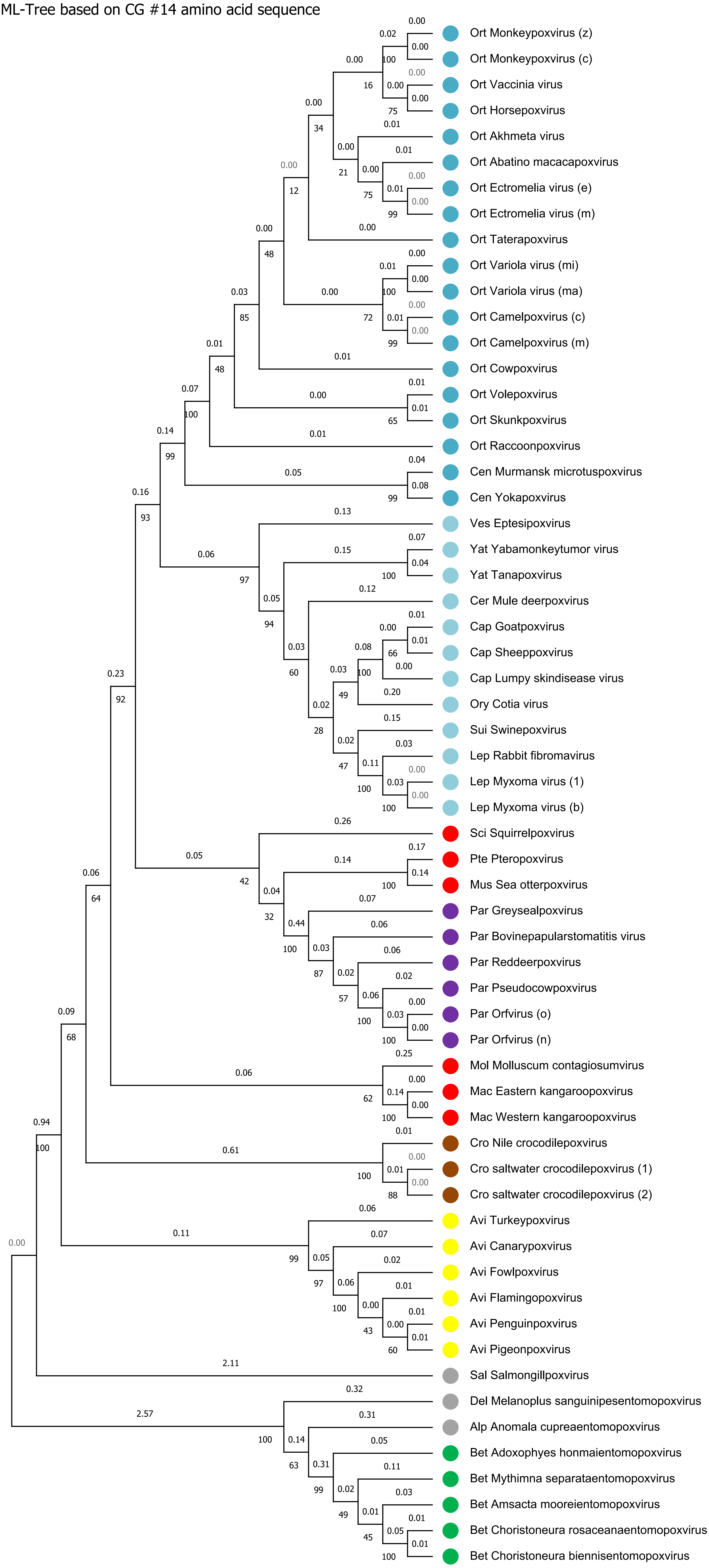

The ML-Tree based on CG #15 amino acid sequence

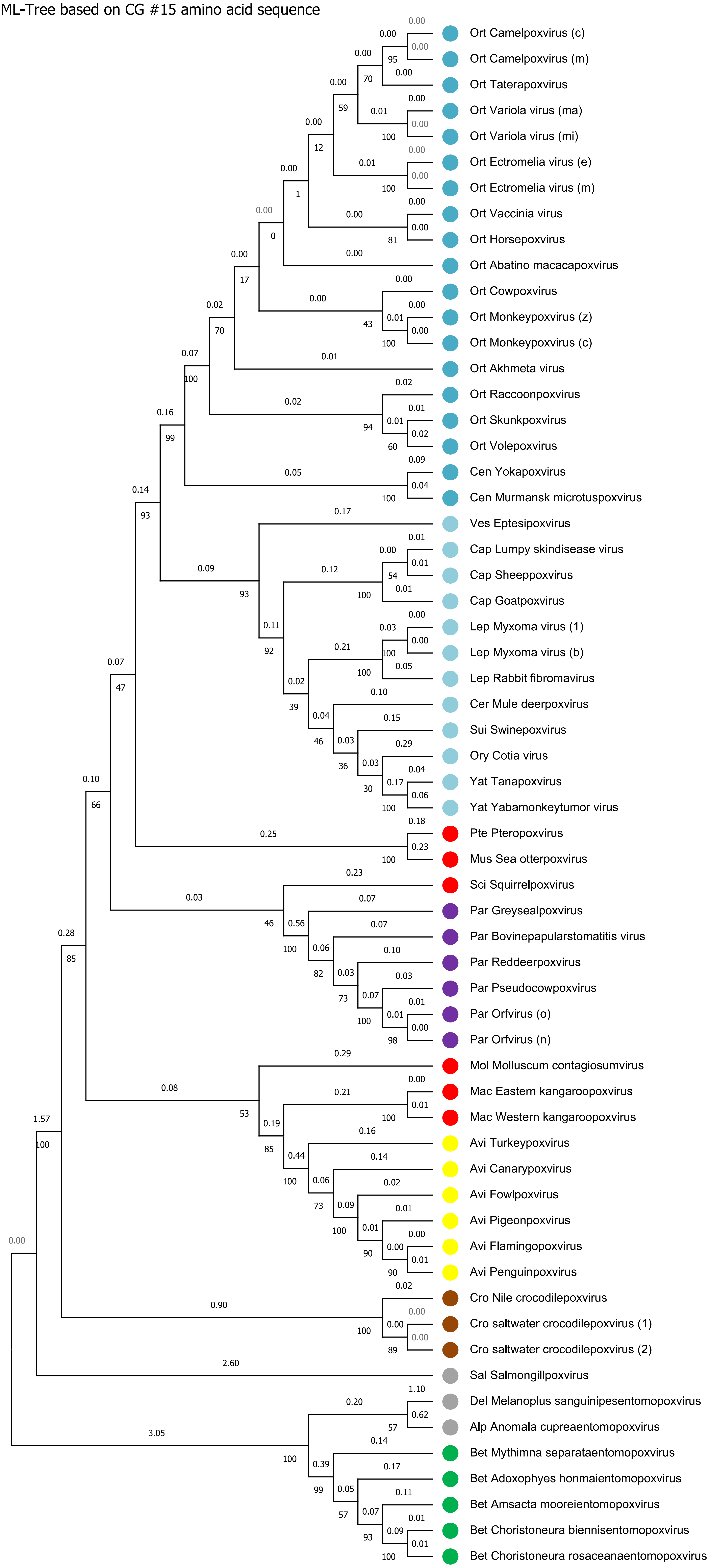

The ML-Tree based on CG #16 amino acid sequence

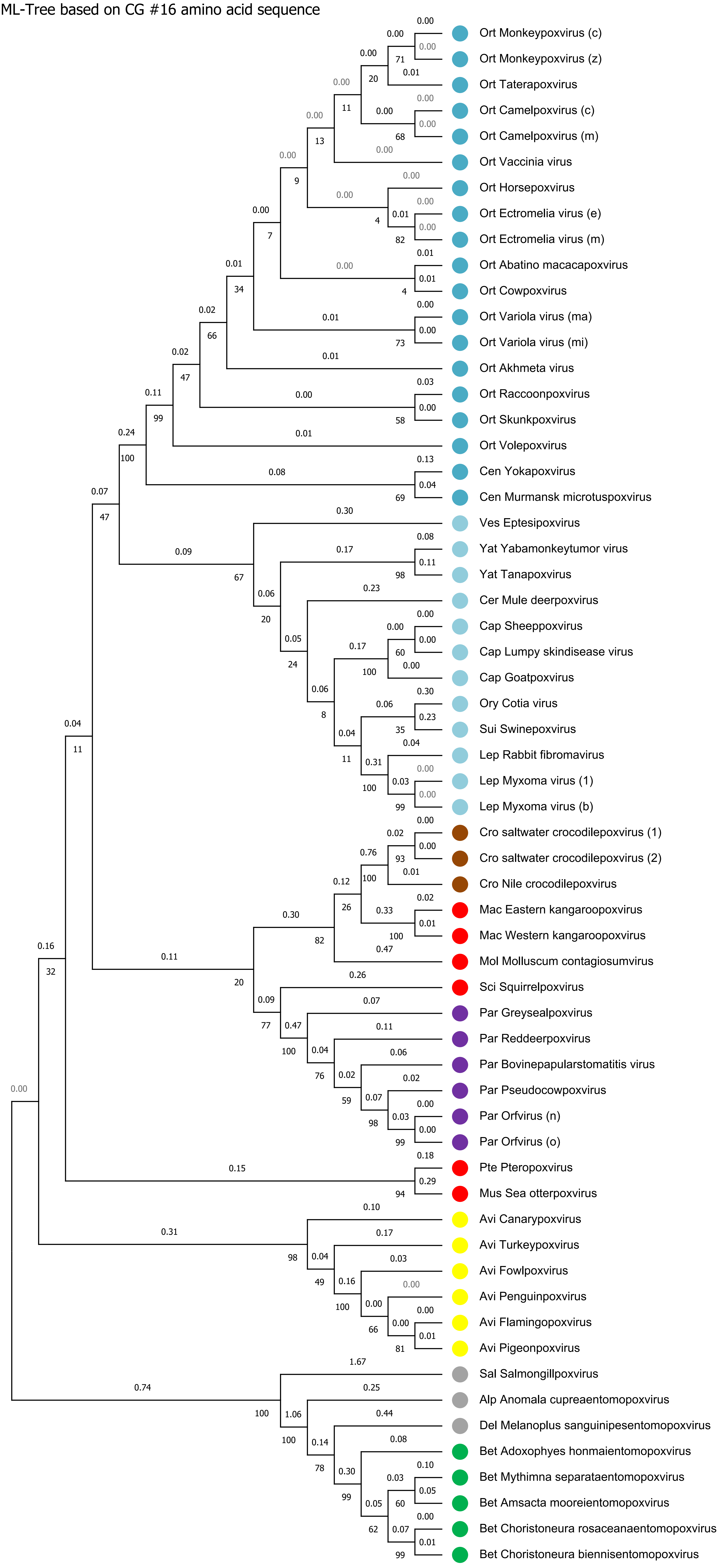

The ML-Tree based on CG #17 amino acid sequence

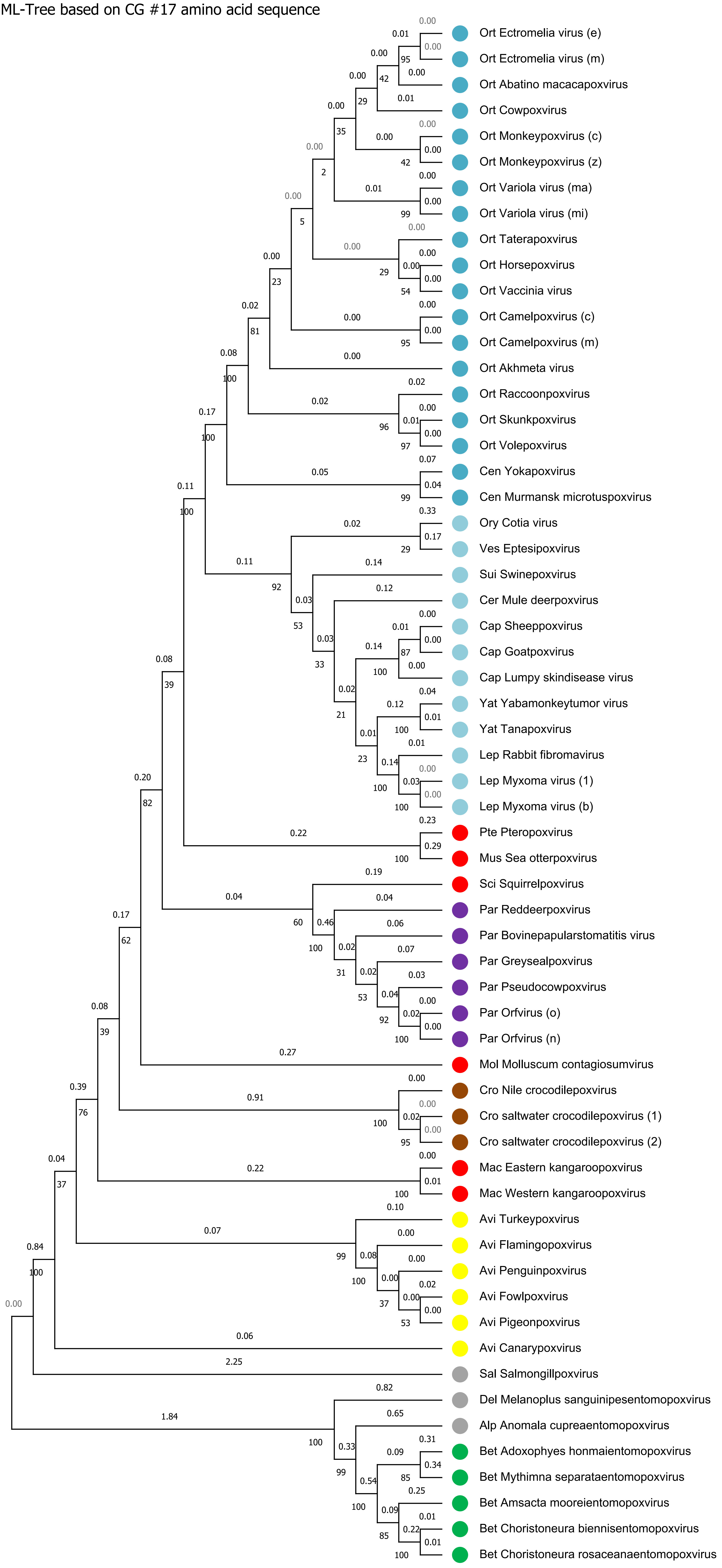

The ML-Tree based on CG #18 amino acid sequence

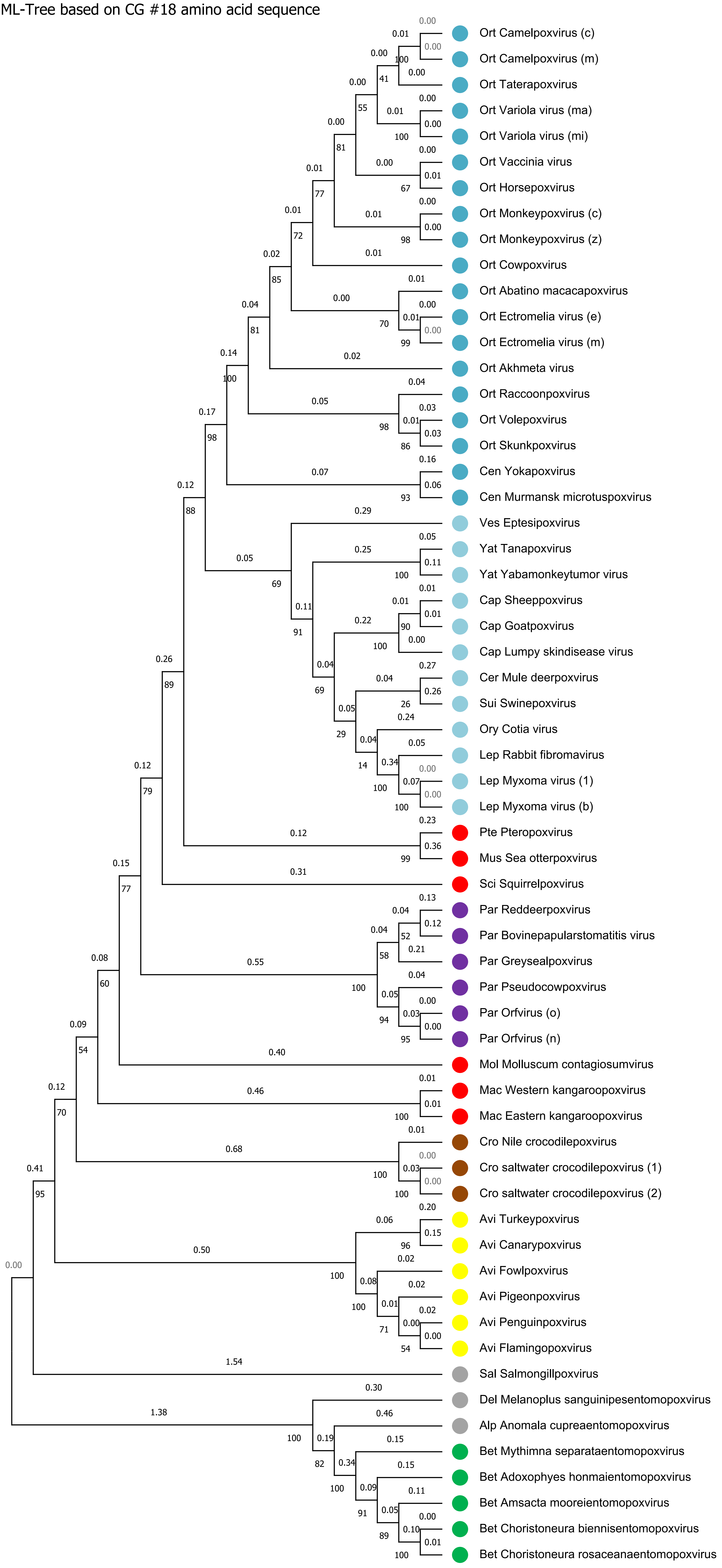

The ML-Tree based on CG #19 amino acid sequence

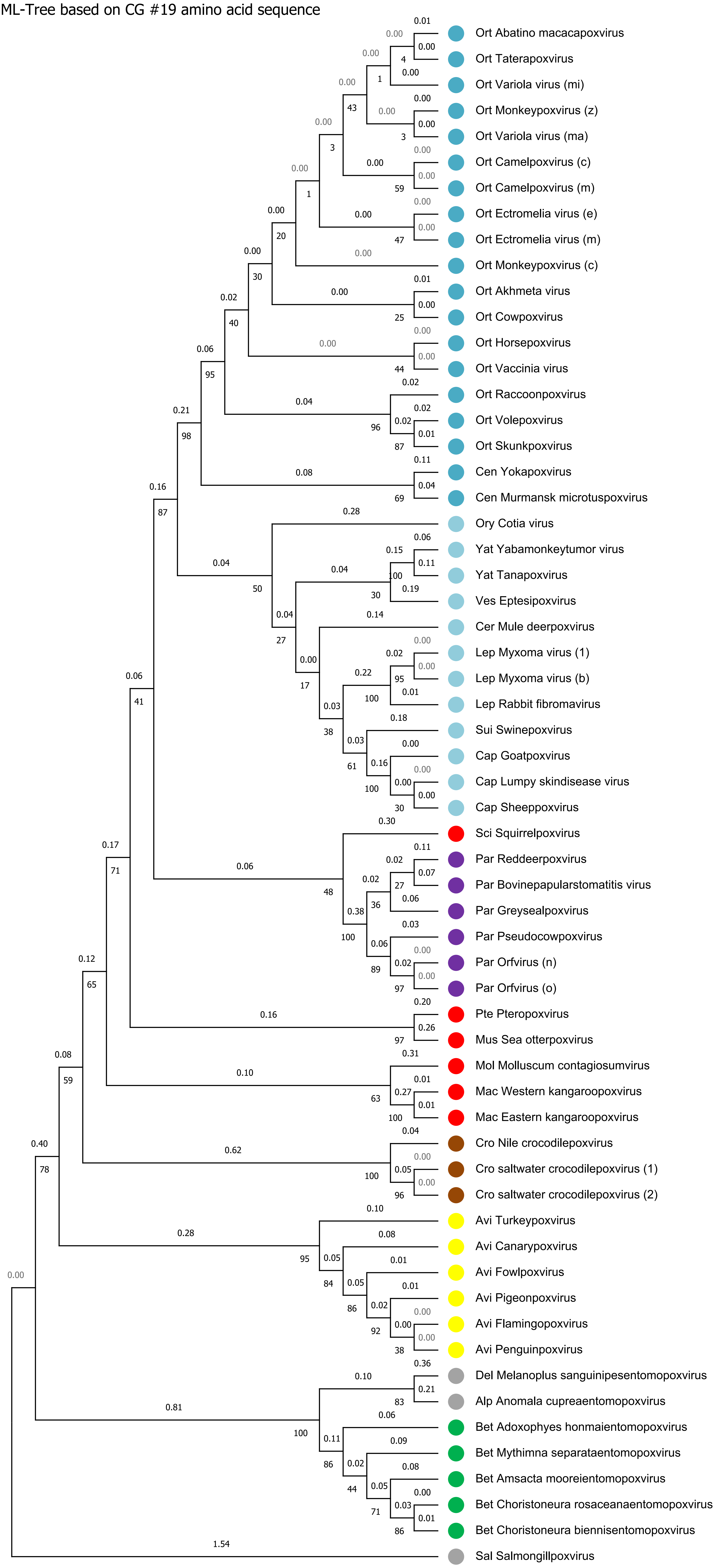

The ML-Tree based on CG #20 amino acid sequence

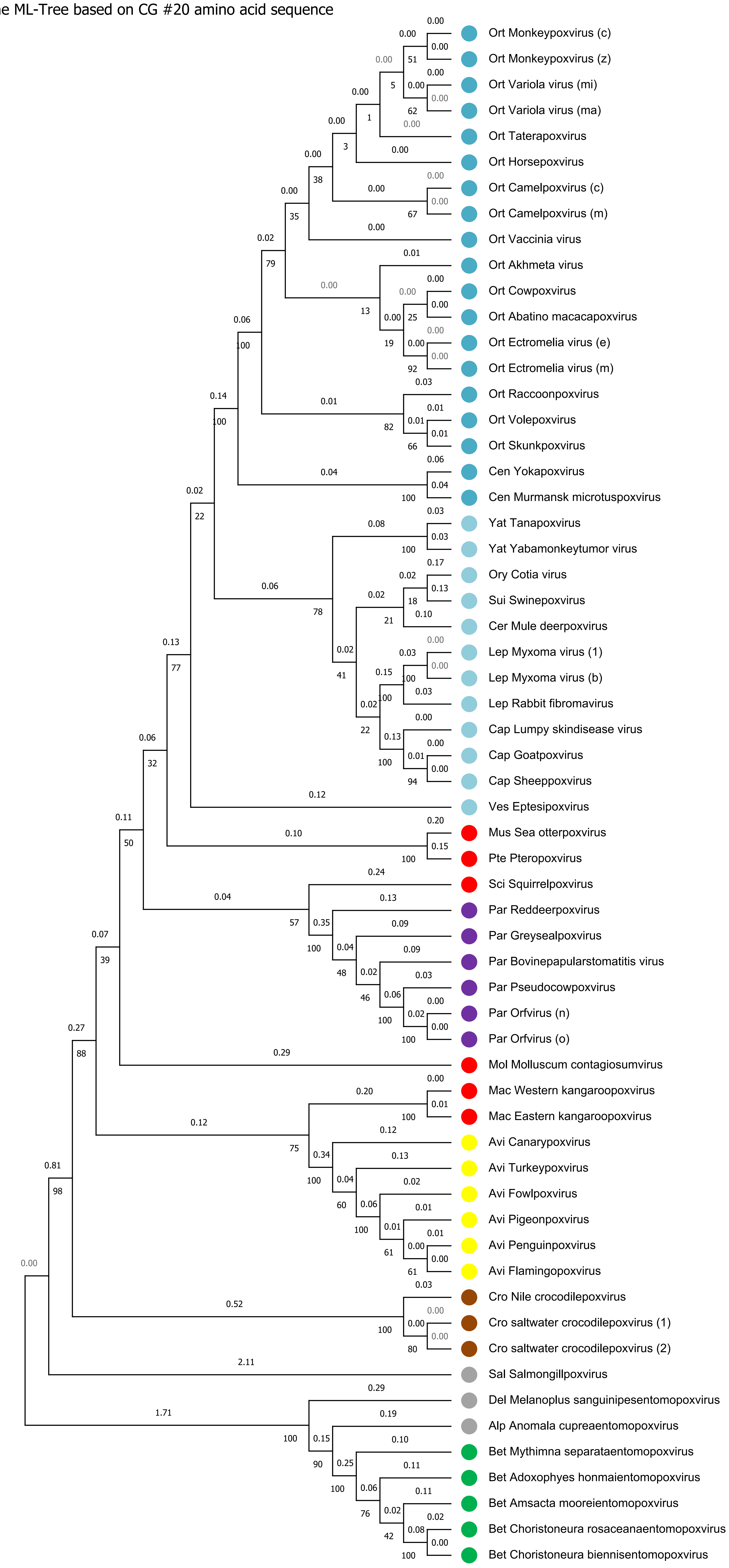

The ML-Tree based on CG #21 amino acid sequence

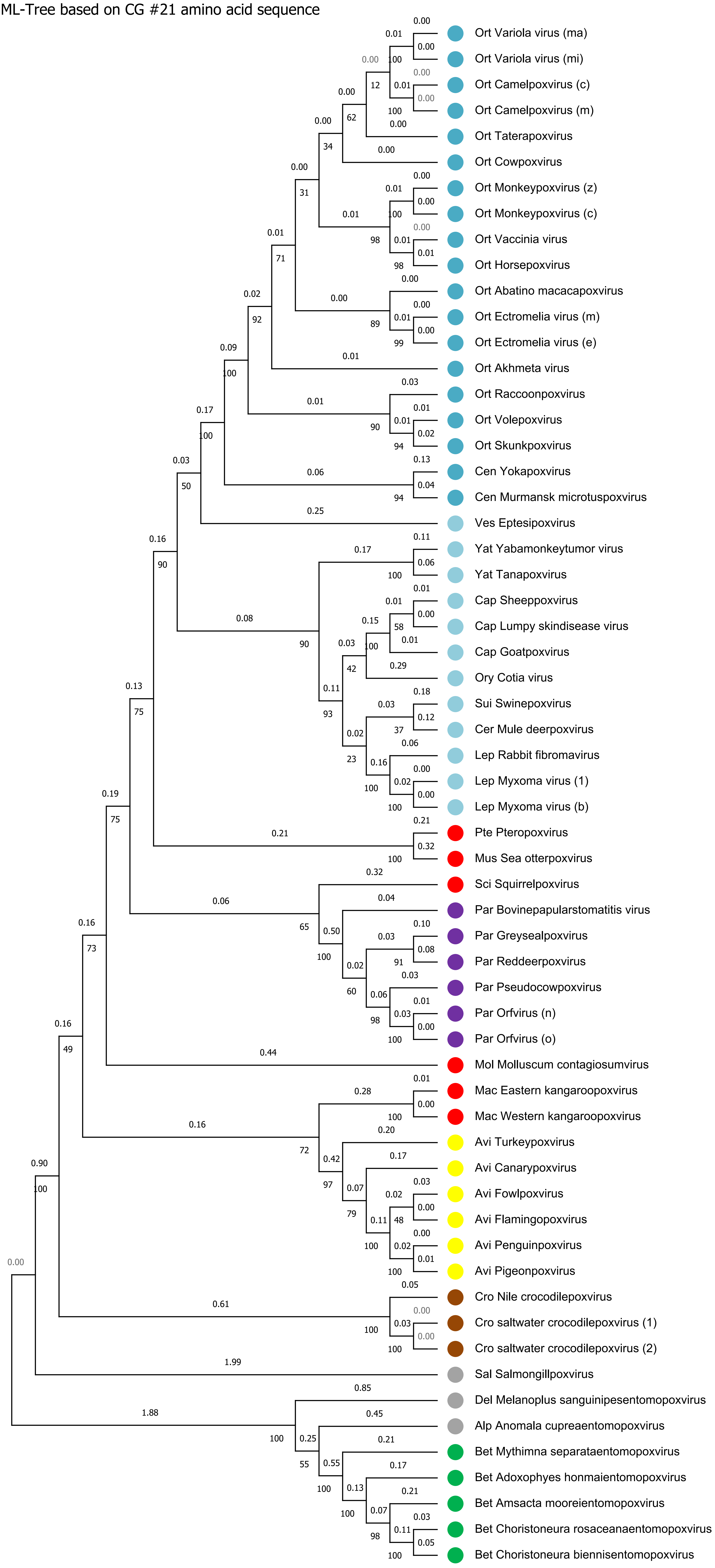

The ML-Tree based on CG #22 amino acid sequence

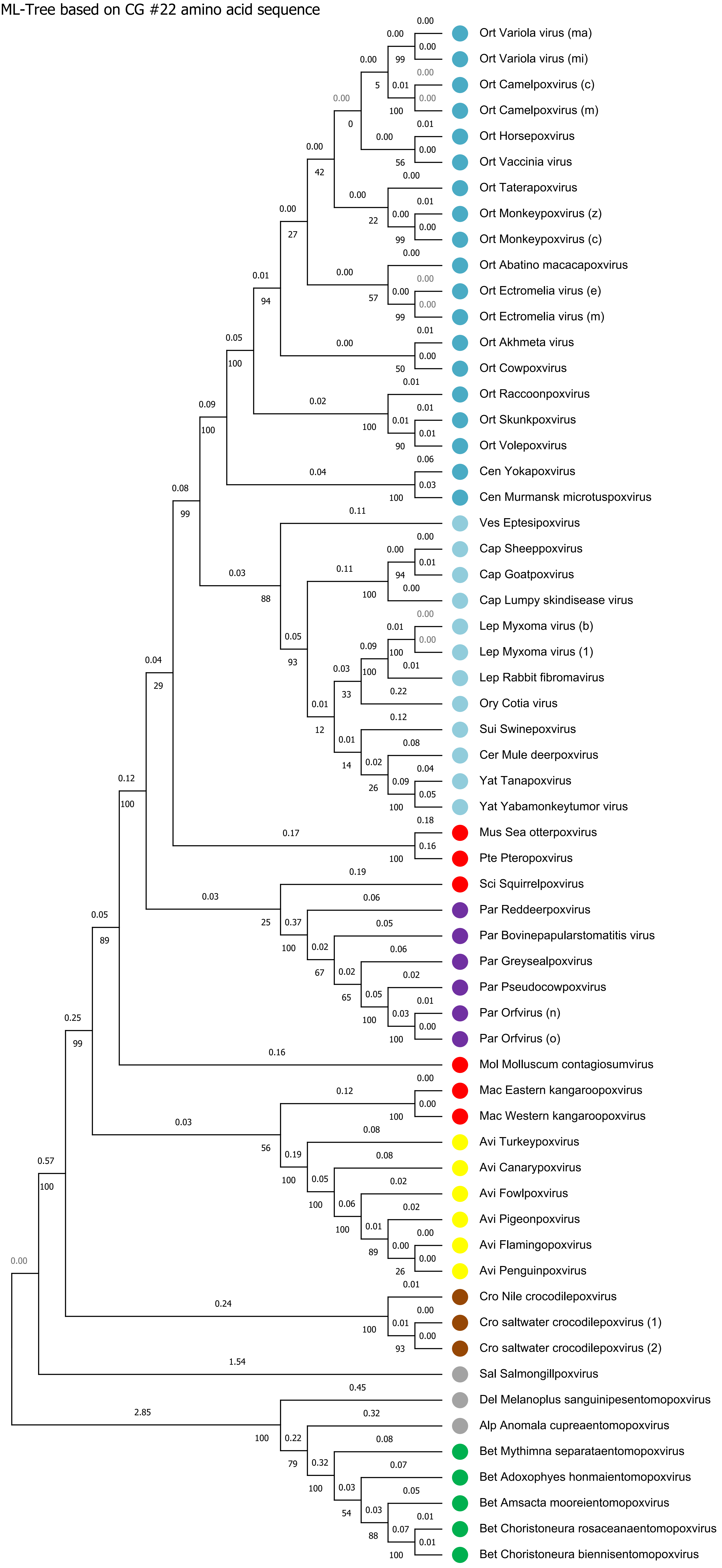

Supplement: Supplementary data 8 [file mmc8.pdf]
